# Equivalent DNA methylation variation between monozygotic co-twins and unrelated individuals reveals universal epigenetic inter-individual dissimilarity

**DOI:** 10.1186/s13059-020-02223-9

**Published:** 2021-01-05

**Authors:** Benjamin Planterose Jiménez, Fan Liu, Amke Caliebe, Diego Montiel González, Jordana T. Bell, Manfred Kayser, Athina Vidaki

**Affiliations:** 1 Department of Genetic Identification, Erasmus MC University Medical Center Rotterdam, Rotterdam, The Netherlands; 2 Key Laboratory of Genomic and Precision Medicine, Beijing Institute of Genomics, Chinese Academy of Sciences, Beijing, People’s Republic of China; 3 University of Chinese Academy of Sciences, Beijing, People’s Republic of China; 4 Institute of Medical Informatics and Statistics, Kiel University, Kiel, Germany; 5 University Medical Centre Schleswig-Holstein, Kiel, Germany; 6 Department of Twin Research and Genetic Epidemiology, King’s College London, London, UK

**Keywords:** Epigenetics, DNA methylation, Monozygotic twins, Inter-individual variation, Monozygotic twin discordance, Epigenetic drift, Metastable epialleles, Clustered protocadherins

## Abstract

**Background:**

Although the genomes of monozygotic twins are practically identical, their methylomes may evolve divergently throughout their lifetime as a consequence of factors such as the environment or aging. Particularly for young and healthy monozygotic twins, DNA methylation divergence, if any, may be restricted to stochastic processes occurring post-twinning during embryonic development and early life. However, to what extent such stochastic mechanisms can systematically provide a stable source of inter-individual epigenetic variation remains uncertain until now.

**Results:**

We enriched for inter-individual stochastic variation by using an equivalence testing-based statistical approach on whole blood methylation microarray data from healthy adolescent monozygotic twins. As a result, we identified 333 CpGs displaying similarly large methylation variation between monozygotic co-twins and unrelated individuals. Although their methylation variation surpasses measurement error and is stable in a short timescale, susceptibility to aging is apparent in the long term. Additionally, 46% of these CpGs were replicated in adipose tissue. The identified sites are significantly enriched at the clustered protocadherin loci, known for stochastic methylation in developing neurons. We also confirmed an enrichment in monozygotic twin DNA methylation discordance at these loci in whole genome bisulfite sequencing data from blood and adipose tissue.

**Conclusions:**

We have isolated a component of stochastic methylation variation, distinct from genetic influence, measurement error, and epigenetic drift. Biomarkers enriched in this component may serve in the future as the basis for universal epigenetic fingerprinting, relevant for instance in the discrimination of monozygotic twin individuals in forensic applications, currently impossible with standard DNA profiling.

**Supplementary information:**

**Supplementary information** accompanies this paper at 10.1186/s13059-020-02223-9.

## Background

Compared to its genomic counterpart, human epigenomic inter-individual variation remains relatively unexplored. Particularly for cytosine-guanine dinucleotide (CpG) methylation, the currently known sites of substantial inter-individual variation are restricted to a limited number, as most are completely unmethylated or methylated across healthy populations [1, 2]. Via epigenome-wide association studies (EWAS) numerous traits have been associated to epigenetic variation; trait-associated CpGs, however, tend to display small effect sizes [3, 4]. The main drivers of inter-individual DNA methylation variation identified so far are genetics, sex, cell type/tissue, environment, and aging [5–7]. The latter includes both the epigenetic clock, i.e., the direct association between CpG methylation and age across individuals, and the epigenetic drift, defined as individual-specific accumulation of stochastic and environmental changes over time [8, 9].

On this note, it was widely popularized that healthy monozygotic (MZ) twins sharing sex, age, and practically identical genomes display indistinguishable methylomes at a young age, while at an older age, differential exposures to environmental factors promote methylation divergence over time (epigenetic drift) [10, 11]. As exceptions to the above, developmental stochastic mechanisms promoting epigenetic variation do exist; for example, X-inactivation or genomic imprinting [12, 13]. Moreover, metastable epialleles were recently identified, presenting methylation levels that are stochastically established during early development, but faithfully passed on across cell divisions and differentiation [14–16]. In practice, however, metastable epiallele variation in MZ co-twins is limited due to the phenomenon of twin super-similarity; namely, a stochastic setting of methylation states prior to the twinning process results in identical methylation profiles for both twins [17].

Some attempts to map epigenome-wide variation via twin models have been previously reported. Particularly successful by employing both MZ and dizygotic (DZ) twins, ACE models decompose the total variance into an additive genetic component (A), a common environmental component (C), and an unshared environmental component (E). The E component encompasses both intra-individual measurement error and inter-individual stochastic biological variation since both qualify as non-genetic influence unshared between twins [5, 6]. As a result, CpG sites displaying no biological variation and hence only subject to measurement error display relative E components close to 1. This turns out to be a problem as generally ACE models are fitted to every CpG, disregarding whether they present inter-individual variation or not. Separating between measurement error and genuine stochastic inter-individual epigenetic variation persists as a long-standing challenge in epigenetics.

Integrating all this information, if the prevalence of stochastic epigenetic inter-individual variation surpassing intra-individual measurement error is frequent enough, this could serve as a source of variation that promotes divergence between any two individuals including MZ twins. Hence, we hypothesized that such a universal stochastic epigenetic component exists and can be isolated following a MZ twin study design. This concept of universal epigenetic variation is the opposite to rare epigenetic variation that only affects a small subset of MZ twin pairs, for example, due to pathological discordance. Assuming limited genetic influence, absolute methylation differences of CpGs susceptible to inter-individual stochastic variation are expected to be similarly distributed between MZ co-twins and unrelated pairs of individuals. However, to avoid other stochastic components, whose predominance increases with age such as epigenetic drift, we decided to direct our analysis to MZ twins of young age. That way, we also expected to enrich for post-twinning stochastic DNA methylation differences having originated during embryonic development and early life rather than changes due to epigenetic drift.

Based on these hypotheses, the objective of this study was to identify CpGs that display inter-individual methylation variation equivalent between young co-twins and unrelated individuals that cannot be explained by epigenetic drift and/or measurement error. Given that such a universal stochastic component is expected to generate inter-individual variation for every pair of individuals including MZ twins, we envision that it could serve as the basis of an epigenetic fingerprint, relevant for individualizing MZ twins in forensic applications in the future, although further research is necessary. To address the different questions posed throughout the manuscript, we integrated 11 publicly available datasets. We considered data derived from two methods: the Illumina Infinium HumanMethylation450K Beadchip array (450K), covering > 450,000 CpG sites and the whole genome bisulfite sequencing (WGBS), currently considered as the gold standard in methylomics. Among them, we included MZ twins, unrelated individuals, longitudinal samples, and technical replicates obtained from whole blood, adipose tissue, and post-mortem tissues.

## Results

### Discovery of equivalently variable (ev)CpGs

In search for CpGs displaying similar variation between MZ co-twins and unrelated individuals, an epigenome-wide discovery phase was implemented in 450K CpG methylation data derived from whole blood of 426 MZ twin pairs sampled at age 18 (*dataset-A*, Table 1) [5]. Described thoroughly in Additional file 3: Supplementary methods, we firstly implemented strict quality control and preprocessing (Additional file 1: Figures S1–5). For example, we excluded SNP-containing, cross-reactive, low-quality, and X,Y-chromosomal probes, controlled for predicted cell composition differences (Additional file 1: Figure S5) and employed three different normalization methods in parallel (Additional file 1: Figure S6). Secondly, given that CpGs with no biological variation display only measurement error and are also expected to show equivalent co-twin and inter-individual variation, we pre-selected variably methylated CpGs using empirical cut-offs for inter-individual variation (inter-quantile range (IQR) > 0.07) and replicability (intra-class correlation coefficient (ICC) > 0.37) [27].

**Table 1:** Description of the 13 DNA methylation datasets employed in this study

Dataset	Dataset	Technology	Tissue	Number	Ethnicity	Female (%)	Mean age (years)
**A**	E-risk [5]	450K	Whole blood	426 MZ twin pairs	British	48.6	≈ 18
**B**	Danish twin cohort [18]	450K	Whole blood	146 MZ twin pairs	Danish	47.9	48.4
**C1**	Zhang et al. [19]	450K	Whole blood	10 MZ twin pairs	Chinese	40.0	41.3
**C2**		450K	Whole blood	1 MZ twin pair and 6 individuals	Chinese	37.5	29.3
**D**	Shi et al. [20]	450K	Whole blood	48 individuals	Chinese	39.6	9.04
**E**	NSPHS [21]	450K	Whole blood	727 individuals	Swedish	53.0	47.4
**F**	TwinsUK [22]	450K	Whole blood	328 MZ twin pairs	British	100	57.9
**G**	ENID [16]	450K	Whole blood	240 individuals	Gambian	48.6	≈ 2
**H**	Lokk et al. [23]	450K	17 post-mortem somatic tissues	4 individuals	Estonian	25.0	51.8
**I**	TwinsUK [24]	450K	Adipose tissue	97 MZ twin pairs	British	100	N/A
**J**	Bollepalli et al. [25]	450K	Adipose tissue	19 individuals	Finnish	63.1	35.2
**K1**	TwinsUK [26]	WGBS	Whole blood	7 MZ twin pairs	British	100	59.1
**K2**		WGBS	Adipose tissue	7 MZ twin pairs	British	100	60.7

**Fig. 1:**
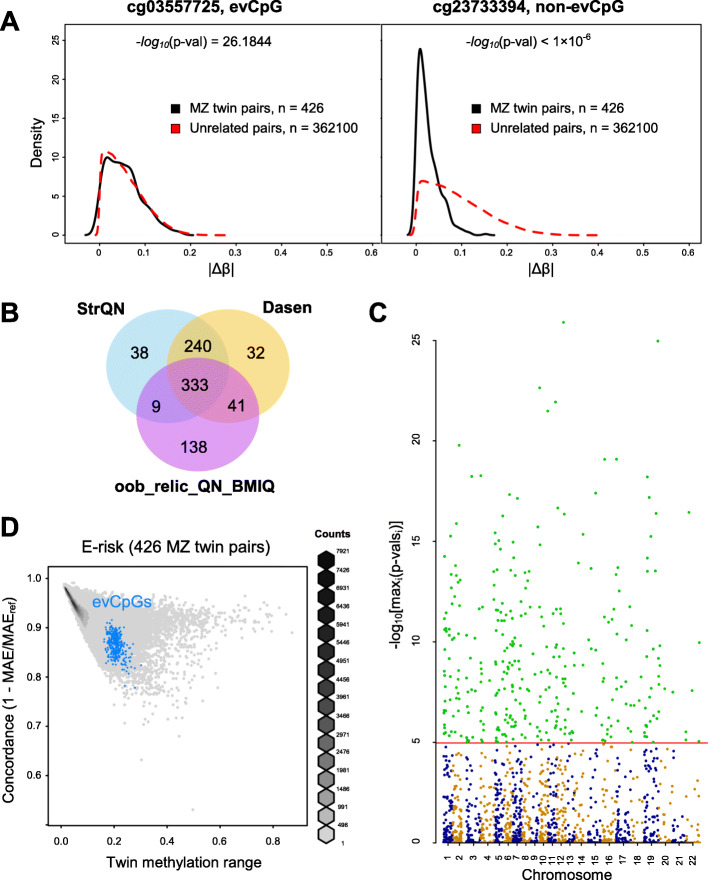
Discovery of evCpGs. **a** |Δ*β*|^MZ twin pairs^ and |Δ*β*|^unrelated pairs^ distributions in an example of an evCpG (left) and a variably methylated non-evCpG (right). Similarity *p* values were obtained via equivalence testing with a two one-sided tests procedure. **b** Venn-Euler diagram displaying significant hits across three normalization methods (StrQN, dasen and oob_RELIC_QN_BMIQ), where the intersection between the three sets corresponds to evCpGs. **c** Manhattan plot displaying CpG significance across chromosomes (odds and even represented either in blue or orange), where evCpGs are highlighted in green. For each CpG, we used the maximal *p* value across three normalization methods. **d** Agreement between twins measured as concordance plotted against methylation range (see Additional file 3: Supplementary Methods for details), where evCpGs are highlighted in blue

Thirdly, for the remaining 4652 variably methylated probes, we estimated MZ co-twin and inter-individual variation by computing absolute methylation differences between MZ twin pairs and all combinations of unrelated MZ twin individuals, respectively. We then employed statistical inference under the scheme of equivalence testing to test whether these methylation differences are similarly distributed (Fig. 1a). This approach identified 333 equivalently variable CpGs (evCpGs) between co-twins and unrelated individuals that were statistically significant across all three normalization methods employed (Fig. 1b, c, Additional file 1: Figure S6, Additional file 2). To ensure that our statistical approach has not been compromised due to the artificial exploration of all the unrelated individual pairs based on the MZ twin dataset, we performed additional verification tests (Additional file 3: Supplementary Methods, Additional file 1: Figure S7). As expected, while most CpGs covered in the Illumina 450K array tend to present low inter-individual variation concordant between MZ co-twins, evCpGs display substantial co-twin and inter-individual variation (Fig. 1d, Additional file 1: Figure S8).

### evCpG variation versus measurement error

**Fig. 2:**
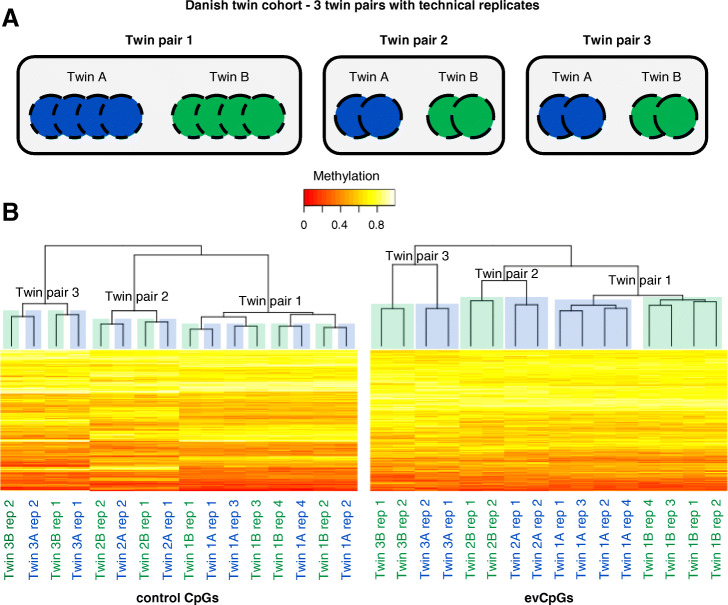
Superior evCpG variation in MZ twins compared to technical replicates. **a** A subset of the Danish twin cohort including 3 twin pairs with technical replicates. **b** Heatmap with unsupervised hierarchical clustering employing 329 out of the 333 evCpGs and equal number of genetically influenced negative control probes. Technical replicates within MZ twin pairs cluster together for evCpGs, unlike negative control probes that cluster per microarray chip batch

Within our pipeline, given that the exclusion of CpGs subject only to measurement error relies heavily on the correct setting of empirical thresholds, it was of importance to prove that our selected evCpGs indeed displayed a level of variation larger than the measurement error. Towards this goal, we firstly checked that the distributions of 450K array technical measures, including number of beads per probe, high detection *p* value and ICC [27], were similar between evCpGs and non-significant CpGs (Additional file 1: Figure S9, see Additional file 3: Supplementary methods for details). This analysis confirmed that our pipeline did not just deliberately enrich for CpGs displaying sub-standard technical performance in the microarray. Secondly, employing independent data from the Danish Twin Registry (*dataset-B*, Table 1) [18], we confirmed that evCpG variation was indeed significantly larger in MZ co-twins than in technical replicates (Additional file 1: Figure S10; *p* value = 4.3 × 10^−41^, Kolmogorov-Smirnov). Moreover, evCpG variation was large enough to successfully separate technical replicates into clusters within each twin pair unlike a set of equal number of genetically influenced CpGs acting as negative controls, extracted from previously reported methylation quantitative trait loci (mQTL) (Fig. 2) [7]. This was also true on a single twin pair with technical replicates in the dataset of Zhang et al. (dataset-C1) (Additional file 1: Figure S11A) [19].

### Short-term time stability of evCpGs

**Fig. 3:**
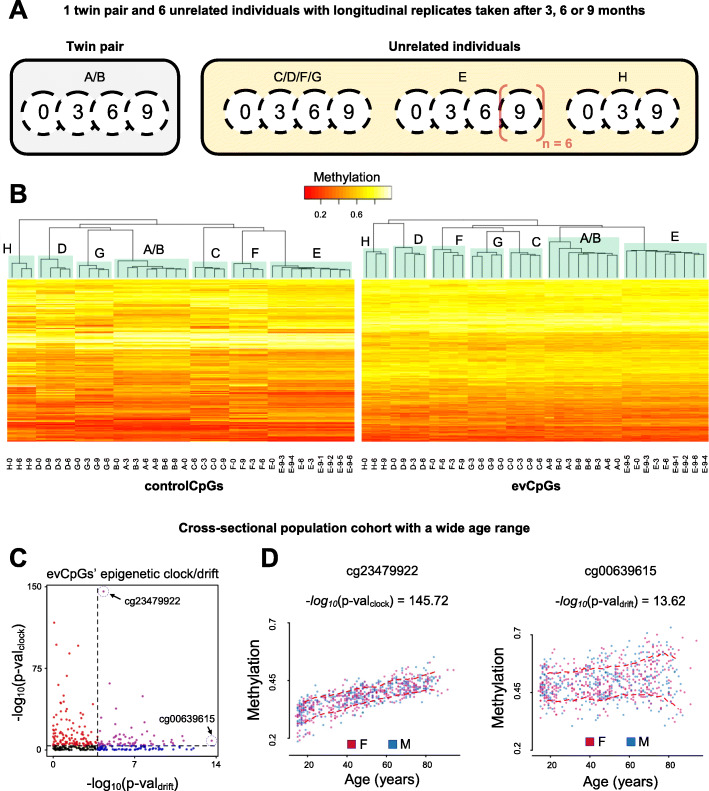
Time-stability of evCpGs. **a** A subset of the dataset of Zhang et al that includes short-term longitudinal replicates obtained 3, 6, and 9 months apart. **b** A heatmap with unsupervised hierarchical clustering employing 296 out of the 333 evCpGs and equal number genetics-dependent negative control probes. Longitudinal replicates cluster together like negative control probes. **c** Epigenetic clock and drift of evCpGs. –log_10_(*p* values) for association are plotted against –log_10_(*p* values) for heteroscedasticity, with respect to age. The colors symbolize significance for association (red), heteroscedasticity (blue) or both (purple). **d** DNA methylation levels plotted against age for example evCpGs highlighted in **c**; males are represented in blue and females in pink, while mean bin methylation ± sd (window length = 10 years, offset = 2 years) are highlighted in red

Once proven that the observed DNA methylation differences at evCpGs were greater than measurement error and to shed light on their hyper-variability, we examined whether evCpG methylation levels behaved erratically in time. To do so, we moved on to a second subset of the dataset from Zhang et al. (*dataset-C2*, Table 1) including multiple samples from 6 unrelated individuals and one twin pair taken up to 9 months apart. Via hierarchical clustering, we observed that longitudinal replicates of unrelated individuals tended to cluster together per individual; the single twin pair though could not be separated into its longitudinal replicates (Fig. 3a). From these findings, we conclude that evCpG methylation in whole blood is relatively stable in time as temporal variation is unable to overcome inter-individual variation at least regarding the tested timescale. For a more quantitative view on short time stability of evCpGs, we also provide the temporal ICC distributions obtained from estimates by Flanagan et al. [28] (Additional file 1: Figure S11B).

### Epigenetic clock/drift of evCpGs

Once evCpG short-term stability was confirmed and given that we used adolescent MZ twins in the initial discovery phase rather than newborns, we next investigated whether methylation divergence at evCpGs could be solely explained as a result of aging in the timescale including infancy and adolescence. Though a longitudinal study design would allow us to identify CpGs susceptible to aging at an individual level, we here focused on population level changes in DNA methylation (e.g. universal variation). Under a cross-sectional design, the epigenetic clock and epigenetic drift can be observed as a direct association or increased variation with age, respectively.

On this note, we first examined the cross-sectional dataset of Shi et al. (*dataset-D*, Table 1) [20] containing 48 children aged from 6.4 to 14.6 years. From the set of evCpGs (*n* = 333), only one (0.3%) showed a direct association between age and DNA methylation (i.e., epigenetic clock), while two (0.6%) showed age-associated increase in methylation variation (i.e., epigenetic drift) (Additional file 1: Figure S12A). Motivated by the absence of strong evidence of evCpG aging effects in this narrow period between late childhood and adolescence, we moved on to the cross-sectional dataset of 727 individuals of the Northern Sweden Population Health Study [21] with a wider age interval ranging from 14 to 94 years (*dataset-E,* Table 1). Out of 331 evCpGs available in this dataset, 122 (36.9%) showed only age-associated effects (i.e., epigenetic clock), 63 (19.0%) showed only an age-associated increase in variation (i.e., epigenetic drift), while 67 (20.2%) showed both (Fig. 3c, d). Further confirming the influence of epigenetic drift on evCpGs in a broader timeframe, observed absolute methylation differences in the older TwinsUK cohort (*dataset-F*, Table 1) [29] were significantly higher than in the adolescent twins used for evCpG discovery (Additional file 1: Figure S12C; *p* value = 7.8 × 10^−144^, Kolmogorov-Smirnov). Thus, evCpG methylation is subject to epigenetic drift at large timescales but its influence in the period between late childhood and adolescence seems to be minor. To trace back the source of inter-individual variation, we searched for data from even younger cohorts. Moving on to a dataset consisting of 2-year-old Gambian children (*dataset-G*, Table 1), strong inter-individual evCpG variation was also evident (Additional file 1: Figure S12B). If strong deterministic genetic effects were to be predominant on evCpG variation, these would fuel inter-individual, but not co-twin variation; hence, the discovery condition of equivalence would not be met. For this reason, it is improbable that strong genetic effects are a major contribution to the inter-individual variation of evCpGs in the Gambian children cohort. Together with the lack of strong evidence for epigenetic drift in the children from *dataset-D*, we conclude that epigenetic drift cannot be fully responsible for the observed discordance in 18-year-old MZ twins. Thus, evCpG stochastic variation likely originates in embryonic development and/or early life but is amplified over a lifetime via epigenetic drift.

### evCpG methylation in other tissues

As tissue type is known to be a strong driver of DNA methylation variation, we aimed to assess whether this was also the case for evCpGs that we had identified in whole blood. In addition, after having discarded genetic effects, observing a strong correlation between tissues would serve as convincing evidence for the establishment of evCpG methylation in early development, similarly to metastable epialleles.

In order to achieve sufficiently large numbers of different tissues per individual, we made use of a panel including 17 different post-mortem somatic tissues (*dataset-H*, Table 1) [23]. The results from multi-dimensional scaling (MDS) analysis did not reveal the formation of clusters per individual in the first two principal components, indicating that evCpGs are subject to strong variation between tissues (Additional file 1: Figure S13C). This effect percolated even in the set of genetically influenced control CpGs, for which inter-individual variation was pushed back to the second principal component (Additional file 1: Figure S13B). This could be due to reduced data quality due to the post-mortem nature of the tissue or simply that mQTL discovered in the blood do not apply to other tissues.

**Fig. 4:**
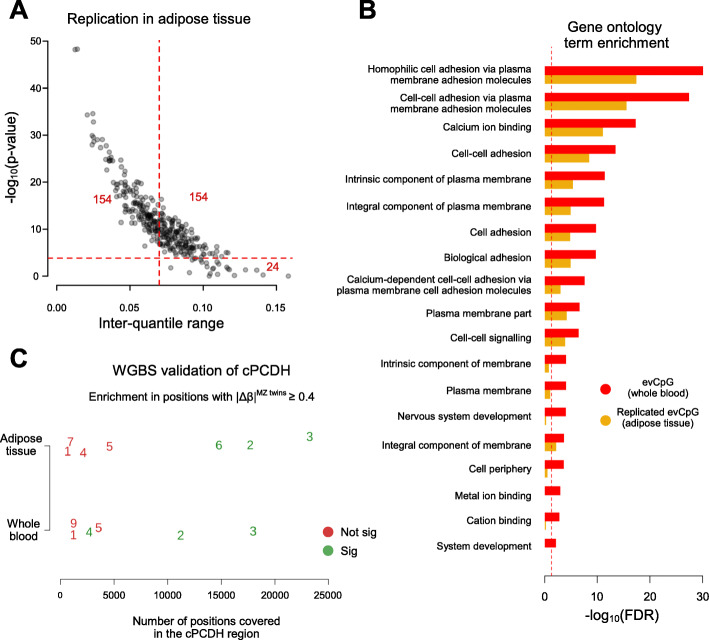
evCpG variation in other tissues. **a** Replication on 332 out of 333 evCpGs in adipose tissue. –log_10_(equivalence *p* value) is plotted against IQR. Threshold lines represent IQR filter of 0.07 and Bonferroni significance. Numbers in red highlight the number of hits in a given sector. **b** Gene ontology (GO) term enrichment of evCpGs (red) and the replicated subset in adipose tissue (orange). The threshold line indicates false discovery rate of 0.05. **c** Whole genome bisulfite sequencing validation on epigenetic discordance between MZ twins in the *cPCDH* region using data of the TwinsUK cohort. Here, we observe the dependence between significance and coverage in the *cPCDH* region. Twin pairs 1 to 5 were assayed in both tissues. For twin pair 8 in whole blood, no sites were shared between twins and hence, enrichment could not be performed. Twin pairs are highlighted in green when a significant enrichment in |Δ*β*| ≥ 0.4 on the *cPCDH* loci with respect to the background is observed.

Setting aside the idea of co-methylation across tissues, we aimed to investigate whether the stochastic behavior of evCpGs itself was beyond whole blood. Saliva and buccal cells are the second most employed tissues in epigenomic datasets; however, suitable large MZ twin datasets are not publicly available. Therefore, we sought to replicate the effect of evCpGs in subcutaneous adipose tissue, a relatively homogenous tissue composed primarily by adipocytes, with only a minor component of endothelial cells and macrophages [30]. Towards this goal, we employed adipose tissue data from the TwinsUK cohort that includes 97 MZ twin pairs (*dataset-I*, Table 1), in which we replicated a total of 154 (46%) of the evCpGs (Fig. 4a). Moreover, we also confirmed short-term temporal stability of the replicated evCpGs in this tissue via hierarchical clustering on longitudinal replicates derived from obese individuals subject to weight intervention (*dataset-J*, Table 1, Additional file 1: Figure S14A). For a more quantitative interpretation, we also estimated temporal ICCs and examined their distributions (Additional file 1: Figure S14B). In conclusion, almost half of the identified evCpGs display stochastic variation in both the blood and adipose tissue.

### Functional annotation of evCpGs

In order to investigate the functional role of evCpGs, we firstly sought for insights in the sequence context of evCpGs. We performed DNA motif enrichment analysis, but no motif showed a large and statistically significant odds ratio (Additional file 1: Figure S15, S16A). Looking closer in the sequences surrounding evCpGs, we observed a significantly diminished [G + C] content (*p* value = 1.1 × 10^−8^, Mann-Whitney *U* test, Additional file 1: Figure S16B), consistent with variable methylation as previously reported [31]. However, we did not observe a significant decrease in CpG island-associated CpGs (Additional file 1: Figure S17B).

To uncover other putative functional roles of evCpGs, we consulted a wide range of public databases and annotations. Examining the evCpG relationship to nearby genes, we noted statistically significant profile divergence compared to the background (*p* value_func_ = 1 × 10^−5^; *n*_bootstrap_ = 100,000; Fisher’s exact test), driven by an enrichment in CpGs not associated to genes and CpGs associated to 1st Exon and within 1500 bp range from transcription starting site (TSS_1500_), as well as a depletion in CpGs associated to 5′-untranslated regions (5′-UTR) and within 200 bp range from transcription starting site (TSS_200_) (Additional file 1: Figure S17A). Altogether, this suggests that evCpGs tend to lie outside important regions for gene regulation. To further test this concept, we made use of the 15-state ChromHMM model from peripheral blood mononuclear cell (PBMC) [32], which is a Hidden Markov Model (HMM) representation of the genome based on the patterns of post-translational modifications of histones and DNA methylation that segments different genomic loci into 15 types of chromatin regulation. Confirming our prior notes, we observed statistically significant divergence in chromatin states between evCpGs compared to the background (*p* value = 1 × 10^−5^; *n*_bootstrap_ = 100,000; Fisher’s exact test). More specifically, a strongly significant increase in heterochromatin in addition to both strongly and weakly polycomb repressed states were observed together with a statistically significant depletion in active TSS flanking regions and actively transcribed states (Additional file 1: Figure S18). Finally, after confirming generally low mRNA expression in blood for evCpG-associated genes compared to a wide panel of tissues from the genotype-tissue expression (GTEX) database (Additional file 1: Figure S19), we conclude that evCpGs tend to lie outside functional genomic regions in blood.

Moreover, we examined potential enrichment in imprinted regions and metastable epialleles, as the literature has highlighted these regions as potential subjects to stochastic methylation variation. We found that evCpG-associated genes were not significantly enriched in imprinted genes (*p* value = 0.8436, Fisher’s exact test) in contrast with previously reported metastable epiallele-like CpGs [33], which showed almost a 10-fold enrichment (*p* value = 3.87 × 10^−8^, Fisher’s exact test). Besides, looking into previously discovered mQTL in the blood of adolescents [7], we found a 5-fold depletion with respect to a background composed by the 4319 variably methylated CpGs which were not included in the evCpG set due to missing co-twin variation and potential influence by genetics (*p* value = 1.34 × 10^−43^, Fisher’s exact test). This was expected since genetic effects were expected to promote inter-individual, but not co-twin variation. Also, utilizing the EWAS Atlas database [3], we further tested for enrichment in previously reported phenotypic associations. A significant enrichment was present not only in probes associated to aging as expected, but also to other traits, such as gender, ancestry, respiratory allergies, some syndromes caused by mutations in the epigenetic machinery, and others associated with pregnancy and early childhood (Additional file 1: Figure S20).

Furthermore, we performed gene ontology (GO) term enrichment analysis. Our results on evCpGs support a putative relationship to development, as evCpG-associated genes are significantly enriched in (nervous) system development processes (Fig. 4b). However, the most striking result was the strong enrichment in “homophilic cell adhesion via plasma membrane adhesion molecules” terms, explained by a large number of clustered protocadherins (*cPCDH*)-associated CpGs. From the total evCpGs, almost 5% collocated with *cPCDHs* in a 1-Mb stretch on chromosome 5 (16 out of 333 CpGs, *p* value = 2.8·10^−16^, Fisher’s exact test). Such significant GO term enrichment and collocation was also observed in the replicated set in adipose tissue (12 out 154 CpGs, *p* value = 3.7 × 10^−17^, Fisher’s exact test). *cPCDHs* are three combinatorial gene clusters (respectively, *α*, *β*, and *γ*), coding for homophilic membrane receptors whose promoter choice is established during early embryonic neurodevelopment via stochastic methylation [34–36]. cPCDHs are involved in the self-recognition of extending neurons by supplying a set of unique membrane receptor identifiers via combinatorial epigenetic silencing of promoters, key to avoid the formation of self-synapses, and hence, short-circuits in the neuronal circuitry (e.g. self-avoidance). Also, functions concerning post-natal same-lineage preferential synapsis formation in neurons have been reported [37–40]. Little is known about the epigenetic behavior of *cPCDHs* in whole blood or adipose tissue, although *cPCDHs* are not expressed in either of them (Additional file 1: Figure S19). Finally, to search for other putative clusters of evCpGs collocated in the genome, we performed an unbiased positional enrichment, finding 11 other smaller but significantly enriched loci in a 1-kb window centered around evCpGs (Additional file 2).

### Validation of clustered protocadherins across technologies

Aiming at replicating the observed methylation differences at *cPCDHs* on a different technological platform, we used publicly available whole genome bisulfite sequencing (WGBS) data from whole blood and adipose tissue of MZ twin pairs (*dataset-K1* (*n* = 7) and *dataset-K2* (*n* = 7)), from which 5 twins pairs were available in both tissues [26]. Given that these datasets do not include technical replicates, we were forced to implement an extra conservative pre-processing to guarantee reliable results. This meant excluding sites posing strong methylation differences between strands, sites aligning to regions known to yield artefactual high coverage, sites with low or abnormally high coverage, and lastly, sites that were not included in both MZ twins for each pair. Additionally, via computer simulations, we established the vastly conservative threshold of absolute methylation difference of 40% as being very unlikely to have arisen simply from random sampling only (see Additional file 3: Supplementary methods for details). Per MZ twin pair, we then counted sites displaying differences higher and lower than the established threshold within and outside *cPCDHs* to perform enrichment analysis (see Additional file 3: Supplementary methods for details).

**Fig. 5:**
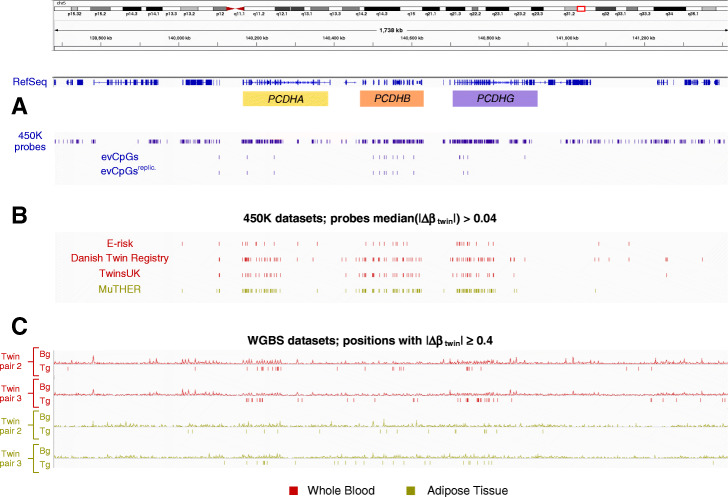
Integration and visualization of the *cPCDH* region (Chromosome 5) using IGV and the MZ twin datasets employed in this study. Tracks are highlighted in red for whole blood and gold for adipose tissue. **a** 450K tracks (dark blue thin bar plots) include the 450K background, total evCpGs and replicated set in adipose tissue. **b** CpGs in this region not included in the evCpG list but showing median(|Δ*β*_twin_|) > 0.04 across 450K twin cohorts are also depicted as red or gold thin bar plots. **c** CpGs with |Δ*β*| ≥ 0.4 (thin bar plots) compared to background (line plots of the normalized density of CpGs) for the two twin pairs displaying the highest WGBS coverage, indicating significant enrichment in MZ twin divergence in the *cPCDH* loci

With regards to the *cPCDH* region, 3 out of 6 twin pairs in blood and 3 out of 7 in adipose tissue were significantly enriched in methylation differences larger or equal to 40% compared to the background (Fig. 4c, Additional file 1: Figures S21-S23); significant twin pairs coincided with those displaying a higher coverage in the *cPCDH* region. For the twin pairs not displaying significant enrichment, this may be due to the 5 to 10 times lower coverage in *cPCDH*s in these samples. Finally, methylation differences were visualized for the two twin pairs posing higher coverage in *cPCDH*s that also displayed significant enrichment in both tissues (Fig. 5). In summary, our discovery in 450K highlighted a strong enrichment for probes subject to stochastic variation distinct from epigenetic drift and measurement error in the *cPCDH* loci. By replicating MZ twin discordance on a different technological platform we have not only gained confidence on our claims concerning *cPCDHs*, but also on the discovery strategy itself.

## Discussion

This study was dedicated to isolate stochastic inter-individual epigenetic variation, distinct from epigenetic drift, genetic influence, and measurement error. To achieve this, we made use of young MZ twins because these are subject to only limited epigenetic drift effects. Additionally, by requiring equivalence between co-twin and inter-individual dissimilarity, we excluded CpGs under genetic control. Given that mechanisms promoting twin-to-twin divergence during embryonic development and early life should potentially generate variation in every individual, we claim to have isolated a universal source of epigenetic inter-individual variation that may individualize even young MZ twins, as it does not rely on epigenetic drift. As previously stated based on different grounds [41, 42], our results confirm that the view of healthy MZ twins posing identical methylomes at a young age is an unrealistic approximation for certain genomic loci.

Under this mindset, we have separated epigenetic drift from epigenetic changes occurring during embryonic development/early life. Since it is unknown whether these two influences operate differently on evCpGs, this segmentation might seem artificial at first. We cannot ignore, however, that a strong link between the definition of epigenetic drift and aging has been established in the literature [8, 43]. Nonetheless, processes occurring during embryonic development and early life are not necessarily related to aging. For example, the agouti mouse is a model for stochastic developmental variation [44]: a long-terminal repeat from an intracisternal-A murine retrotransposon acts as a cryptic promoter for the *agouti* gene, a key regulator of fur color in mice. However, stochastic variable methylation in the cryptic promoter results in variable expression of the *agouti* gene. As a result, genetically identical mice can give rise to a palette of fur colors, ranging from yellow to brown. Though it remains unknown whether evCpG methylation hypervariability operates like an *agouti* cryptic promoter, we believe it is practical to make such distinction, especially given the common misconception that the epigenome of young MZ twins is identical at young ages, but diverges as a result of aging. Given our evidence obtained from children, it is very unlikely that aging alone occurring between childhood and adolescence can explain the observed methylation discordance in the cohort of 18-year-old MZ twins, in agreement with the literature. For example, in [45], they claim that epigenetic changes occurring between birth and 5 years of age outclass those occurring between 5 and 10 years of age.

On another note, from the 4652 variably methylated CpGs tested, only 333 (7%) showed equivalent co-twin and inter-individual variation; hence, 93% of variably methylated CpGs potentially displayed genetic effects. This is in concordance with studies claiming that genetic effects have a strong impact on variably methylated CpGs [1, 2, 5]. Hundreds of identified evCpGs may seem a small number at first glance given the average unshared environment component of 81.0% [6] or 67.4% in the blood [5] and 80.8% in the adipose tissue [24] based on previously published ACE models. However, these estimates are expected to be strongly biased since they include CpGs devoid of inter-individual variation, for which measurement error accounts for most, if not all the observed variation. We emphasize that the aim of this study was not to conduct an exhaustive discovery of all evCpG-like biomarkers in the human methylome but to correctly identify a subset for which we can ensure with high confidence that measurement error is not fully accountable for the observed discrepancies between MZ twins. As evidence for such intention, we excluded a large proportion of CpGs via the applied empirical inter-individual variation threshold. We also employed Bonferroni correction, known to be over-conservative for (epi)genome-wide discoveries; as a result, it is possible and plausible that a proportion of false negatives remains unidentified. Furthermore, our discovery pursues pure inter-individual stochastic variation, hence neglecting ambivalent CpGs posing mixed deterministic genetic and stochastic epigenetic influence, except for minor genetic influences not sufficiently large to escape the equivalence range. Studies evaluating the frequency of CpGs subject to both genetics and environment exist; particularly, those employing genetic and environment interaction (GxE) models [46, 47] claim that most variably methylated CpGs are under the jointed influence of genetics and the environment and that CpGs posing solely environmental influence are extremely rare (only 1 in the entire Infinium MethylationEPIC array [46]); thus, challenging our claims on the hundreds of evCpGs identified in the 450K. However, E in a GxE model unlike in an ACE model is defined as variation explained by a list of environmental phenotypes such as maternal age, smoking, and concentration of certain metabolites. As a result, measurement error, stochastic influence, or the variation associated to variables not included in the model will end up being part of an unexplained variance term. The percentage of unexplained variance for their models was not reported, which could well be larger than the percentage of variance explained. Particularly, stochastic influence is expected to be a key component in evCpGs. Also, GxE models are fitted in cord blood, uninfluenced by the early life period that might contribute to the hypervariability of evCpGs. In summary, it is not possible to extrapolate conclusions from such GxE models to our analysis on evCpGs which takes into account the total variance, without including an unexplained variation term in the model.

That aside, the biggest challenge we faced in this study was separating genuine inter-individual methylation variation from measurement error. Unlike common practices in previously published ACE models [5, 6], we have thoroughly tackled the confounding issue between measurement error and stochastic variation by extending our analysis to both technical and longitudinal replicates. Altogether, we have provided convincing evidence that our observations cannot be explained by measurement error or erratic longitudinal drift. That said, we were unable to cluster the longitudinal replicates of the MZ twin pair of Zhang et al. [19]. Even though we cannot generalize conclusions from a single MZ twin pair, it seems to suggest that short-time variation surpasses co-twin variation at least in this single twin pair. Supporting this idea, the twin pair in question is aged 26 at the time of sample collection; thus, we do not expect strong epigenetic drift contributions. However, we do not know with confidence whether there were differences in the methylation of evCpGs to begin with, as no technical replicates were included at time point zero. Moreover, we cannot ignore that the raw data of the Zhang et al was unavailable; since it is required for our pre-processing approach, the degree of control was smaller than in our core datasets. Moreover, in this analysis, we used only 296 out of the 333 evCpGs; 37 evCpGs (11.1%) were not available. It could well be that by applying our careful pre-processing and normalization that the MZ longitudinal stability incongruence could be eliminated. That aside, our longitudinal analysis on unrelated individuals of evCpGs in whole blood and the replicated set in adipose tissue provide our core evidence on the short-term temporal stability. In summary, future studies are required to shed light on the concern of temporal stability of evCpG methylation in MZ twins, required for any practical application.

Furthermore, throughout the paper, we have made no distinction between embryonic development and early life influences, which may require some explanation. Since evCpGs were discovered in whole blood, the peculiarities in the development of the immunological system apply. Any successful pregnancy requires the correct suppression of any immunological response between the fetus and the mother; thus, both immune systems actively cross-talk to promote a tolerogenic environment [48]. It is well established that newborns deviate from adults in both the innate and the adaptive immune systems [49, 50]. Soon after birth, factors such as the sudden and massive exposure to environmental antigens or the need to overrun mother-embryo allotolerance results in strong post-natal development [51] that potentially affects DNA methylation profiles in blood. There is a vast literature regarding changes in methylation occurring during the first years of life further justifying no need for distinction between development and early life in whole blood DNA methylation data [52–55]. These changes may provide a temporal context to the variation occurring at evCpGs. However, claims concerning this period are often confounded in tissue as whole blood extraction or adipose tissue biopsies tend to be too invasive for pediatric use. Instead, other tissues like buccal epithelial tissue, saliva, blood spots, or cord blood are preferred in practice for children or newborns, with problems of between-tissue variation and lower technical replicability [56–58]. In any case, the lack of co-methylation between post-mortem tissues shown here pushes the balance towards early life experiences, assuming that the post-mortem quality has not altered the quality of the epigenetic profiles. There remains reasonable doubt, however, whether the methylation levels are firstly set during early development and then reset again at latter stages in a tissue-dependent way. Nonetheless, 46% of evCpGs we had identified in whole blood could be successfully replicated in adipose tissue, which supports that the observed changes in blood cannot be caused by imperfect correction of cell composition differences in our pipeline. It also strengthens the confidence that a large portion of our evCpGs are indeed true positive findings. More fundamentally, it suggests the existence of regions concentrated in stochastic epigenetic variation that are common between tissues. We do, however, acknowledge some limitations in our replication, such as a lower sample size, an older age distribution in the TwinsUK cohort, and the lower control on preprocessing given the absence of raw data files.

In addition, our findings highlight the clustered protocadherins as a putative hotspot for stochastic methylation variation in blood and adipose tissue. In the context of aging, strong DNA methylation variation for CpGs within these loci has been previously reported [11, 59–61]. In fact, given the age distribution of the MZ twins in our WGBS validation study, the methylation differences were observed at *cPCDHs* which are expected to be a consequence of not only developmental-early life stochastic variation, but also of epigenetic drift. However, as these loci were highlighted upon the discovery in the E-risk twins cohort composed by 18-year-olds (Fig. 5), it is improbable that only epigenetic drift drives the twin divergence we observe at *cPCDHs*. Functions where *cPCDHs* are expressed in a combinatorial way to generate a wide range of membrane receptors have been previously described in the context of the brain, all of which strictly require gene expression. However, the joint evidence of enrichment in regions with low [G + C], avoidance of important regions for gene regulation, association to genes not expressed in the blood, and enrichment in heterochromatin states all seem to indicate that the majority of the stochastic methylation variation of evCpGs in the blood may be the result of biological noise rather than a biological function. As it stands for *cPCDHs* concretely, it is unlikely that stochastic methylation plays a role in whole blood or adipose tissue given that the mRNA expression in both tissues is residual. More functional studies are required in the future to shed light on the epigenetic dynamics of the *cPCDH* loci in tissues beyond the brain.

Finally, having identified hundreds of CpGs displaying MZ co-twin divergence at young age has implications for future practical applications. For instance, being able to discriminate between human individuals has proven vital in paternity testing, the determination of perpetrators in crime and in the identification of missing persons including victims of mass disasters. Nowadays, genetic markers rich in inter-individual variation are routinely exploited to separate biological samples derived from different human individuals; however, current forensic DNA analysis is unable to discriminate between MZ twins [62]. Extending the concept from genetic to epigenetic fingerprinting by making use of markers such as evCpGs may one day also allow the discrimination of MZ twins, with strong repercussions for law enforcement [63]. Further work is required though to shed light on the feasibility of this approach for any practical forensic application; towards one day being able to provide evidence that is admissible in court, greater understanding is required concerning the measurement error, longitudinal stability in MZ twins, and the statistical modeling of uncertainty. Beyond forensics, we also envision further implications of our findings that branch out into philosophy regarding the uniqueness of human beings.

## Conclusions

We have discovered and characterized hundreds of variably methylated CpGs in the blood of young MZ twins showing equivalent variation among co-twins and unrelated individuals. Being able to cluster technical and longitudinal replicates while distinguishing between young MZ twins, the evCpGs we identified here are enriched in a stochastic variation component distinct from measurement error, genetic influence, and epigenetic drift. Additionally, we have highlighted the clustered protocadherin region in blood and adipose tissue as loci concentrated in MZ co-twin variation and verified our findings across technologies. Future functional studies are required to clarify the underlying molecular mechanisms and putative biological implications of our identified evCpG markers. It has not escaped our notice that such a class of biomarkers may one day allow universal epigenetic fingerprinting, which for instance is relevant in forensics for differentiating MZ twin individuals, typically impossible with standard forensic DNA profiling.

## Methods

### 450K microarray data analysis

All data analysis was performed in R 3.4.4 (“Someone to Lean on”) [64]. We employed the libraries minfi [65], ENmix [66], wateRmelon [67], and missMethyl [68] for reading IDAT files, performing normalization, and quality control. For publicly available data derived from the GEO database, phenotypes were parsed with the help of GEOquery [69]. On this note, we only chose pre-processed data when no similar public data was available in IDAT format. The quality control that can be performed on pre-processed data is inferior, as the information regarding internal 450K control probes (SNP, out-of-band, bisulfite conversion probes, etc.) has been discarded. Also, depending on the choice of authors depositing the dataset, additional information is often unavailable, such as detection *p* value and beads-per-probe matrices, separate intensity channels, CpG-SNPs, or even sex chromosomes, the latter required for checking for sex mismatches. Details concerning the processing of each individual dataset used in this study are available in Additional file 3: Supplementary methods.

### Marker discovery

For the 450K data pertaining the E-risk study, we removed outlier samples, filtered-out potentially noisy probes including low-quality (*n* = 2561), SNP-containing (*n* = 99,337), cross-reactive (*n* = 41,993) [70, 71], and X- (*n* = 11,232) and Y-chromosomal (*n* = 416) probes. We normalized in parallel using three popular methods: stratified quantile normalization [65], dasen [67], and oob_RELIC_QN_BMIQ [66]. Additionally, we used ComBat [72] and a modified Houseman method [73, 74] to correct for potential batch effects and for whole-blood cell composition differences, respectively (Additional file 1: Figure S5). Following normalization, probes displaying either ICC < 0.37 [27] or IQR < 0.07 were filtered out. On the one hand, ICC measures the proportion of non-technical variance compared to the total variance. An ICC of zero indicates that 100% of the variance could be explained by variance between technical replicates (a.k.a. measurement error). In the 450K array, probes displaying ICC close to zero are common and mostly represent probes lacking any inter-individual variation. On the other hand, an IQR of 0.07 is expected from measurement error only in a CpG following a beta distribution with mean = 0.5 and standard deviatio*n* = 0.05.

For the remaining 4652 CpGs, we computed per CpG the absolute difference in methylation for twin pairs and for all combinations of unrelated pairs. We tested for similarity under the paradigm of equivalence testing with a two one-sided tests (TOST) procedure based on the Yuen *t* test that tolerates non-normality [75]. We subsequently selected significant CpGs (*α*/*n* = 0.05/4652; Bonferroni-corrected) and intersected significant hits across normalizations. Employing several normalization methods is not a standard routine in epigenome-wide studies and was initially introduced as another quality-control step. But given that strong differences between methods were observed (Additional file 1: Figures S3-4, S6), and to avoid normalization method-specific outcomes, we decided to search for significant results across multiple normalization strategies. The parameter epsilon (*ε*), which characterizes the resolution at which the difference in two means can be defined as equivalent, was also established per normalization; we justify this choice as the |Δ*β*| distribution in twin/unrelated pairs highly differed between normalizations. We based the selection of epsilon solely on the distribution of the trimmed mean of |Δ*β*|^twin^ across all tested CpGs. Discovery statistics and effect sizes were visualized via Manhattan and concordance plot, respectively (see Additional file 3: Supplementary methods for details).

### Evaluation of measurement error and longitudinal stability

To ensure that technical measurement on its own cannot explain the results obtained in the discovery, we confirmed similarity in distribution of number of beads, detection *p* value, and ICC between significant (*n* = 333) and non-significant CpGs (*n* = 4319). Secondly, the set of evCpGs was evaluated in the resolution of technical replicates within MZ twin pairs and in the pairing of longitudinal replicates by employing heatmap and unsupervised hierarchical clustering. We compared their performance with a set of negative control CpGs previously reported for strong genetic effects, which were not expected to resolve between MZ twins or longitudinal replicates. These derived via ranking reported blood mQTL CpGs by significance in adolescents from the ARIES cohort [7] and selecting a number equal to that of available evCpGs in the given cohort.

### Assessment of aging effects

For epigenetic clock, we evaluated the association with age via linear regression, where the dependent variable is the evCpG methylation value and the independent variable is age, correcting for sex as a covariate. For epigenetic drift, we evaluated heteroscedasticity (increased variance with age) via the White test. We preferred this option to an ordinary Breusch-Pagan test as in [11], as it additionally includes a quadratic term for age in the auxiliary linear model.

### Replication across tissues

To assess whether evCpG methylation is subject to tissue-to-tissue variation, we made use of a large panel of post-mortem tissues to achieve a high number of tissues per individual; more details are available in Additional file 3: Supplementary methods. We performed multi-dimensional scaling (MDS) for the 65 450K SNP probes, the genetically controlled CpGs employed previously and for evCpGs. Additionally, we performed replication of the discovery in adipose tissue on *dataset-I* similarly to evCpG discovery in whole blood, but in absence of cell composition and batch effect correction. Finally, we tested time stability of replicated evCpGs on *dataset-J* for which temporal ICC’s were estimated.

### Functional annotation

We deeply annotated evCpGs based on the IlluminaHumanMethylation450kanno.ilmn12.hg19 file (Additional file 2). Furthermore, we extracted 500 bp up- and down-stream evCpGs and background via samtools (v1.9) [76]. We ran Homer (v4.10) [77] in search for known and de novo motif enrichment analysis. We input fasta files into R and computed [G + C] content with the help of the seqinr R-package [78]. Also, as part of Roadmap Epigenomics mapping consortium [32], a hidden Markov model had been built based on data derived from PBMC from peripheral blood, by which the whole genome was segmented into 15 categories or states, ChromHMM. The data was obtained from the Encyclopedia of DNA Elements (ENCODE) (accession ID: ENCSR550VPH) in bigBed format, which was subsequently converted to a Bed format file with the BigBedToBed tool obtained from the UCSC server (http://hgdownload.soe.ucsc.edu/admin/exe/). We subsequently annotated all probes in the 450K with its respective category and performed enrichment for evCpGs. On the same note, median transcriptional expression levels for 247 out of the 264 evCpG-associated genes were extracted from the GTEx portal. Also, known and predicted imprinted human genes were extracted from the Geneimprint database (http://www.geneimprint.com/site/genes-by-species), human metastable epiallele CpGs were extracted from [33], and EWAS-associated trait CpG annotation was obtained from the EWAS Atlas [3], while mQTLs discovered in the blood of adolescents were obtained from [7]. Gene Ontology (GO) term enrichment was performed with the library missMethyl [68] that can correct for the number of probes per gene.

### WGBS pre-processing

Unfiltered processed whole-genome bisulfite sequencing (WGBS) data derived from whole blood belonging to MZ twins were obtained from the ArrayExpress database (accession ID: E-MTAB-3549). Similarly to [26], we excluded sites with more than 20% methylation differences between the strands or sites that fell within the Duke Excluded Regions (https://www.encodeproject.org/annotations/ENCSR797MUY/) or the DAC Blacklisted Regions (https://www.encodeproject.org/annotations/ENCSR636HFF/), known to yield artefactual high coverage. We additionally applied both high- and low-end coverage filters. We excluded (i) sites with coverage less or equal to 10 reads and (ii) larger than the per-sample 99.9% quantile. Altogether, this procedure improves the accuracy of the methylation estimates per site and filters out possible PCR artifacts at the high end of the coverage. Per twin pair, we then selected only those sites that were common.

Via simulations, we estimated the 95% quantile of the sampling |*∆β*| distribution to be 0.4 for 10 reads given no *β* difference between samples (see Additional file 3: Supplementary methods for details). Differences higher or equal to this threshold are very unlikely to have arisen from random sampling only. Finally, positional enrichment analysis was performed on the *cPCDH* region (chr5:140165876:140892546 for genome assembly hg19). Per twin, we computed the number of sites with |Δ*β*|^twin^ ≥ 0.4 and |Δ*β*|^twin^ < 0.4 within and outside this region and performed a Fisher’s exact test to obtain an enrichment *p* value.

## Supplementary Information

**Additional file 1.** Supplementary figures.

**Additional file 2.** Supplementary file.

**Additional file 3.** Supplementary methods.

**Additional file 4.** Review history.

## Data Availability

All datasets employed in this study are compiled on Table 1. Additional details concerning cohort characteristics can be found in Additional file 3: Supplementary methods. The accession identifiers are also listed here: (*dataset-A*) GSE105018 (GEO) [79], (*dataset-B*) GSE61496 (GEO) [80], (*dataset-C*) GSE51388 (GEO) [81], (*dataset-D*) GSE104812 (GEO) [82], (*dataset-E*) GSE87571 (GEO) [83], (*dataset-F*) partially available at GSE121633 and at GSE62992 (GEO) [84, 85], (*dataset-G*) GSE99863 (GEO) [86], (*dataset-H*) GSE50192 (GEO) [87], (*dataset-I*) E-MTAB-1866 (ArrayExpress) [88], (*dataset-J*) GSE103768 (GEO) [89], and (*dataset-K*) E-MTAB-3549 (ArrayExpress) [90]. The complete TwinsUK methylation dataset can be applied for through the TwinsUK data access procedures described in detail at (https://twinsuk.ac.uk/resources-for-researchers/access-our-data/). Data analysis was performed by employing custom R-scripts, which have been released to the public domain under an MIT license at GitHub [91] and at the Zenodo digital object identifier-assigning repository (10.5281/zenodo.4271916) [92].

## Abbreviations

450K: Infinium HumanMethylation450K Beadchip
5’-UTR: 5’-untranslated region
A component: additive genetic component
C component: common environmental component
*cPCDH*: clustered protocadherins
CpG: cytosine-guanine dinucleotide
DZ: dizygotic
E component: unshared environmental component
ENCODE: encyclopedia of DNA elements
evCpG: equivalently variable CpG between co-twins and unrelated individuals
EWAS: epigenome-wide association study
GO: gene ontology
GTEx: the genotype-expression project
ICC: intra-class correlation coefficient
IDAT: intensity data file
ICC: intra-class correlation coefficient
IQR: inter-quantile range
MZ: monozygotic
IQR: inter-quantile range
MDS: multidimensional scaling
mQTL: methylation quantitative trait locus
PBMC: peripheral blood mononuclear cell
PCR: polymerase chain reaction
SNP: single nucleotide polymorphism
STR: short tandem repeat
TOST: two one-sided tests
TSS: transcription starting site
WGBS: whole genome bisulfite sequencing
