# Supplementary material for: Equivalent DNA methylation variation between monozygotic co-twins and unrelated individuals reveals universal epigenetic inter-individual dissimilarity
Source: Genome Biol. 2021 Jan 5;22:18. doi: 10.1186/s13059-020-02223-9 (PMC7786996; doi:10.1186/s13059-020-02223-9)
Supplement: Supplementary file 1 — Additional file 1. Supplementary figures. [file 13059_2020_2223_MOESM1_ESM.pdf]

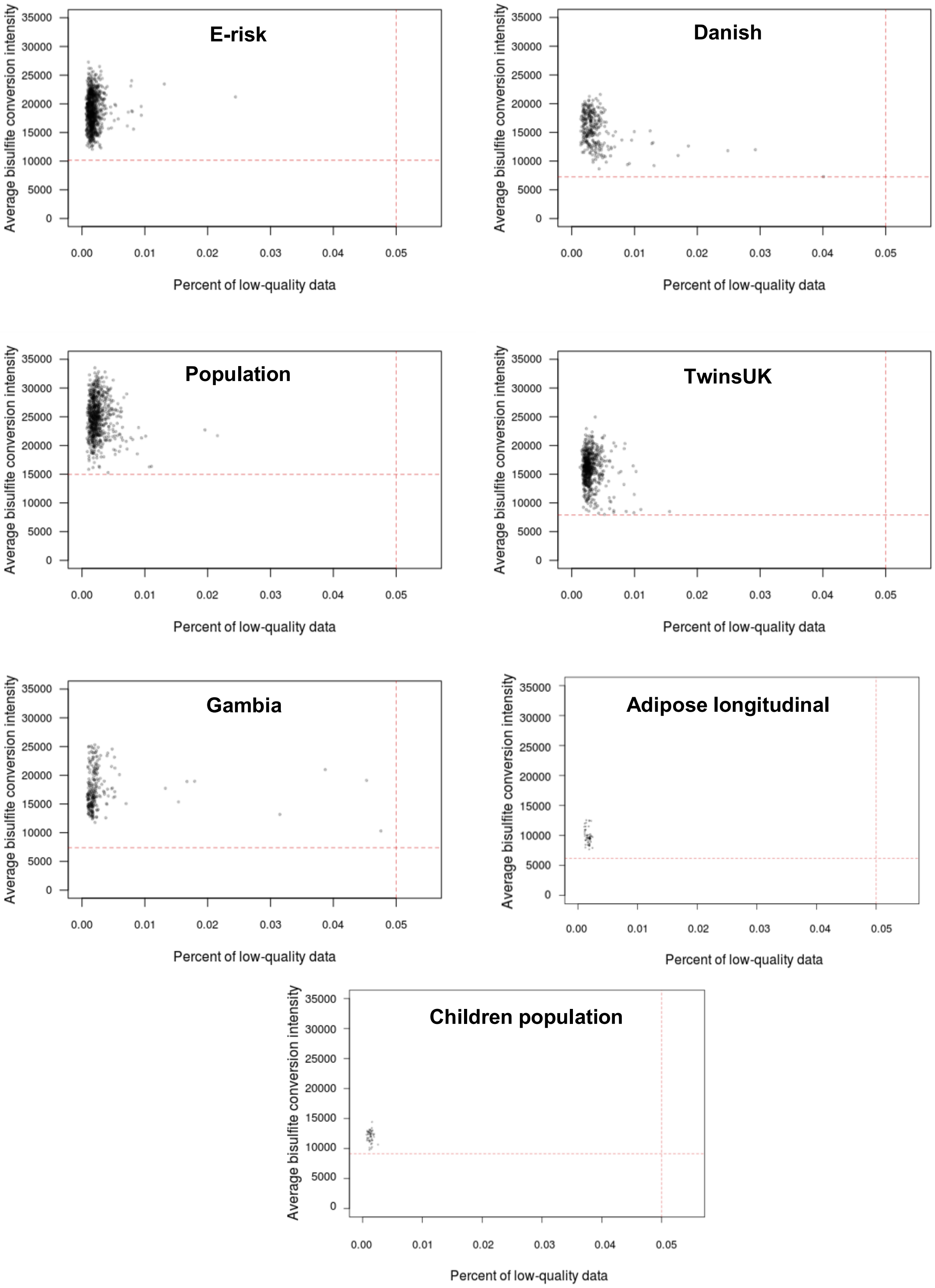

Fig S1. Sample quality control across 450K datasets after exclusion of low-quality samples. Under default parameters ENmix::Qcinfo flags any sample with percent of low-quality data higher than 0.05 and average bisulfite conversion intensity of mean - 3 × standard deviations. The correspondance between the names employed and the datasets is the following: E-risk (*dataset-A*), Danish (*dataset-B*), population (*dataset-E*), TwinsUK (*dataset-F*), Gambia (*dataset-G*), adipose longitudinal (*dataset-J*) and children population (*dataset-D*).

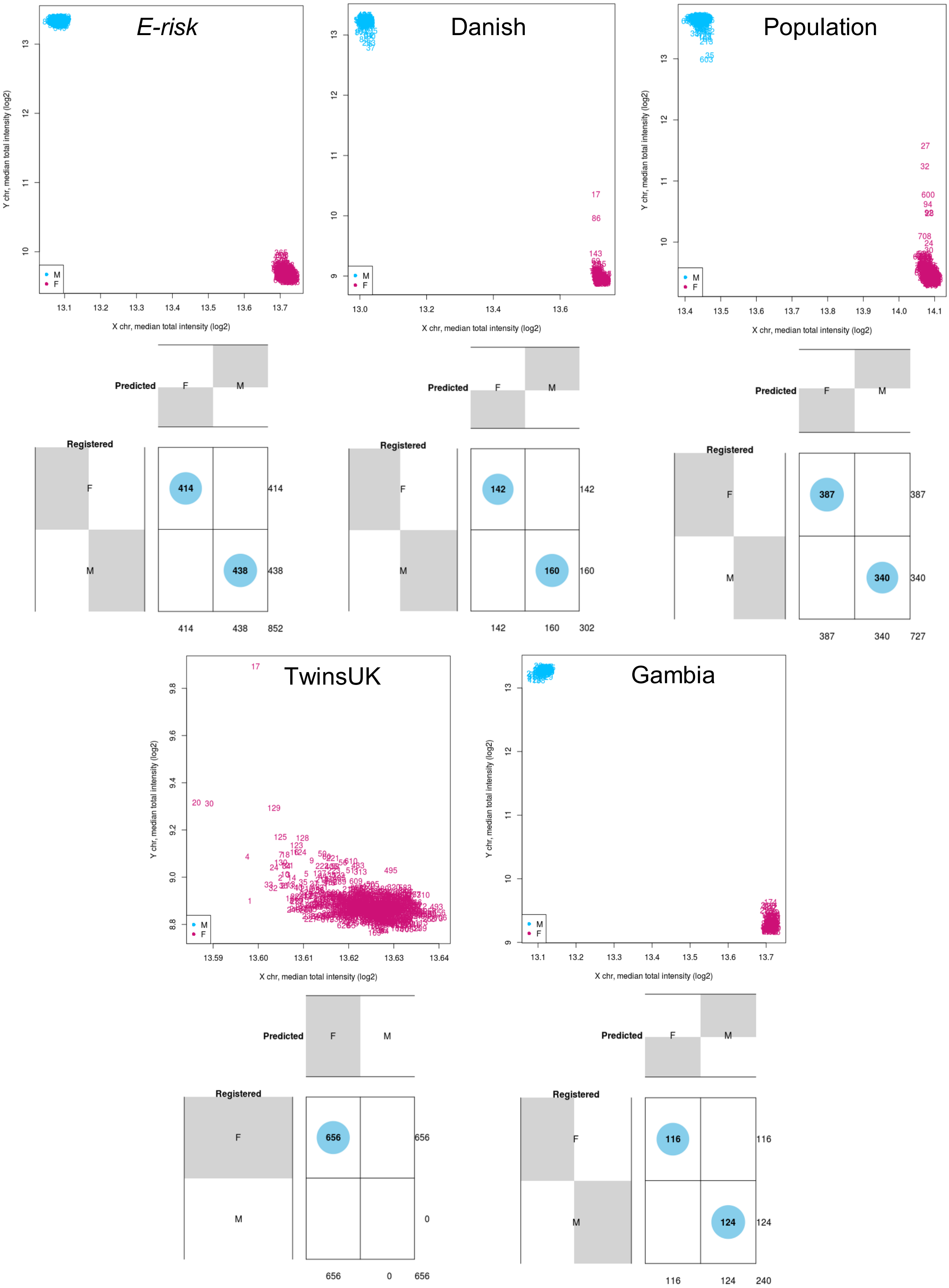

Fig S2A. Sex quality control across 450K datasets after exclusion of samples with wrongly assigned sex. The correspondance between the names employed and the datasets is the following: E-risk (*dataset-A*), Danish (*dataset-B*), population (*dataset-E*), TwinsUK (*dataset-F*) and Gambia (*dataset-G*).

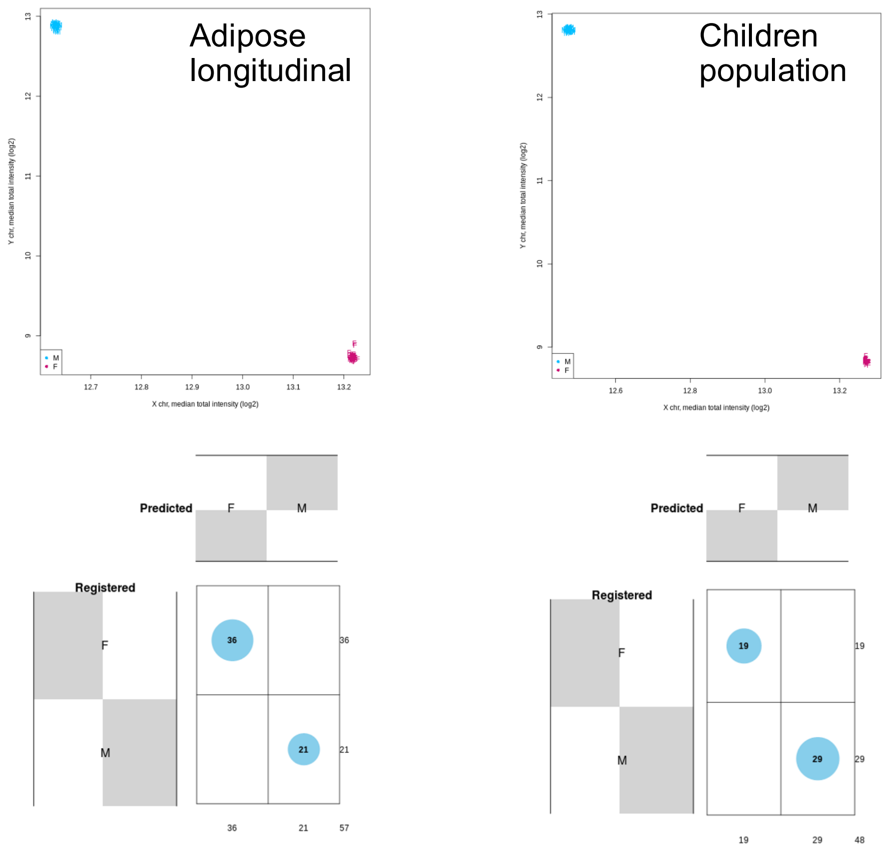

Fig S2B. Sex quality control across 450K datasets after exclusion of samples with wrongly assigned sex. The correspondance between the names employed and the datasets is the following: adipose longitudinal (*dataset-J*) and children population (*dataset-D*).

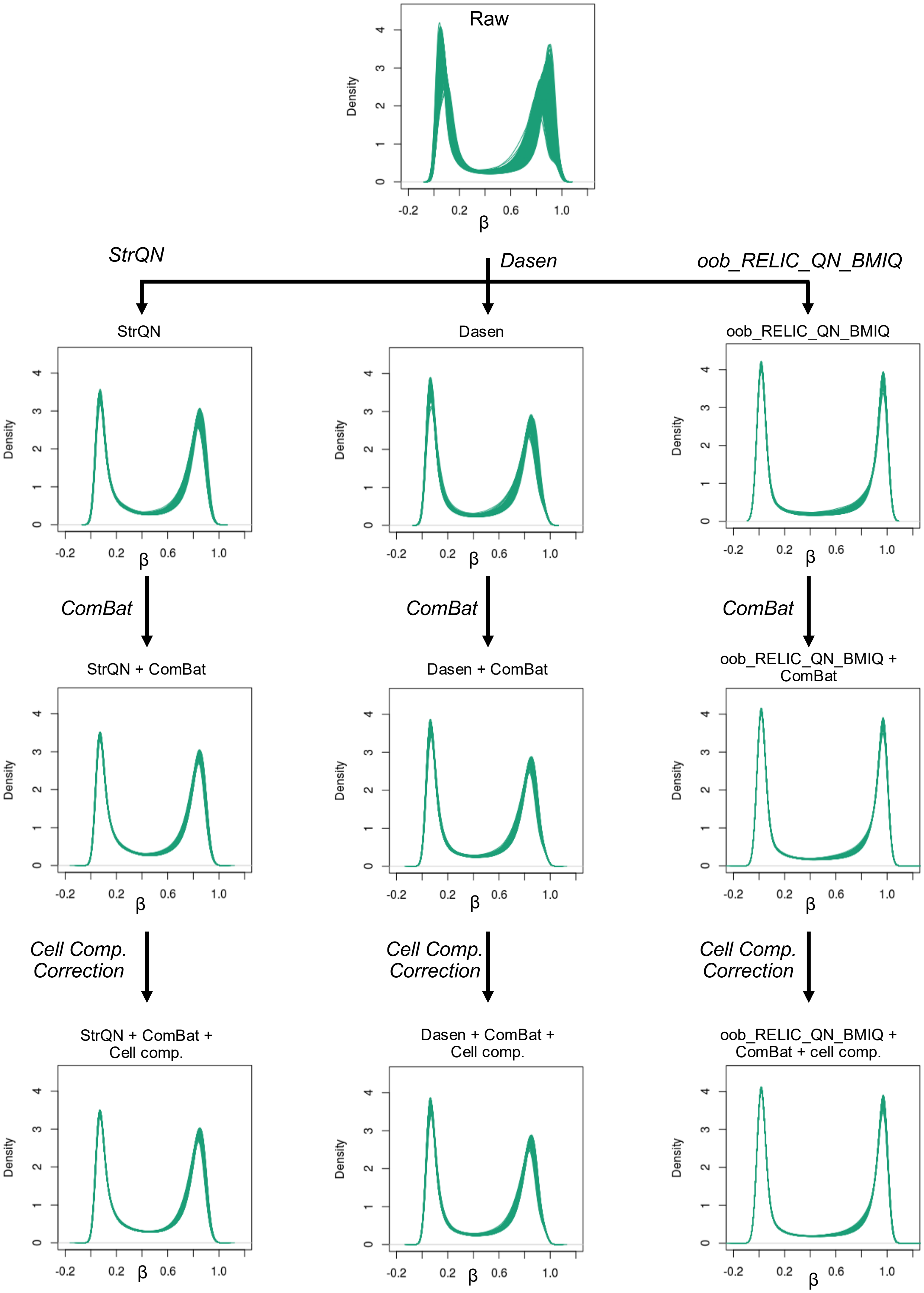

Fig S3. Beta-value distribution across all non-filtered CpGs at each stage of the pipeline for three different normalization methods on the discovery cohort.

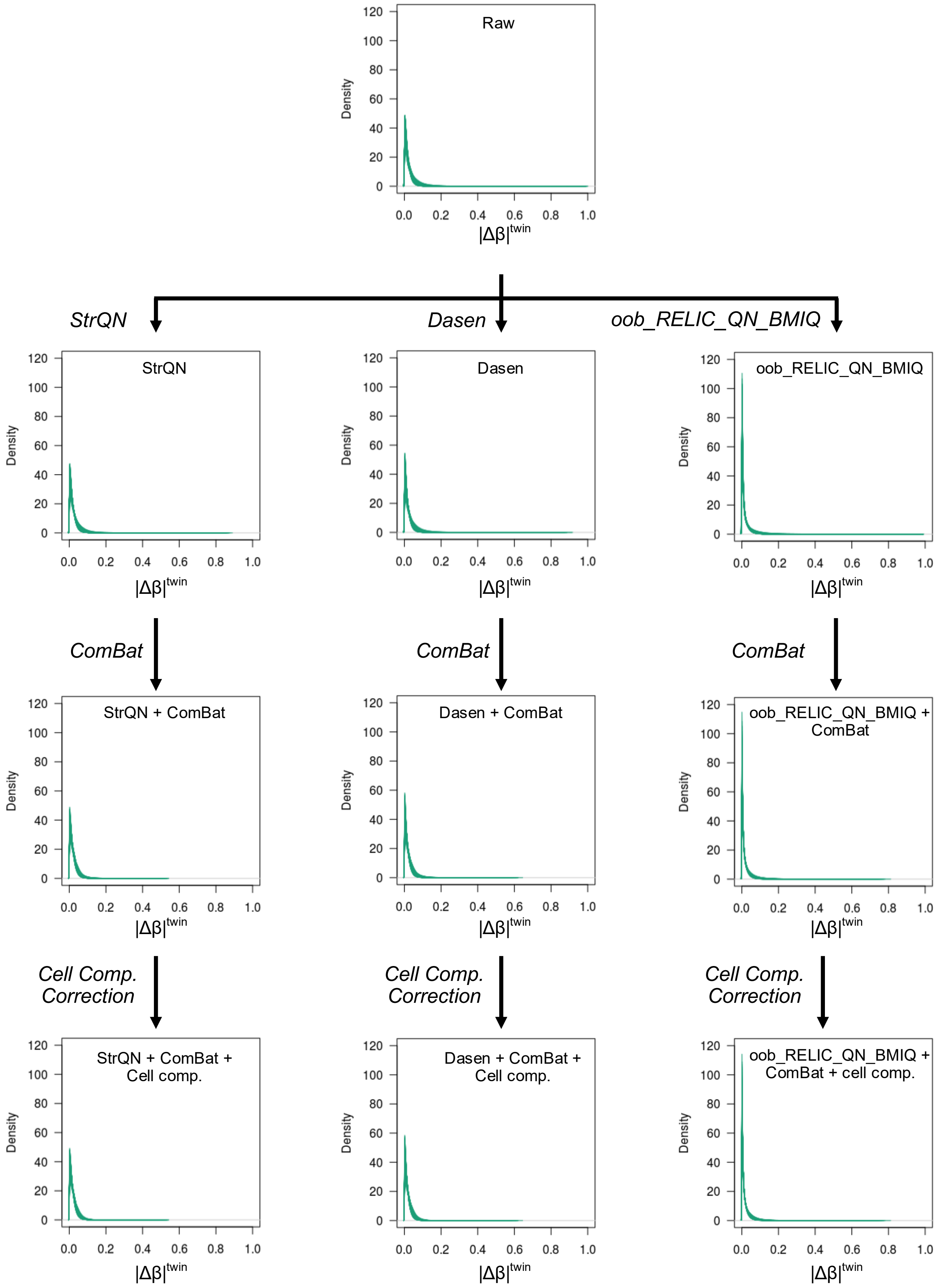

Fig S4. Absolute differences of beta-value distribution between MZ twin pairs across all non-filtered CpGs at each stage of the pipeline for three different normalization methods on the discovery cohort.

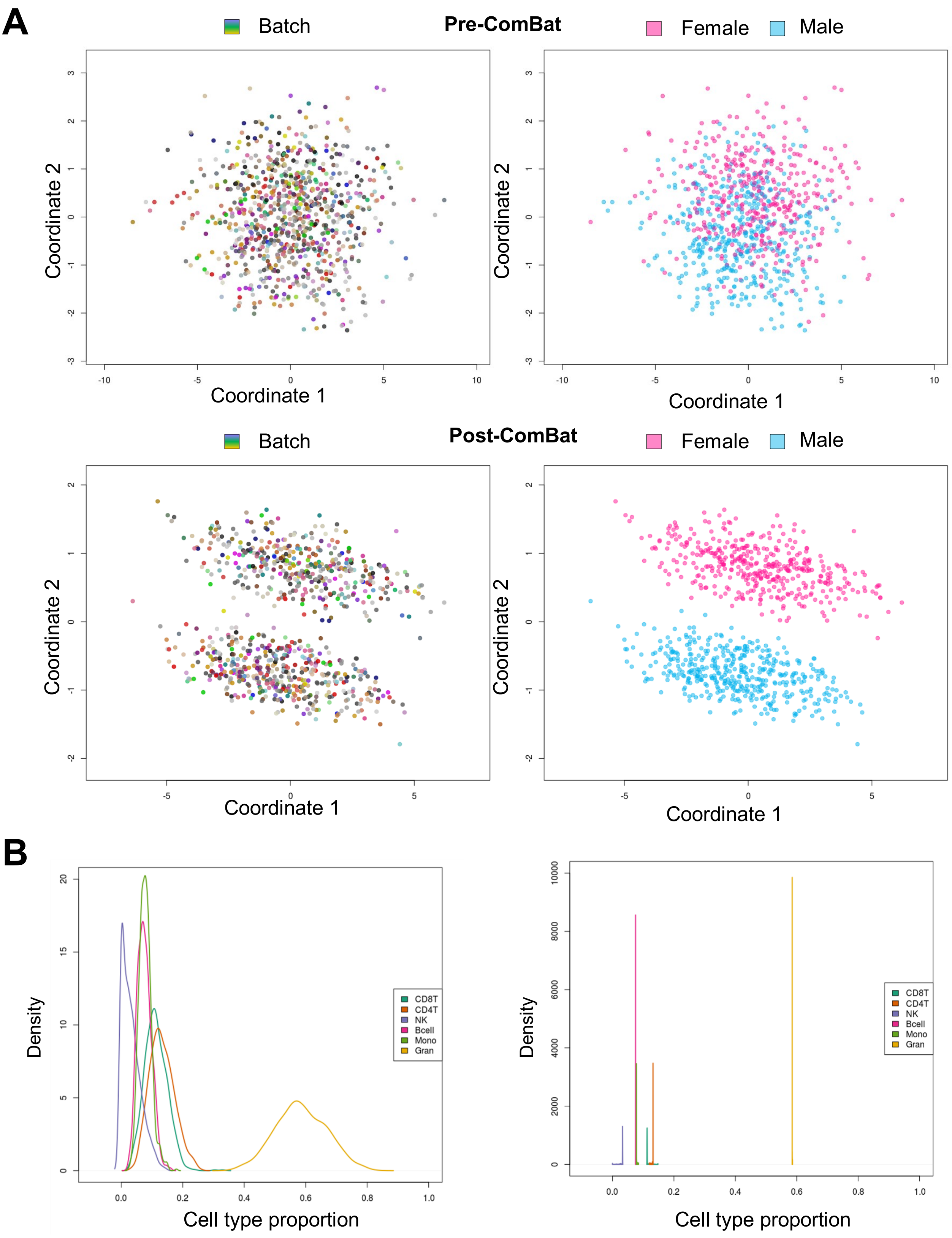

Fig S5. Correction of batch effects and cell composition differences in the discovery cohort. **a**. MDS plot on the 5,000 most variably methylated CpGs for StrQN and StrQN+ComBat, coloured by batch (left) or by sex (right). **b**. Predicted cell counts density distribution across individuals on StrQN+comBat with (right) and without (left) cell composition correction.

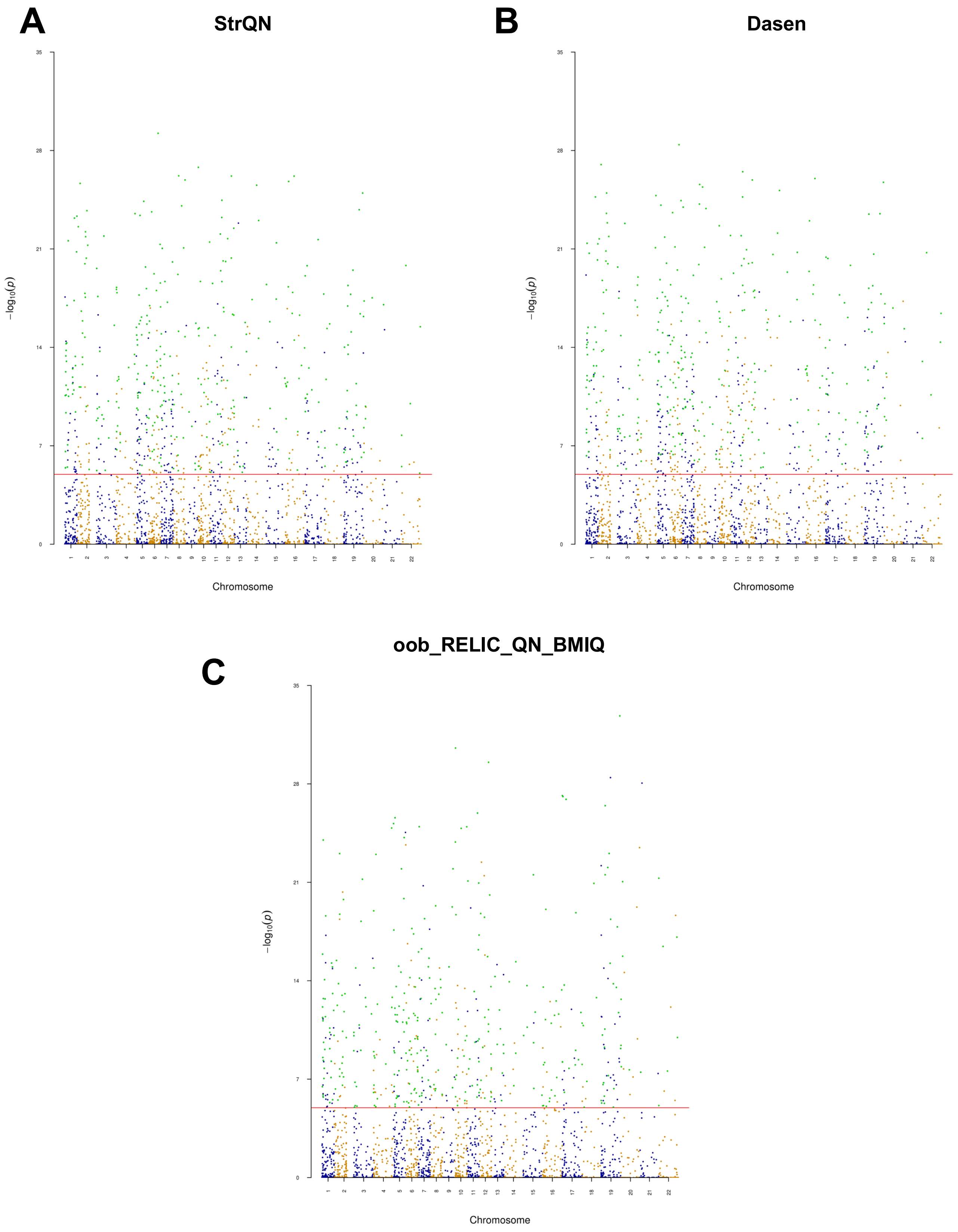

Fig S6. Individual Manhattan plots for each normalization method. Significance is displayed across chromosomes for all CpGs tested for equivalence (odds and even chromosomes represented either in blue or orange). Employed normalization methods include: **a**. StrQN. **b**. Dasen. **c**. oob_RELIC_QN_BMIQ. evCpGs are highlighted in green.

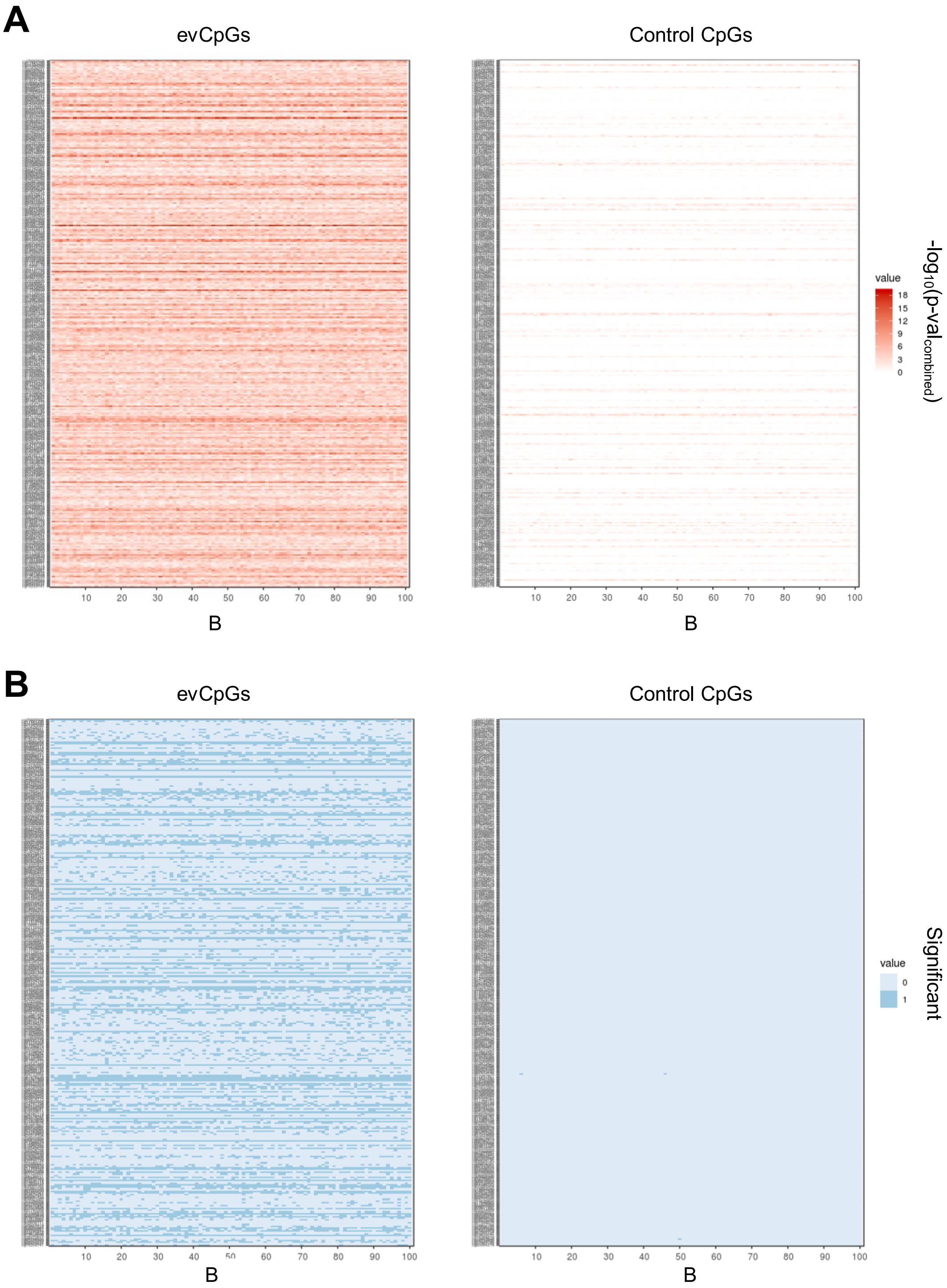

Fig S7. Addtional verification on the evCpG discovery. **a**. Log_{10}-transformed p-values heatmap (combined across normalisations with max_i) over B = 100 unrelated matchings for the total 333 evCpGs and 333 randomly selected non-significant CpGs (control CpGs). **b**. Binary heatmap indicating whether a given p-value was significant based on the significance threshold employed at the discovery stage (α = 0.05/4,652).

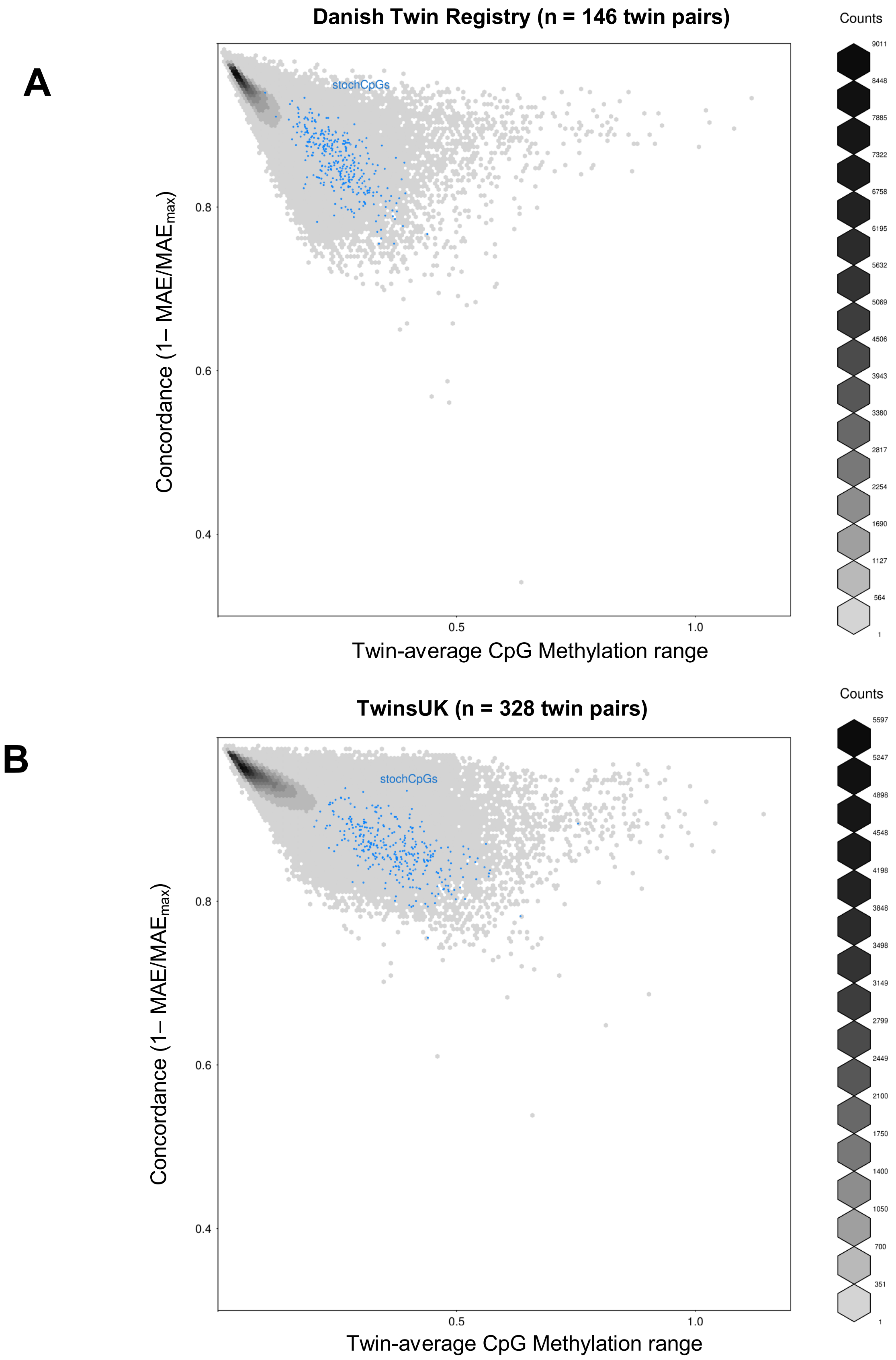

Fig S8. Agreement between MZ twins measured as concordance plotted against methylation range (See Supplementary methods for details) for **a**. Danish Twin Registry and **b**. TwinsUK. evCpGs are highlighted in blue.

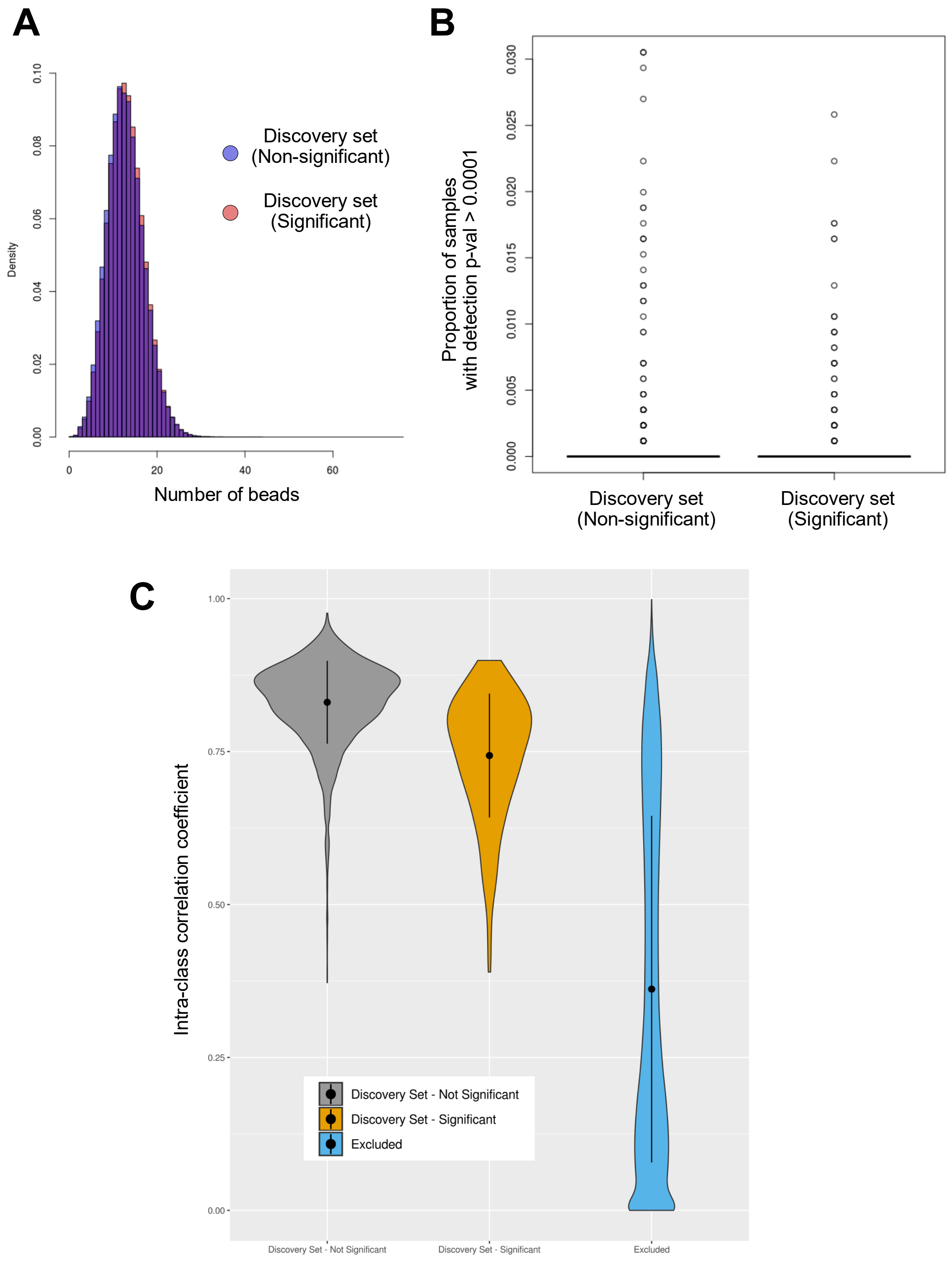

Fig S9. Additional technical control of evCpGs in the E-risk discovery cohort (*dataset-A*). **a**. Distribution of number of beads among significant (evCpGs) and non-significant hits. **b**. Proportion of low-quality samples per CpG among significant (evCpGs) and non-significant hits. **c**. Intra-class correlation coefficient distribution (extracted from the ARIC study) among significant (evCpGs), non-significant hits and excluded probes.

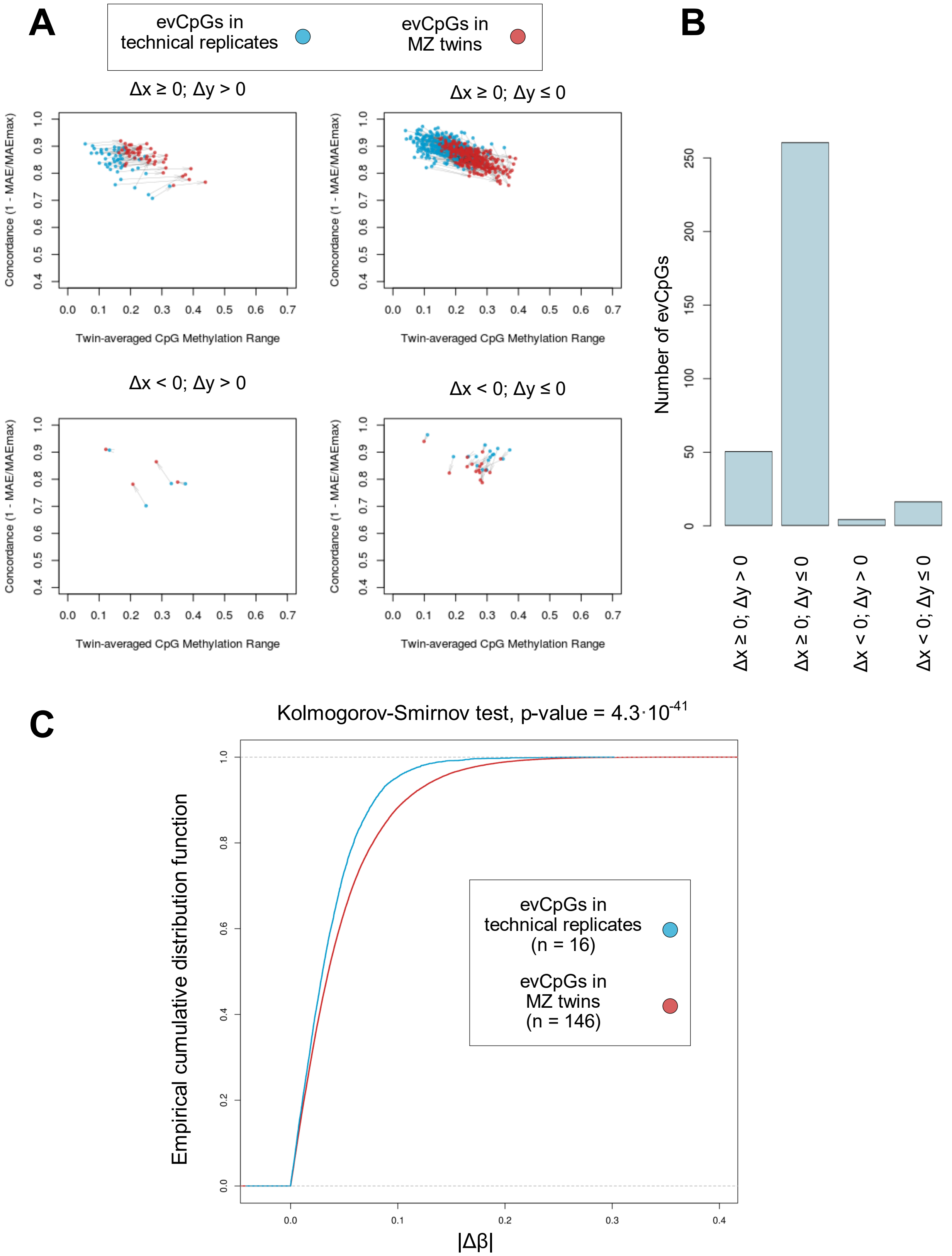

Fig S10. Superior evCpG variation between MZ twins compared to technical replicates in the Danish Twin Registry. **a**. Concordance against methylation range for MZ twin pairs (red) and technical replicates (blue) for 329/333 evCpGs showing high-quality. Arrows link technical replicate to twin pair nodes. In case that the observed variation is beyond technical noise, it is expected that the methylation range will increase (Δx = Δrange > 0), while the concordance will decrease (Δy = Δconcordance < 0). **b**. Number of evCpGs per category mentioned in (a). **c**. Empirical cumulative distribution function of |Δβ| in evCpGs in MZ twins and technical replicates.

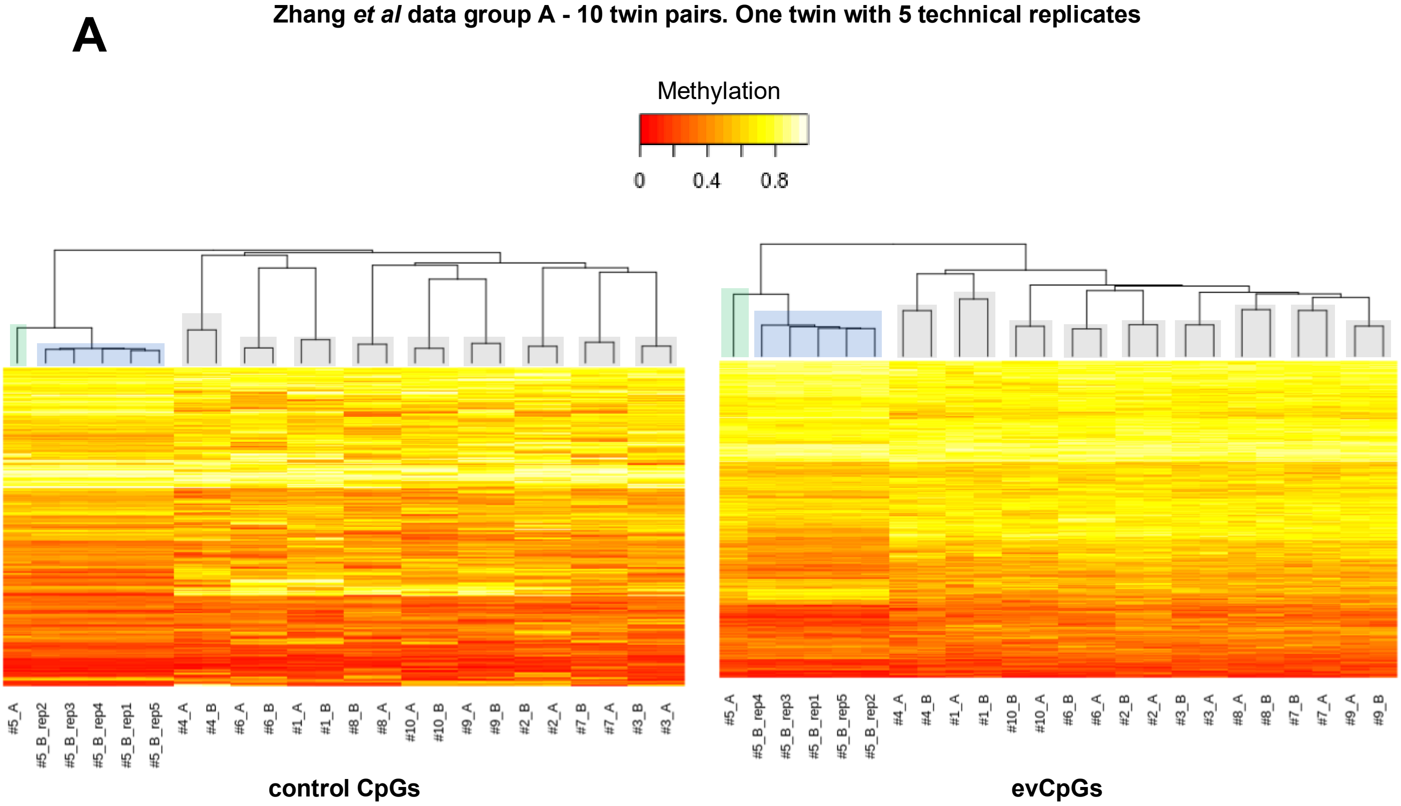

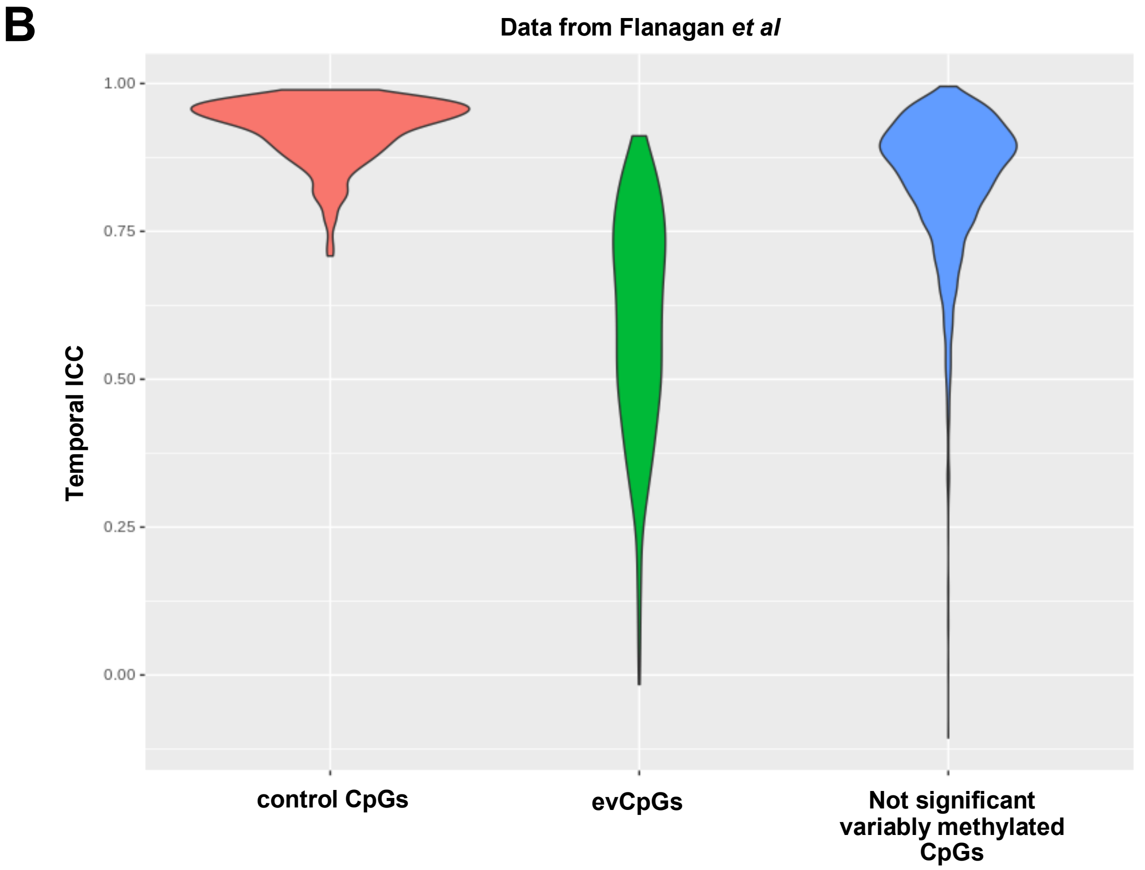

Fig S11. a. Heatmap with unsupervised hierarchical clustering based on 299 out of 333 evCpGs and equal number of control probes in group A from Zhang *et al.* **b**. Flanagan temporal ICC distribution of the set of control CpGs, evCpGs and non-significant hits out of the variably methylated CpGs.

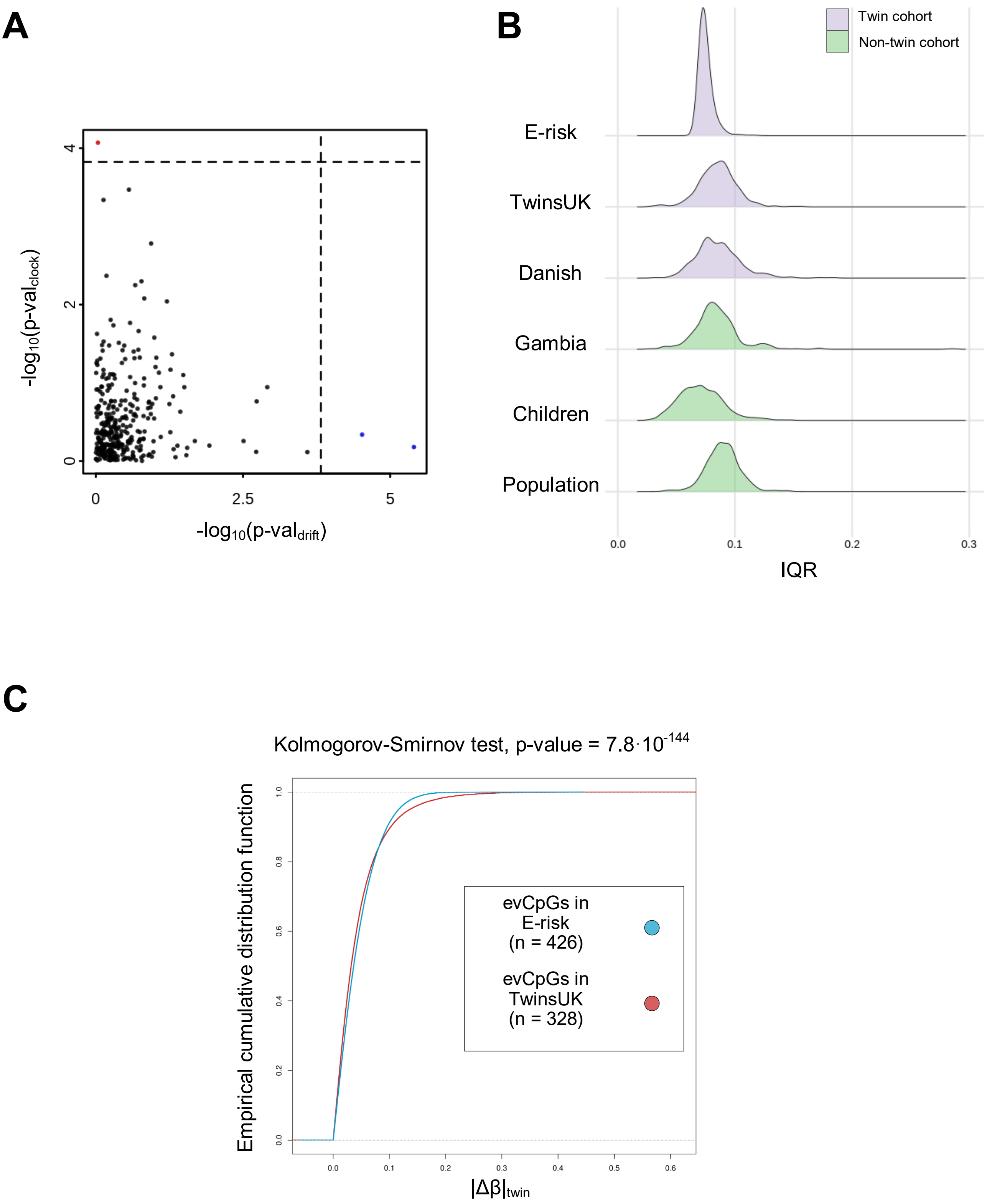

Fig S12. Additional results on the relation between evCpGs and aging. **a**. Identified infancy-to-adolescence epigenetic drift changes. **b**. Inter-quantile range (IQR) distribution across cohorts. **c**. Empirical cumulative distribution of the absolute differences in evCpG methylation in monozygotic twins belonging to E-risk and TwinsUK.

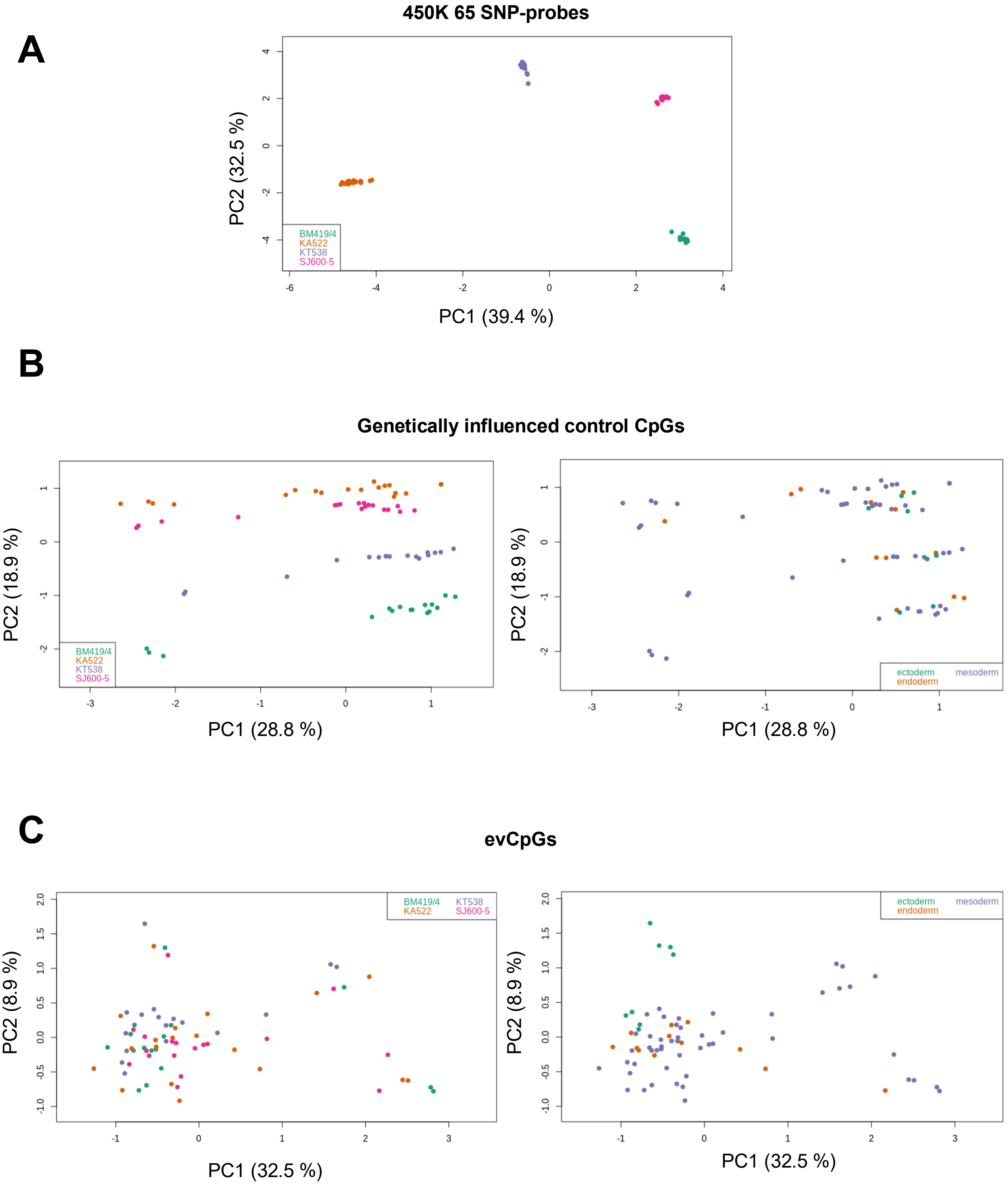

Fig S13. Post-mortem inter-tissue evCpG variation. MDS plots were performed on **a**. 65 SNPs in the 450K, **b**. Genetically influenced control CpGs and **c**. evCpGs. Colours highlight which individual corpse (left) or embryonic layer (right) the sample derives from.

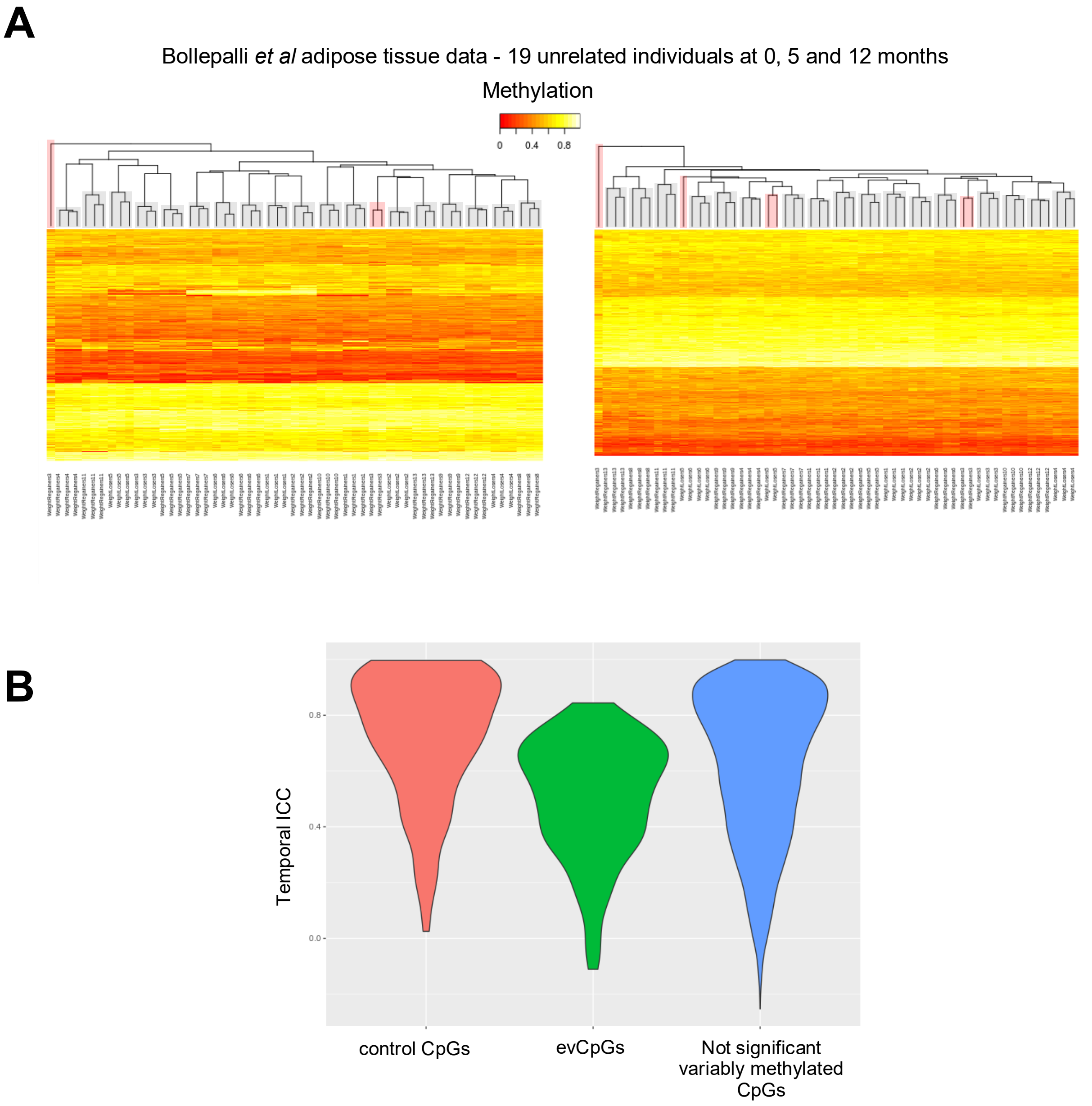

Fig S14. Longitudinal stability in adipose tissue **a**. Heatmap with unsupervised hierarchical clustering based on evCpGs and a set of genetically influenced control CpGs derived from previously reported mQTLs in blood **b.** Distribution of temporal ICC excluding one outlier in both evCpGs and control CpGs.

Homer *de novo* Motif Results (./)

Known Motif Enrichment Results
Gene Ontology Enrichment Results
If Homer is having trouble matching a motif to a known motif, try copy/pasting the matrix file into STAMP
More information on motif finding results: HOMER | Description of Results | Tips
Total target sequences = 333
Total background sequences = 343165
* - possible false positive

Rank	Motif	P-value	log P-pvalue	% of Targets	% of Background	STD(Bg STD)	Best Match/Details	Motif File
1 *	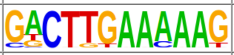	1e-11	-2.729e+01	4.20%	0.28%	290.1bp (343.1bp)	At2g41835(C2H2)/col-At2g41835-DAP-Seq(GSE60143) /Homer(0.760) More Information | Similar Motifs Found	motif file (matrix)
2 *	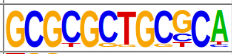	1e-11	-2.614e+01	3.90%	0.24%	272.2bp (261.7bp)	ZC3H10(Znf)/Homo_sapiens-RNCMPT00085-PBM/HughesRNA(0.713) More Information | Similar Motifs Found	motif file (matrix)
3 *	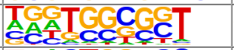	1e-10	-2.391e+01	23.12%	10.59%	282.8bp (275.5bp)	brk/dmmpmm(Bergman)/fly(0.801) More Information | Similar Motifs Found	motif file (matrix)
4 *	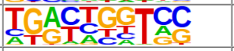	1e-9	-2.279e+01	8.71%	2.05%	262.9bp (284.0bp)	PH0140.1_Pknox1/Jaspar(0.681) More Information | Similar Motifs Found	motif file (matrix)
5 *	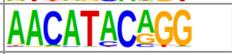	1e-9	-2.278e+01	7.81%	1.65%	281.9bp (326.2bp)	HNRPLL(RRM)/Homo_sapiens-RNCMPT00178-PBM/HughesRNA(0.691) More Information | Similar Motifs Found	motif file (matrix)
6 *	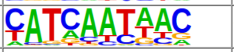	1e-9	-2.273e+01	9.61%	2.49%	315.3bp (347.1bp)	PH0134.1_Pbx1/Jaspar(0.773) More Information | Similar Motifs Found	motif file (matrix)
7 *	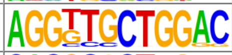	1e-9	-2.265e+01	3.00%	0.15%	253.4bp (267.6bp)	SAMD4A(SAM)/Homo_sapiens-RNCMPT00063-PBM/HughesRNA(0.716) More Information | Similar Motifs Found	motif file (matrix)
8 *	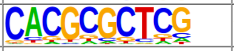	1e-9	-2.128e+01	30.03%	16.45%	273.1bp (265.5bp)	FHY3/MA0557.1/Jaspar(0.901) More Information | Similar Motifs Found	motif file (matrix)
9 *	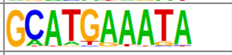	1e-9	-2.102e+01	9.61%	2.67%	297.8bp (342.6bp)	ASD-1(RRM)/Caenorhabditis_elegans-RNCMPT00180-PBM/HughesRNA(0.724) More Information | Similar Motifs Found	motif file (matrix)
10 *	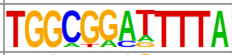	1e-9	-2.091e+01	2.40%	0.09%	229.5bp (308.1bp)	ERF105(AP2EREBP)/colamp-ERF105-DAP-Seq(GSE60143) /Homer(0.640) More Information | Similar Motifs Found	motif file (matrix)
11 *	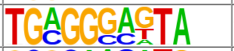	1e-8	-1.976e+01	5.41%	0.90%	307.4bp (315.8bp)	Ptx1/dmmpmm(Noyes_hd)/fly(0.670) More Information | Similar Motifs Found	motif file (matrix)
12 *	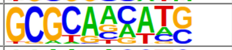	1e-8	-1.967e+01	21.62%	10.52%	257.6bp (275.1bp)	DAL82/MA0291.1/Jaspar(0.809) More Information | Similar Motifs Found	motif file (matrix)
13 *	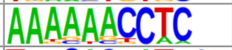	1e-8	-1.850e+01	12.01%	4.34%	245.2bp (340.6bp)	TIA1(RRM)/Homo_sapiens-RNCMPT00165-PBM/HughesRNA(0.752) More Information | Similar Motifs Found	motif file (matrix)
14 *	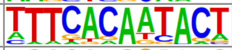	1e-7	-1.813e+01	6.01%	1.25%	230.2bp (363.3bp)	slbo/dmmpmm(Bergman)/fly(0.726) More Information | Similar Motifs Found	motif file (matrix)
15 *	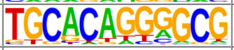	1e-7	-1.791e+01	9.61%	3.05%	263.8bp (265.0bp)	NRF1/MA0506.1/Jaspar(0.702) More Information | Similar Motifs Found	motif file (matrix)
16 *	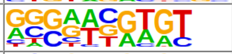	1e-6	-1.588e+01	4.80%	0.92%	267.9bp (282.7bp)	HNRNPL(RRM)/Homo_sapiens-RNCMPT00091-PBM/HughesRNA(0.670) More Information | Similar Motifs Found	motif file (matrix)
17 *	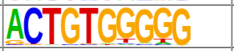	1e-6	-1.534e+01	9.91%	3.60%	326.2bp (286.1bp)	MET28(MacIsaac)/Yeast(0.789) More Information | Similar Motifs Found	motif file (matrix)
18 *	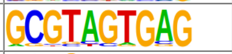	1e-6	-1.520e+01	6.01%	1.50%	258.9bp (274.0bp)	At5g04390(C2H2)/col200-At5g04390-DAP-Seq(GSE60143) /Homer(0.677) More Information | Similar Motifs Found	motif file (matrix)
19 *	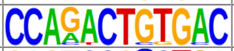	1e-6	-1.494e+01	2.10%	0.13%	241.2bp (300.3bp)	MET31/Literature(Harbison)/Yeast(0.652) More Information | Similar Motifs Found	motif file (matrix)
20 *	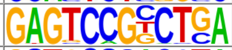	1e-6	-1.453e+01	2.10%	0.13%	323.2bp (293.7bp)	BAS1/BAS1_SM/2-BAS1(Harbison)/Yeast(0.602) More Information | Similar Motifs Found	motif file (matrix)
21 *	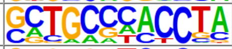	1e-6	-1.416e+01	9.31%	3.43%	270.6bp (274.6bp)	PB0029.1_Hic1_1/Jaspar(0.743) More Information | Similar Motifs Found	motif file (matrix)
22 *	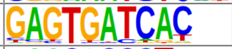	1e-6	-1.388e+01	7.51%	2.43%	218.9bp (308.2bp)	eyg/dmmpmm(Bergman)/fly(0.718) More Information | Similar Motifs Found	motif file (matrix)
23 *	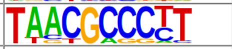	1e-5	-1.321e+01	5.71%	1.56%	140.7bp (288.4bp)	hkb/dmmpmm(Papatsenko)/fly(0.823) More Information | Similar Motifs Found	motif file (matrix)
24 *	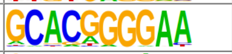	1e-5	-1.255e+01	7.81%	2.80%	262.7bp (286.8bp)	RO3G_00049(RRM)/Rhizopus_oryzae-RNCMPT00205-PBM/HughesRNA(0.727) More Information | Similar Motifs Found	motif file (matrix)
25 *	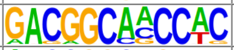	1e-5	-1.246e+01	2.40%	0.26%	331.5bp (268.5bp)	RCS1/RCS1_H2O2Hi/35-RCS1(Harbison)/Yeast(0.657) More Information | Similar Motifs Found	motif file (matrix)
26 *	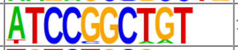	1e-4	-1.073e+01	2.40%	0.34%	208.1bp (268.6bp)	SPDEF/MA0686.1/Jaspar(0.741) More Information | Similar Motifs Found	motif file (matrix)
27 *	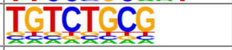	1e-4	-9.846e+00	6.31%	2.35%	241.4bp (265.5bp)	Smad4(MAD)/ESC-SMAD4-ChIP-Seq(GSE29422)/Homer(0.805) More Information | Similar Motifs Found	motif file (matrix)
28 *	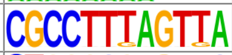	1e-3	-8.860e+00	0.60%	0.00%	114.9bp (164.7bp)	FNE(RRM)/Drosophila_melanogaster-RNCMPT00120-PBM/HughesRNA(0.688) More Information | Similar Motifs Found	motif file (matrix)
29 *	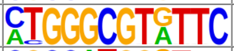	1e-3	-8.729e+00	0.90%	0.03%	25.7bp (278.0bp)	PB0143.1_Klf7_2/Jaspar(0.734) More Information | Similar Motifs Found	motif file (matrix)
30 *	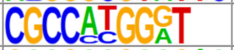	1e-3	-8.623e+00	2.70%	0.58%	206.3bp (265.2bp)	brk/dmmpmm(Bergman)/fly(0.678) More Information | Similar Motifs Found	motif file (matrix)
31 *	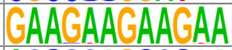	1e-3	-7.481e+00	2.10%	0.42%	161.0bp (337.7bp)	Unknown4/Arabidopsis-Promoters/Homer(0.783) More Information | Similar Motifs Found	motif file (matrix)
32 *	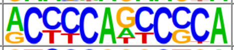	1e-2	-6.477e+00	6.61%	3.25%	240.9bp (275.1bp)	Zfp281(Zf)/ES-Zfp281-ChIP-Seq(GSE81042)/Homer(0.679) More Information | Similar Motifs Found	motif file (matrix)
33 *	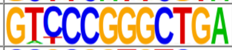	1e-1	-4.449e+00	0.60%	0.05%	107.0bp (264.3bp)	PUT3/MA0358.1/Jaspar(0.692) More Information | Similar Motifs Found	motif file (matrix)
34 *	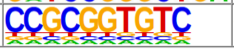	1e0	-6.920e-01	0.30%	0.21%	109.7bp (274.9bp)	PDR1/MA0352.1/Jaspar(0.708) More Information | Similar Motifs Found	motif file (matrix)

Fig S15. *De novo* motif enrichment analysis. **a**. Homer's *de novo* motif enrichment analysis output.

A

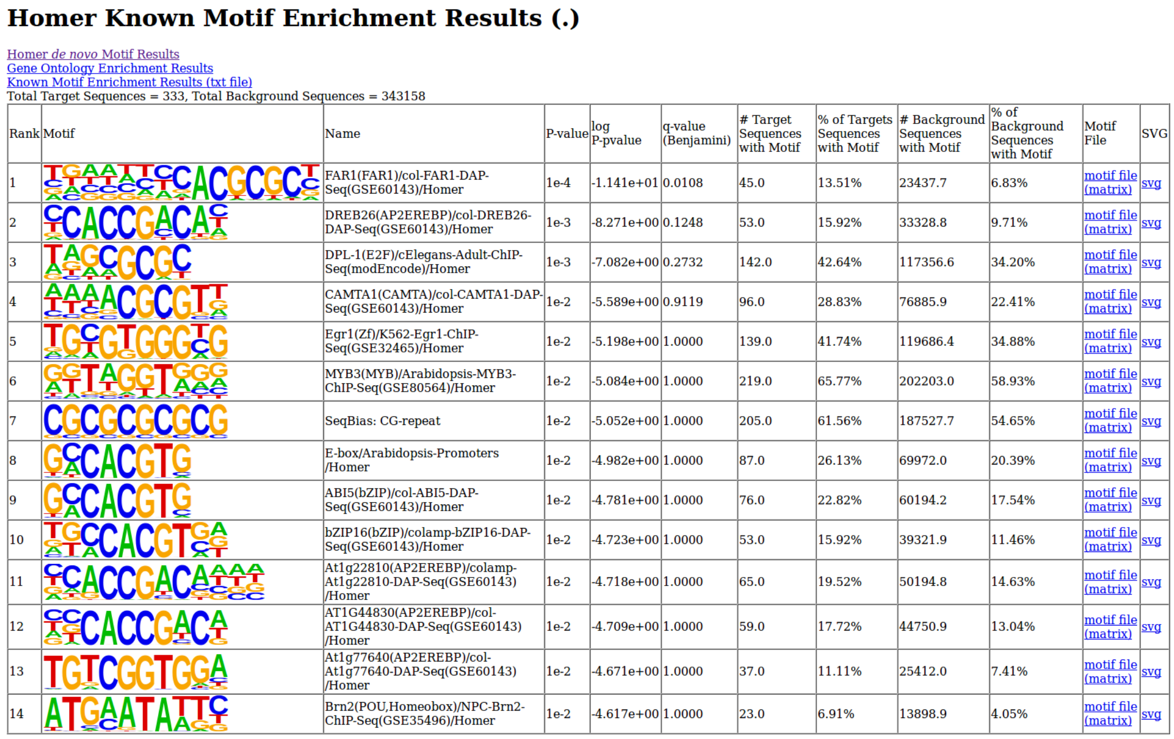

Homer Known Motif Enrichment Results (.)

Homer *de novo* Motif Results
Gene Ontology Enrichment Results
Known Motif Enrichment Results (txt file)
Total Target Sequences = 333, Total Background Sequences = 343158

Rank	Motif	Name	P-value	log P-pvalue	q-value (Benjamini)	# Target Sequences with Motif	% of Targets Sequences with Motif	# Background Sequences with Motif	% of Background Sequences with Motif	Motif File	SVG
1		FAR1(FAR1)/col-FAR1-DAP-Seq(GSE60143)/Homer	1e-4	-1.141e+01	0.0108	45.0	13.51%	23437.7	6.83%	motif file (matrix)	svg
2		DREB26(AP2EREBP)/col-DREB26-DAP-Seq(GSE60143)/Homer	1e-3	-8.271e+00	0.1248	53.0	15.92%	33328.8	9.71%	motif file (matrix)	svg
3		DPL-1(E2F)/cElegans-Adult-ChIP-Seq(modEncode)/Homer	1e-3	-7.082e+00	0.2732	142.0	42.64%	117356.6	34.20%	motif file (matrix)	svg
4		CAMTA1(CAMTA)/col-CAMTA1-DAP-Seq(GSE60143)/Homer	1e-2	-5.589e+00	0.9119	96.0	28.83%	76885.9	22.41%	motif file (matrix)	svg
5		Egr1(Zf)/K562-Egr1-ChIP-Seq(GSE32465)/Homer	1e-2	-5.198e+00	1.0000	139.0	41.74%	119686.4	34.88%	motif file (matrix)	svg
6		MYB3(MYB)/Arabidopsis-MYB3-ChIP-Seq(GSE80564)/Homer	1e-2	-5.084e+00	1.0000	219.0	65.77%	202203.0	58.93%	motif file (matrix)	svg
7		SeqBias: CG-repeat	1e-2	-5.052e+00	1.0000	205.0	61.56%	187527.7	54.65%	motif file (matrix)	svg
8		E-box/Arabidopsis-Promoters/Homer	1e-2	-4.982e+00	1.0000	87.0	26.13%	69972.0	20.39%	motif file (matrix)	svg
9		ABI5(bZIP)/col-ABI5-DAP-Seq(GSE60143)/Homer	1e-2	-4.781e+00	1.0000	76.0	22.82%	60194.2	17.54%	motif file (matrix)	svg
10		bZIP16(bZIP)/colamp-bZIP16-DAP-Seq(GSE60143)/Homer	1e-2	-4.723e+00	1.0000	53.0	15.92%	39321.9	11.46%	motif file (matrix)	svg
11		At1g22810(AP2EREBP)/colamp-At1g22810-DAP-Seq(GSE60143)/Homer	1e-2	-4.718e+00	1.0000	65.0	19.52%	50194.8	14.63%	motif file (matrix)	svg
12		AT1G44830(AP2EREBP)/col-AT1G44830-DAP-Seq(GSE60143)/Homer	1e-2	-4.709e+00	1.0000	59.0	17.72%	44750.9	13.04%	motif file (matrix)	svg
13		At1g77640(AP2EREBP)/col-At1g77640-DAP-Seq(GSE60143)/Homer	1e-2	-4.671e+00	1.0000	37.0	11.11%	25412.0	7.41%	motif file (matrix)	svg
14		Brn2(POU,Homeobox)/NPC-Brn2-ChIP-Seq(GSE35496)/Homer	1e-2	-4.617e+00	1.0000	23.0	6.91%	13898.9	4.05%	motif file (matrix)	svg

B

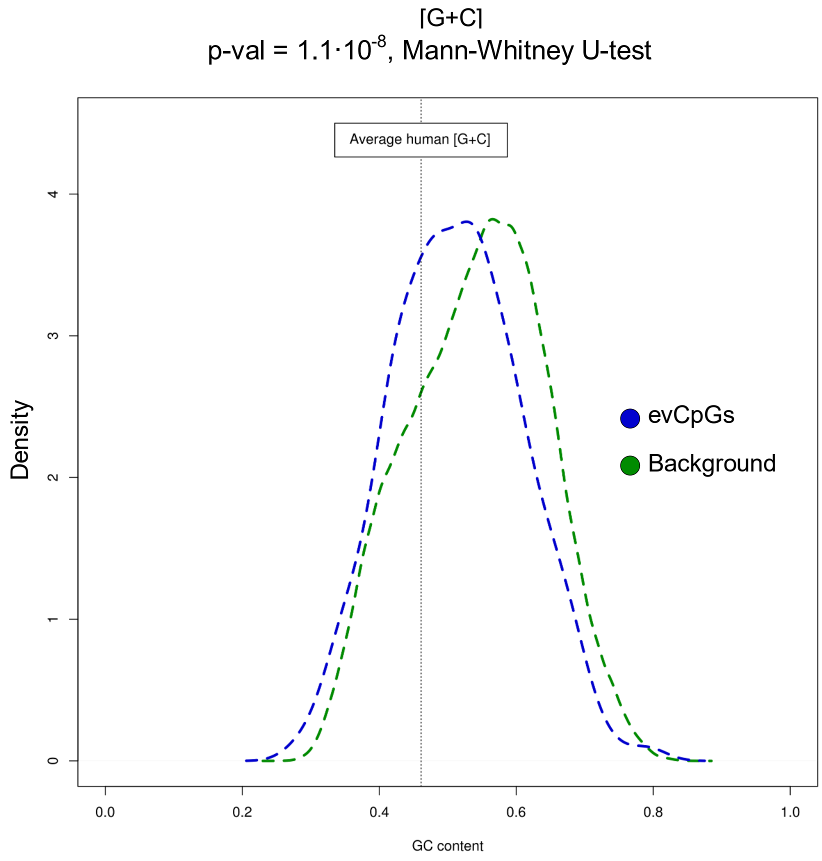

Fig S16. [G+C] content and known motif enrichment analysis. **a**. Homer's known motif enrichment analysis output. **b.** [G+C] content distribution in sequences ± 500 bp around evCpGs and the background CpGs.

A

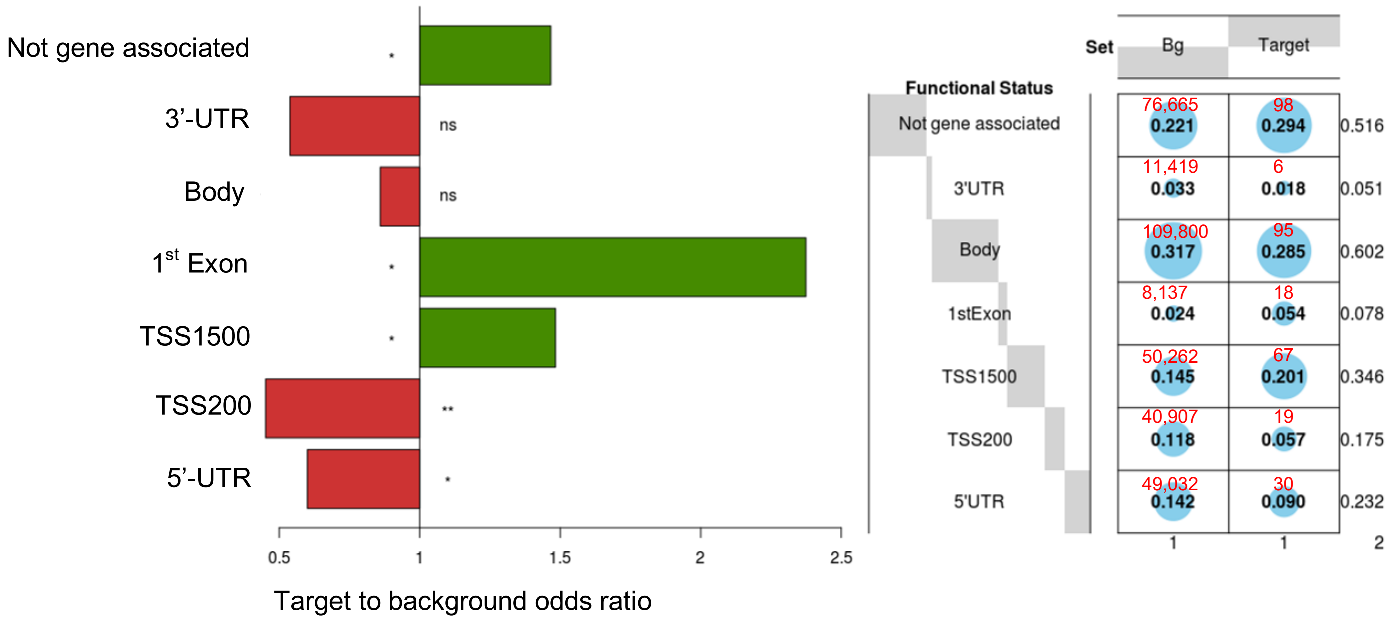

B

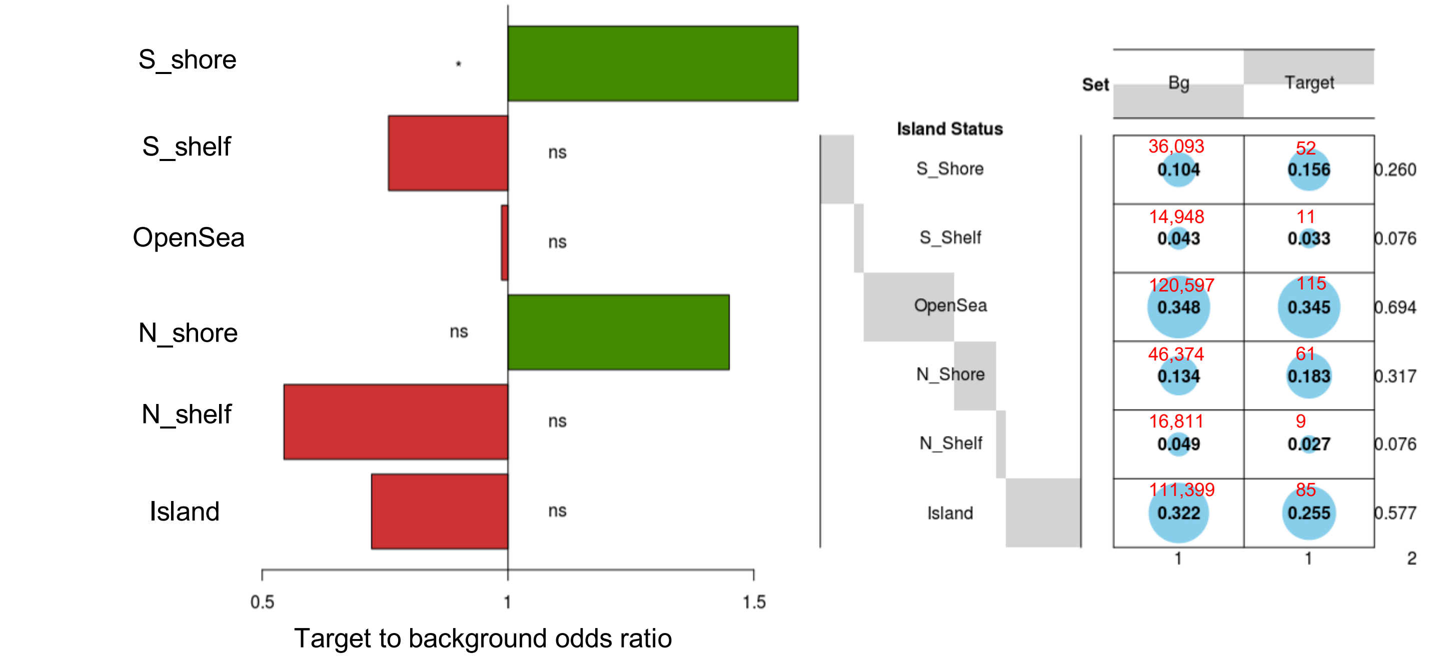

Fig S17. Functional and island status enrichment analysis of evCpGs **a**. CpG functional status and **b.** CpG island status enrichment analysis, where odds ratios and significance are represented on the left while relative counts are represented on the right (absolute counts are highlighted in red). ns: non-significant; *: $0.01 \leq$ p-val$_{Bonferroni} < 0.05$; **: $0.001 \leq$ p-val$_{Bonferroni} < 0.01$; ***: p-val$_{Bonferroni} < 0.001$.

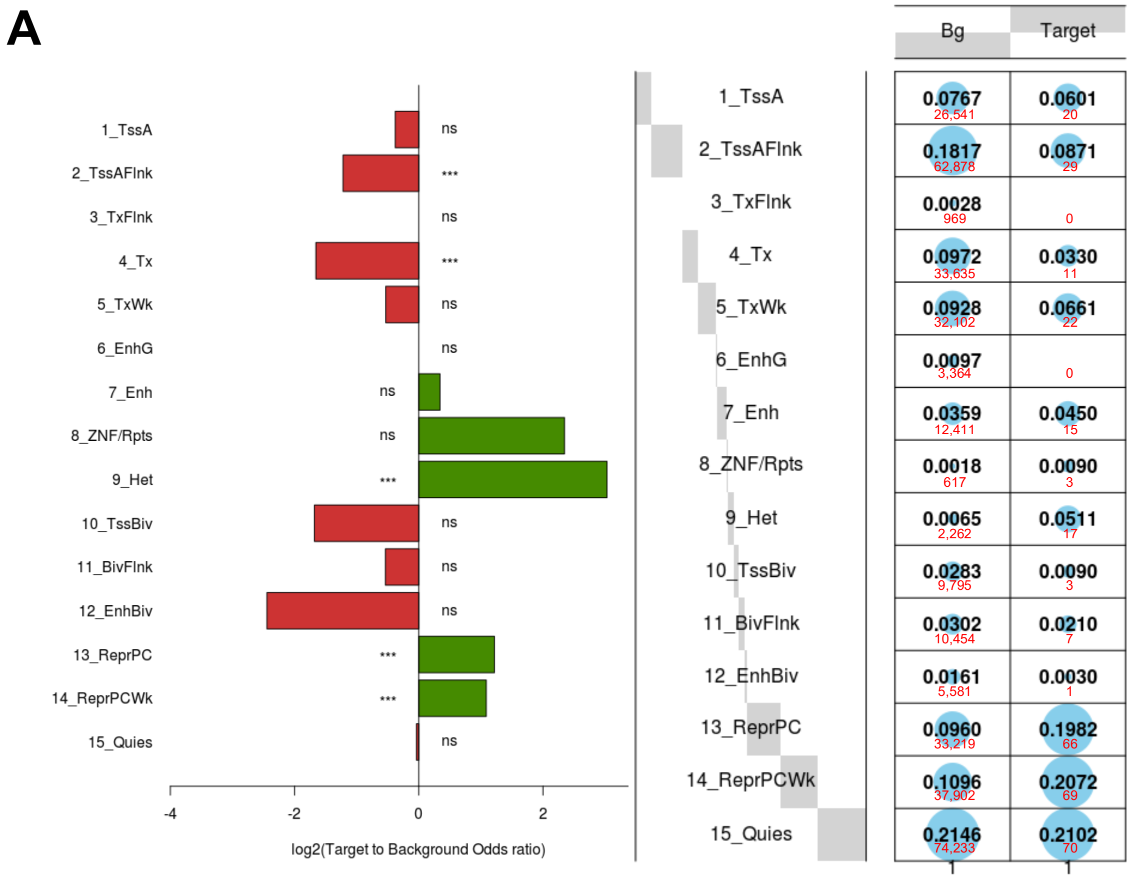

(1) *TssA: active TSS promoter;* ***(2)*** *TssAFlnk: flanking active TSS promoter;* ***(3)*** *TxFlnk: transcribed state at the 5' or 3' end of genes with promoter and enhancer signatures;* ***(4)*** *Tx: actively transcribed;* ***(5)*** *TxWk: weakly transcribed;* ***(6)*** *EnhG: genic enhancer;* ***(7)*** *Enh: enhancer states;* ***(8)*** *ZNF/Rpts: associated with zinc finger genes;* ***(9)*** *Het: constitutive heterochromatin;* ***(10)*** *TssBiv: bivalent/poised TSS;* ***(11)*** *BivFlnk: flanking bivalent TSS/Enhancer;* ***(12)*** *EnhBiv: bivalent enhancer;* ***(13)*** *ReprPC: repressed polycomb;* ***(14)*** *ReprPCWk: weak repressed Polycomb;* ***(15)*** *Quies: quiescent.*

Fig S18. PBMC 15-state HMM status enrichment analysis of evCpGs **a**. Chromatin functional state enrichment analysis, where $\log_2$ transformed odds ratios and significance are represented on the left while relative counts are represented on the right (absolute counts are highlighted in red). ns: non-significant; *: $0.01 \leq p\text{-val}_{Bonferroni} < 0.05$; **: $0.001 \leq p\text{-val}_{Bonferroni} < 0.01$; ***: $p\text{-val}_{Bonferroni} < 0.001$.

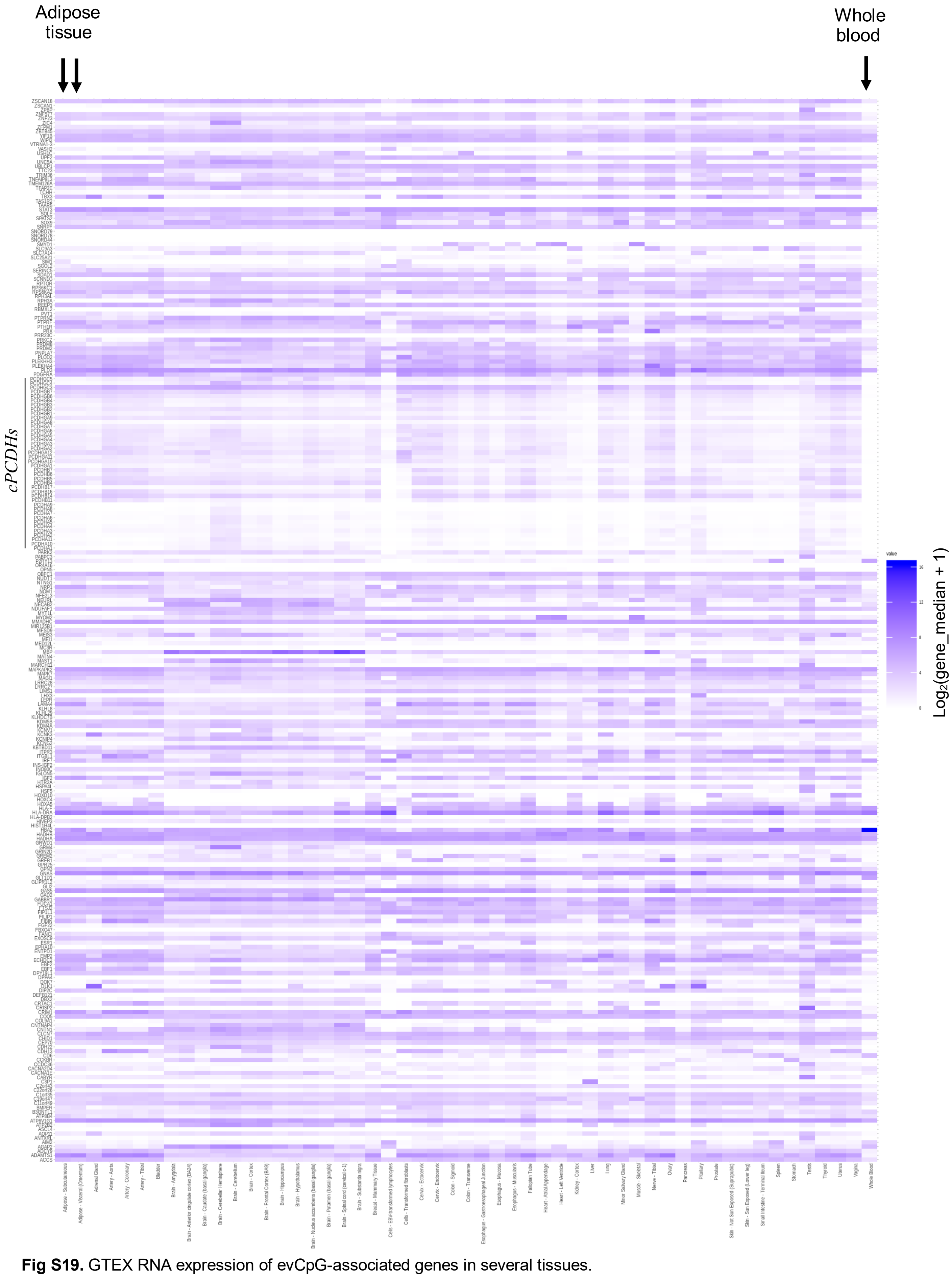

Fig S19. GTEX RNA expression of evCpG-associated genes in several tissues.

A

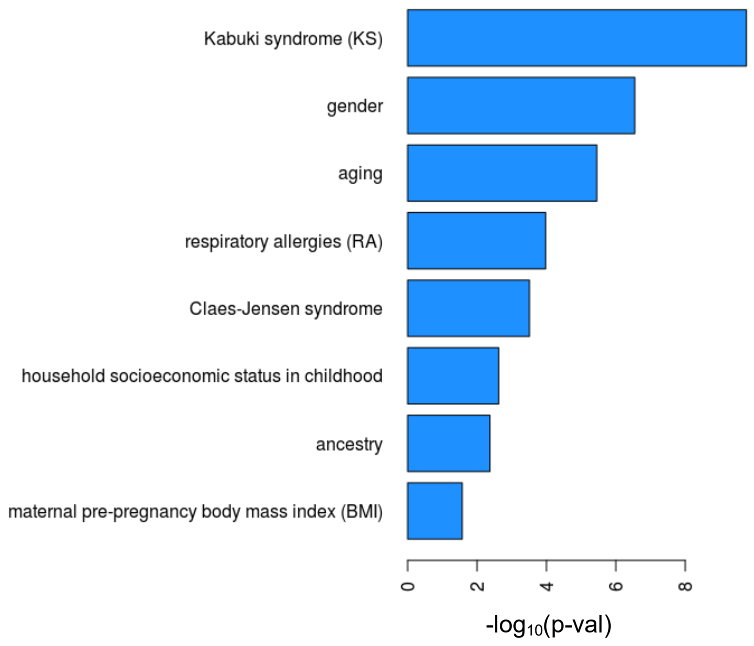

B

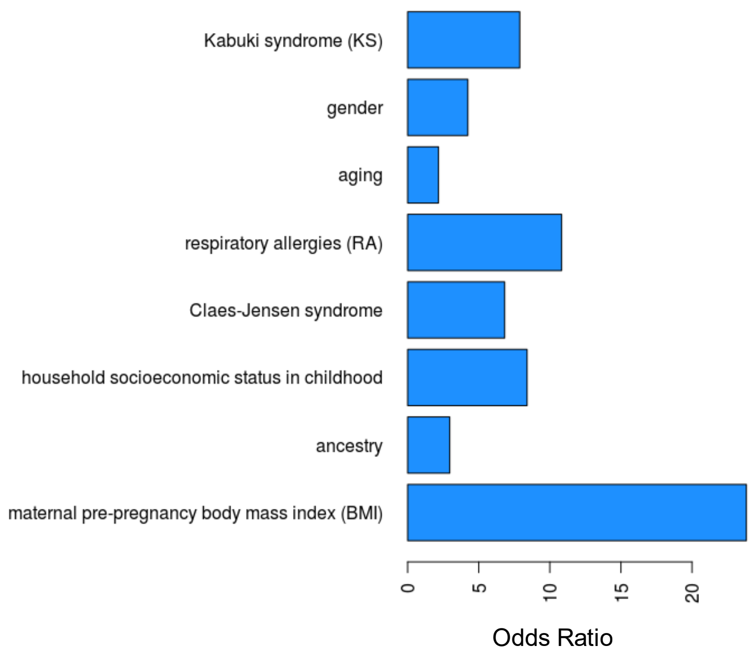

Fig S20. EWAS trait enrichment of evCpGs based on the EWAS atlas database and focusing on the traits that were associated to evCpGs. **a**. Fisher's exact test significance. **b**. Enrichment odds ratios.

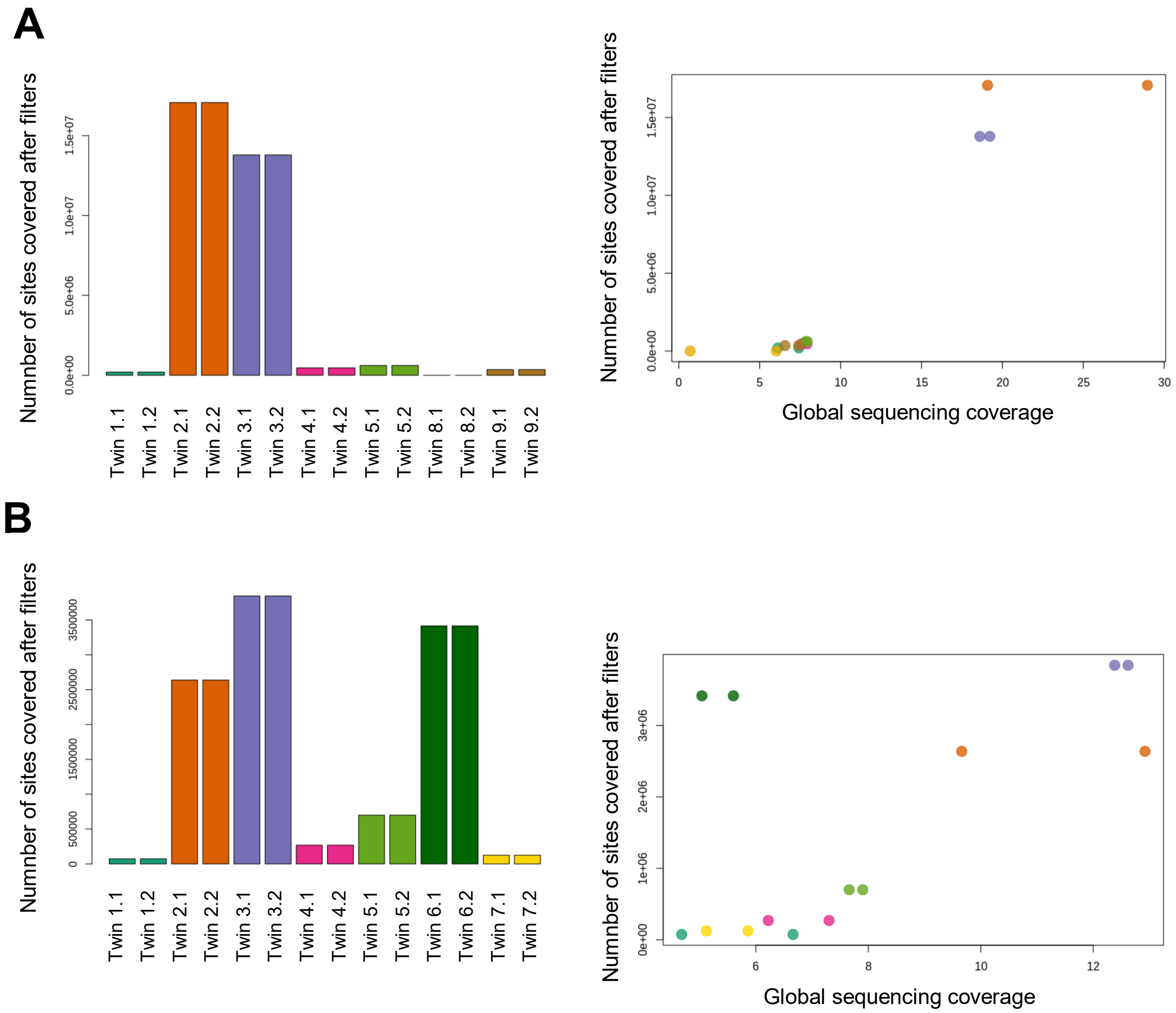

Fig S21. WGBS data visualization in **a.** whole blood and **b.** adipose tissue, in terms of number of methylation sites covered per MZ twin (colour-coded per pair) after preprocessing and this same number plotted against sequencing coverage.

Whole blood; α_{Bonfer} = 0.05/13

Twin pair		PCDH	Not PCDH
1	|Δβ| ≥ 0.4	1	36
	|Δβ| < 0.4	1,201	198,335
	Enrichment p-value = 0.2003		
2	|Δβ| ≥ 0.4	28	6,687
	|Δβ| < 0.4	11,172	17,049,974
	Enrichment p-value = 4.785e-14		
3	|Δβ| ≥ 0.4	46	4,628
	|Δβ| < 0.4	17,928	13,763,879
	Enrichment p-value = p-value < 2.2e-16		
4	|Δβ| ≥ 0.4	7	107
	|Δβ| < 0.4	2,669	462,192
	Enrichment p-value = 4.993e-06		
5	|Δβ| ≥ 0.4	0	153
	|Δβ| < 0.4	3,557	606,724
	Enrichment p-value = 1		
8	|Δβ| ≥ 0.4	NA	NA
	|Δβ| < 0.4	NA	NA
	Enrichment p-value = NA; **no sites were common between twins**		
9	|Δβ| ≥ 0.4	0	73
	|Δβ| < 0.4	1,219	349,424
	Enrichment p-value = 1		

Fig S22. Enrichment analysis in whole blood WGBS data. Relative counts for methylation sites showing differences ≥ 0.4 and < 0.4 in the *cPCDH* loci or outside. Absolute counts are highlighted in red.

Adipose tissue; α_{Bonfer} = 0.05/13

Twin pair		PCDH	Not PCDH
1	$\lvert\Delta\beta\rvert \geq 0.4$	0	20
	$\lvert\Delta\beta\rvert < 0.4$	666	73,291
	Enrichment p-value = 1		
2	$\lvert\Delta\beta\rvert \geq 0.4$	14	680
	$\lvert\Delta\beta\rvert < 0.4$	17,699	2,619,808
	Enrichment p-value = 0.0003332		
3	$\lvert\Delta\beta\rvert \geq 0.4$	28	1045
	$\lvert\Delta\beta\rvert < 0.4$	23,238	3,818,830
	Enrichment p-value = 2.896e-10		
4	$\lvert\Delta\beta\rvert \geq 0.4$	1	63
	$\lvert\Delta\beta\rvert < 0.4$	2,130	267,244
	Enrichment p-value = 0.3985		
5	$\lvert\Delta\beta\rvert \geq 0.4$	3	162
	$\lvert\Delta\beta\rvert < 0.4$	4,573	695,447
	Enrichment p-value = 0.09464		
6	$\lvert\Delta\beta\rvert \geq 0.4$	11	813
	$\lvert\Delta\beta\rvert < 0.4$	14,769	3,399,522
	Enrichment p-value = 0.001143		
7	$\lvert\Delta\beta\rvert \geq 0.4$	0	14
	$\lvert\Delta\beta\rvert < 0.4$	917	123,718
	Enrichment p-value = 1		

Fig S23. Enrichment analysis in adipose tissue WGBS data. Relative counts for methylation sites showing differences ≥ 0.4 and < 0.4 in the *cPCDH* loci or outside. Absolute counts are highlighted in red.
