# Supplementary material for: Equivalent DNA methylation variation between monozygotic co-twins and unrelated individuals reveals universal epigenetic inter-individual dissimilarity
Source: Genome Biol. 2021 Jan 5;22:18. doi: 10.1186/s13059-020-02223-9 (PMC7786996; doi:10.1186/s13059-020-02223-9)
Supplement: Supplementary file 3 — Additional file 3. Supplementary methods. [file 13059_2020_2223_MOESM3_ESM.docx]

**Supplementary methods**

**Equivalent DNA methylation variation between monozygotic co-twins and unrelated individuals reveals universal epigenetic inter-individual dissimilarity**

Benjamin Planterose Jiménez, Fan Liu, Amke Caliebe, Diego Montiel González, Jordana T. Bell, Manfred Kayser, Athina Vidaki

**Table of contents**

**1. Datasets 3**

**2. 450K data processing 5**

**2.1 Thorough quality control for datasets including IDATs 5**

**2.2 Limited quality control for pre-processed datasets 7**

**2.3 Whole blood data pre-processing from IDATs 8**

**2.4 Adipose tissue data pre-processing from IDATs 11**

**2.5 Data extraction and preparation for pre-processed datasets 12**

**3. evCpG discovery 13**

**3.1 Statistical approach 13**

**3.2 Visualisation 16**

**4. Assessing evCpGs’ measurement error in blood 19**

**4.1 450K technical measures 19**

**4.1.1 Number of beads 19**

**4.1.2 Detection p-value 20**

**4.1.3 Intra-class correlation coefficient 21**

**4.2 Technical replicates and short-term stability 21**

**5. evCpGs’ epigenetic clock/drift in blood 22**

**6. Behavior of evCpGs in other tissues 23**

**6.1 Post-mortem tissues 23**

**6.2 Replication of evCpGs in adipose tissue 23**

**7. Functional annotation of evCpGs 25**

**7.1 Enrichment analysis 25**

**7.1.1 Imprinted genes, metastable epialleles, EWAS traits and mQTL 26**

**7.1.2 Functional and CpG island status 26**

**7.1.3 PBMC 15-states ChromHMM 27**

**7.1.4 Gene ontology terms 28**

**7.1.5 DNA motifs and [G+C] content 28**

**7.1.6 Genomic position 29**

**7.2 RNA expression of evCpG-associated genes 29**

**8. Whole genome bisulfite sequencing 30**

**References 31**

**1. Datasets**

The following datasets were employed for this study:

- (*dataset-A*) Environmental Risk (E-risk) Longitudinal Twin Study (British, raw 450K, GSE105018 (GEO), 426 MZ twin pairs, whole blood, samples collected at age 18, 48.6% females) [1].
- (*dataset-B*) Danish Twin Registry (Danish, raw 450K, GSE61496 (GEO), 146 MZ twin pairs, whole blood, mean age = 48.4 years, s.d. = 15.5 years, 47.9% females) [2]. Additionally, three of the twin pairs had technical replicates for both twins (duplicates for two and tetrads for one twin pair). In total, 16 possible combinations of technical duplicates could be computed: $\sum_{i=1}^{n_{R}} \binom{n_{i}}{2}=4\cdot\binom{2}{2}+2\cdot\binom{4}{2}=16$; $n_{R}$: number of twins with replicates; $n_{i}$: number of replicates for individual $i$.
- (*dataset-C1*) Zhang *et al.* dataset in blood, group A (Chinese, pre-processed 450K, GSE51388 (GEO), 10 pairs of MZ twins (1 twin individual with 5 technical replicates), whole blood, mean age = 41.3 years, s.d. = 14.7 years, 40% females) [3].
- (*dataset-C2*) Zhang *et al.* dataset in blood, group B (Chinese, pre-processed 450K, GSE51388 (GEO), 1 pair of MZ twins and 6 unrelated individuals (one individual with 6 technical replicates at one time point), whole blood, mean age = 29.3 years, s.d. = 5.8 years, 37.5% females) [3]. The individuals here included differ from group A (*dataset C-1*) and encompasses time points 0, 3, 6 and 9 months.
- (*dataset-D*) Cross-sectional children dataset (Chinese, raw 450K, GSE104812 (GEO), 48 individuals, whole blood, mean age = 9.04 years, s.d. = 1.73 years. 39.6% females) [4].
- (*dataset-E*) The Northern Sweden Population Health Study (NSPHS) dataset with a wide age interval (Swedish, raw 450K, GSE87571 (GEO), 727 individuals, whole blood, mean age = 47.4 years, s.d. = 20.9 years, 53% females) [5].
- (*dataset-F*) TwinsUK (British, raw 450K, 328 MZ twin pairs, whole blood, mean age = 57.9 years, s.d. = 10.3 years, 100% females) [6]. The data is partially available in GSE121633 and in at GSE62992 (GEO).
- (*dataset-G*) Early Nutrition and Immune Development (ENID) Trial children cohort (Gambian, raw 450K, GSE99863 (GEO), 240 children aged 2 years, whole blood, 48.6% females) [7].
- (*dataset-H*) Cross-tissue variation dataset (Estonian, pre-processed 450K, GSE50192 (GEO), 4 individuals, wide variety of post-mortem tissues, mean age = 51.8 years, s.d. = 8.4 years, 25% females) [8]. A total of 17 different somatic tissues are included in the panel: abdominal and subcutaneous adipose tissue, bone, joint cartilage, yellow and red bone marrow, coronary and splenic artery, abdominal and thoracic aorta, gastric mucosa, lymph node, tonsils, bladder, gall bladder, medulla oblongata and sciatic nerve.
- (*dataset-I*) TwinsUK cross-tissue replication dataset (British, preprocessed 450K, E-MTAB-1866 (ArrayExpress), 97 MZ twin pairs, subcutaneous adipose tissue, mean age = NA years, s.d. = NA years, 100% females) [9].
- (*dataset-J*) Longitudinal dataset in fat (Finnish, raw 450K, GSE103768 (GEO), 19 individuals at time points 0, 5 and 12 months, subcutaneous adipose tissue, mean age = 35.2 years, s.d. = 1.8 years, 63.1% females) [10].
- (*dataset-K1*) TwinsUK study (British, whole-genome bisulfite sequencing, E-MTAB-3549 (ArrayExpress), 7 MZ twin pairs, whole blood, mean age = 59.1 years, s.d. = 8.9 years, 100% females) [11]. 5 twin pairs are common with *dataset-K2*. We employ the same labels as the authors: Twins 1, 2, 3, 4, 5, 8 and 9.
- (*dataset-K2*) TwinsUK cohort (British, whole-genome bisulfite sequencing, E-MTAB-3549 (ArrayExpress), 7 MZ twin pairs, adipose tissue, mean age = 60.7 years, s.d. = 10.4 years, 100% females) [11]. 5 twin pairs are common with *dataset-K1*. We employ the same labels as the authors: Twins 1, 2, 3, 4, 5, 6 and 7.

**2. 450K data processing**

All data analysis was performed in R 3.4.4 (“Someone to Lean on”) [12]. We employed the libraries minfi [13], ENmix [14], wateRmelon [15], missMethyl [16] for reading IDAT files and for performing normalization and quality control for all 450K data. For publicly available data derived from the GEO database, phenotypes were parsed with the help of GEOquery [17].

**2.1 Thorough quality control for datasets including IDATs**

The following approach could only be performed for datasets with IDAT files available (*datasets A-E* and *J*). Low-quality probes and samples were assessed with ENmix::QCinfo with parameters detPthre = 10^-6^, nbthre = 3, samplethre = 0.05, CpGthre = 0.05 and outlier = TRUE (**Fig S1**). For all datasets, we predicted sex with minfi::getSex and compared it to phenotypic sex in order to avoid mix-ups (**Fig S2**).

Discarded samples are reported below:

- (*dataset-A*) From the 1658 samples in GSE105018 (GEO), 852 samples were used as they corresponded to MZ samples (612 corresponded to dizygotic and 194 have NA as zygosity).
- (*dataset-B*) From the 312 samples in GSE61496 (GEO), 302 samples were used (including technical replicates). The eliminated files (GSM1506315, GSM1506430, GSM1506317, GSM1506529, GSM1506321, GSM1506535, GSM1506323, GSM1506543, GSM1506424 and GSM1506549) either had bad quality, missing values in the phenotype or were a co-twin of an eliminated sample.
- (*dataset-D*) No samples out of the 48 were discarded due to low-quality or predicted sex discordancy.
- (*dataset-E*) From the 732 samples in GSE87571 (GEO), 727 samples were used. The eliminated files (GSM2334328, GSM2334261 GSM2334015, GSM2334619 and GSM2334342) either had bad quality or missing phenotype (age or sex).
- (*dataset-F*) From the 660 samples that we obtained by applying to TwinsUK cohort data access (<https://twinsuk.ac.uk/resources-for-researchers/access-our-data/>), 656 samples were used. The eliminated files (6929793057_R06C02, 6929793137_R01C02, 6929793137_R01C01 and 6929793137_R05C02) either had bad quality or were a co-twin of an eliminated sample.
- (*dataset-G*) From the 257 samples in GSE99863 (GEO), 240 samples were used. The eliminated files (GSM2656366, GSM2656358, GSM2656200, GSM2656251, GSM2656253, GSM2656264, GSM2656266, GSM2656317, GSM2656368, GSM2656357, GSM2656427, GSM2656378, GSM2656393, GSM2656193, GSM2656202, GSM2656223 and GSM2656295) either had bad quality, consisted of technical replicates or presented sex that was discordant with the predicted sex.
- (*dataset-J*) No samples out of the 57 were discarded due to low-quality or predicted sex discordancy.

With respect to discarded probes:

We excluded all low-quality probes and CpGs associated to X- (n = 11,232) and Y-chromosomes (n = 416) based on IlluminaHumanMethylation450kmanifest R-package. The number of excluded low-quality probes per dataset are the following: *dataset-A* (n = 2,561), *dataset-B* (n = 4,545), *dataset-D* (n = 1,509), *dataset-E* (n = 2,843), *dataset-F* (n = 2,800), *dataset-G* (n = 3,807) and *dataset-J* (n = 2,382).

With respect to our SNP policy, we employed the SNPs.147CommonSingle file from R-package IlluminaHumanMethylation450kanno.ilmn12.hg19 which is based on dbSNP v.147. We excluded all probes containing SNPs at the CpG or in the single base extension site (the latter solely for type I probes) and probes containing SNPs with minor allele frequency > 0.01 within the probe (n = 99,337). With respect to cross-reactive probes, we excluded all CpGs predicted *in silico* to be cross-hybridizing as reported by Chen *et al* and Price *et al* (n = 41,993) [18, 19]. We should mention that there is a considerable intersection between the different sets of excluded probes.

**2.2 Limited quality control for pre-processed datasets**

Limited 450K quality control was performed on pre-processed datasets (datasets G-I). Pre-processed data was preferred only when no similar publicly available dataset had been deposited in raw IDAT form and never for core results in the manuscript. The quality control that can be performed on pre-processed data is inferior, as the information regarding internal 450K control probes (SNP, out-of-band, bisulfite conversion probes, etc) has been discarded. Also, but in an irregular fashion depending on the choice of authors for depositing the dataset, additional information is often unavailable, such as detection p-value and beads-per-probe matrices, separate intensity channels, CpG-SNPs or even sex chromosome CpGs, the latter required for checking for sex mismatches. Finally, the use of different normalization methodology injects additional variation that makes it hard to establish direct comparisons across datasets. No samples were discarded from these datasets, resulting in the following total number per dataset: *dataset-C1*: 24, *dataset-C2*: 36, *dataset-H*: 70 and *dataset-I*: 194.

**2.3 Whole blood data preprocessing from IDATs**

Prior to normalization, we extracted the Reinius *et al* [20] isolated cell type reference raw data in the form of an RGChannelSet via the FlowSorted.Blood.450k R-package. An equivalent RGChannelSet object was obtained for the dataset-to-analyze (DTA) with the function minfi::read.metharray.exp (for *datasets*-A, B, D, E, F and G). They were both merged with the function minfi::combineArrays. We then normalized all together with three different methods: dasen, stratified quantile normalization and oob_RELIC_QN_BMIQ. The functions employed were *(i)* waterRmelon::dasen (modified to skip Sentrix position background linear gradient model), *(ii)* minfi::preprocessQuantile and *(iii)* ENmix::preprocessENmix with parameters bgParaEst = “oob” and dyeCorr = “RELIC”, ENmix::norm.quantile with parameter method = “quantile1” and ENmix::bmiq.mc. Employing several normalization methods is not a standard routine in epigenome-wide studies, but in our study it was initially included as another quality control step. We did notice strong differences between the methods (**Fig S3-4**, **Fig S6**); thus, in order to avoid normalization method-specific outcomes, we decided to search for significant results across multiple normalization strategies. There are dozens of normalization methods for 450K data. We considered only popular methods and with different orders of computational complexity. Briefly:

• Dasen [15]: first equalizes backgrounds for type I and type II, then performs quantile normalization on the M/U intensity stratifying by probe type.

• SQN [13]: first fixes outliers in the U/M intensities, then performs quantile normalization on the M/U intensities stratifying by CpG-island relationship (CpG Island/shore/shelf/open sea).

• Oob_RELIC_QN_BMIQ [14]: first corrects the background by modelling it as an exponential-normal mixture distribution from type I-green in the red channel or type I-red in the green channel, e.g. out-of-band probes (oob). It then performs dye bias correction with Regression on Logarithm of Internal Control probes (RELIC), then performs quantile normalization on the M/U intensity stratifying by probe type and finally, performs Beta mixture quantile dilation (BMIQ) to correct bias between type I and type II probes.

For all methods, beta values were computed via:

$$\beta=\frac{M}{U+M+\alpha}$$

Where $\beta$ is the beta-value or methylation level, $M$ is the fluorescence intensity for methylated epiallele, $U$ is the fluorescence intensity for unmethylated epiallele and$\alpha$ an offset for numerical stability (hereby set at 100). For normalization *(ii)*, given that the function minfi::getBeta did not seem to accept an offset argument for GenomicRatioSet objects (minfi 1.25.1), we had to apply the offset via:

$$\beta_{\alpha=100}=\frac{\beta_{\alpha=0}\cdot2^{CN}}{2^{CN}+\alpha}=\beta_{\alpha=0}\cdot2^{CN}\cdot{(2^{CN}+\alpha)}^{-1}$$

Where interpunct symbolizes element-wise matrix multiplication, where $\beta_{\alpha=0}$ and $\beta_{\alpha=100}$ are the methylation matrices with offset of 0 and 100 (CpGs as rows and samples as columns), where CN is the copy number matrix (CpGs as rows and samples as columns) easily extractable via minfi::getCN which computes:

$$CN=\log_{2} (U+M)$$

Since the array design is confounded for the isolated cell type references, we could not correct their chip batch effects. We did correct chip batch effects on the DTA with sva::ComBat which was supplied with the available covariates: *dataset-A* (sex), *dataset-B* (sex, age), *dataset-E* (sex, age), *dataset-F* (age, BMI, smoking) and *dataset-G* (sex). We did not correct for batch effects on the dataset of Chinese children (*dataset-D*), as chip array was not supplied by the authors. Finally, we corrected cell composition differences via a modified Houseman method [21] as implemented in minfi [13] (**Fig S5**); we performed a discovery of cell composition sensitive probes based on minfi:::pickCompProbes (modified to accept a beta-value matrix from any normalisation) and we estimated the cell counts with minfi:::projectCellType. We defined the reference composition as the average cell composition across the dataset. We then adjusted probes towards the reference composition with the following linear algebra expression:

$$B_{corrected}=B_{uncorrected}- R\times\Delta\alpha$$

Where $\times$ symbolizes matrix multiplication, where $B_{corrected}$ and $B_{uncorrected}$ are beta-value matrices prior and after correction (CpGs as rows and samples as columns), $R$ is a matrix containing the average CpG methylation for the isolated cell type references (CpGs as rows and number of isolated cell types as columns) and $\Delta\alpha$ is a matrix of the differences in cell composition for every sample with respect to the average reference composition (number of isolated cell types as rows and samples as columns). Only CpGs with p-value (F-test) < 10^-8^ were adjusted for cell composition, by setting the rows of $R$ to zero for CpGs with p-value > 10^-8^.

**2.4 Adipose tissue data preprocessing from IDATs**

For *dataset-J*, we read the raw IDAT files with the function minfi::read.metharray.exp. We then normalized stratified quantile normalization with minfi::preprocessQuantile.

Beta values were computed via:

$$\beta=\frac{M}{U+M+\alpha}$$

With an offset$\alpha$ of 100. Again, given that the function minfi::getBeta did not seem to accept an offset argument for GenomicRatioSet objects (minfi 1.25.1), we had to apply the offset via:

$$\beta_{\alpha=100}=\frac{\beta_{\alpha=0}\cdot2^{CN}}{2^{CN}+\alpha}=\beta_{\alpha=0}\cdot2^{CN}\cdot{(2^{CN}+\alpha)}^{-1}$$

Where interpunct symbolizes element-wise matrix multiplication, where $\beta_{\alpha=0}$ and $\beta_{\alpha=100}$ are the methylation matrices with offset of 0 and 100 (CpGs as rows and samples as columns), where CN is the copy number matrix (CpGs as rows and samples as columns) easily extractable via minfi::getCN which computes:

$$CN=\log_{2} (U+M)$$

We did not correct chip batch effects with sva::ComBat this time given that the chip array design has not been shared. We did not correct for cell composition differences given that no method has been described in the literature to estimate and correct for cell composition differences in adipose tissue.

**2.5 Data extraction and preparation for pre-processed datasets**

For *dataset-C1/C2,* the authors had made their final processed data available in GEO. We extracted normalized values via the GEOquery R-package. The authors extracted the information from the IDATs with Genome studio without background correction and performed color bias correction + QN + BMIQ + batch effect corrections employing the R-package lumi.

For *dataset-H*, the authors did not provide either raw IDAT files or their final processed dataset. Instead, they shared the separate M/U intensity channels and the raw beta values extracted by GenomeStudio (e.g. partially processed data). On this note, on the one hand, we could not perform normalization with minfi or ENmix as these strictly require IDAT files. On the other hand, although wateRmelon::dasen can be deployed on the separate M/U intensity channels, these were named after the individual (instead of individuals and tissue) so there was no way to match tissues to samples. In the end, we extracted the raw beta values (properly named after individual and tissue) via the GEOquery R-package. We excluded probes for which 5% of the samples had a detection p-value > 1E-06 and performed quantile normalization with preprocessCore::normalize.quantiles. No batch effect correction could be performed given that the microarray chip design was not shared either.

For *dataset-I*, we downloaded the final pre-processed data from ArrayExpress E-MTAB-1866 (MuTHER_Fat_450K_norm_AE_030913.txt). The authors had extracted the information from the IDATs with Genome studio and performed QN on separate channels and probe type (similar to wateRmelon::dasen).

**3. evCpG discovery**

**3.1 Statistical approach**

We firstly excluded non-variable CpGs. The intra-class correlation coefficient (ICC) measures the proportion of non-technical variance compared to the total variance. An ICC of zero indicates that 100% of the variance could be explained by technical variance. In the 450K array, probes displaying ICC close to zero are common. We made use of the ICC coefficients derived from the Atherosclerosis Risk in Communities Study, which was computed based on 265 technical replicates. We decided to exclude CpGs that displayed less than the suggested ICC empirical cut-off of 0.37 [22]. We additionally excluded CpGs that displayed an inter-quantile range (IQR) of less than 0.07 in at least one normalization. Such IQR empirical threshold value was devised based on the rounded expected IQR derived from a beta distribution with mean $\mu$ equal to 0.5 (conservative as it corresponds to the maximum variance) and standard deviation $\sigma$ equal to 0.05 (which corresponds to the technical error that some authors report in the literature for targeted methods). In order to simulate it, we had to compute the beta distribution’s shape parameters as a function of $\mu$ and $\sigma$. The following expressions were employed:

$$\alpha=\left( \frac{1-\mu}{\sigma^{2}}- \frac{1}{\mu} \right)\cdot\mu^{2}; \beta= \alpha\cdot\left( \frac{1}{\mu}-1 \right)$$

For a total of 4,652 CpGs, absolute difference in methylation were computed for E-risk twin pairs (n = 426) and on all combination of unrelated pairs. The latter can be computed as:

$$n_{total combinations}-n_{twin pair combinations}=\binom{426\cdot2}{2}-426=362,100 unrelated pairs$$

We tested for equivalence of the distribution of the beta values between MZ pairs and unrelated pairs under the paradigm of equivalence testing with two one-sided tests (TOST). We used a robust TOST which does not require normality of the data. It is based on Yuen t-test, which evades non-normality by employing winsorized variance and trimmed mean [23]. We employed the wrappers supplied by equivalence::rtost with parameters Trim = 0.2. The parameter epsilon ($\varepsilon$), which characterizes the resolution at which the difference in two means can be defined as equivalent, was established per normalization; we justify this choice as the |Δβ| distribution in twin/unrelated pairs highly differed between normalizations (especially between oob_RELIC_QN_BMIQ compared to SQN or dasen). We based the selection of epsilon solely on the distribution of the trimmed mean of |Δβ|^twin^ across all tested CpGs. The criterion selected was the following:

$$\varepsilon={Median}_{i}({Tr}_{j}({{|\Delta\beta|}_{ij}}^{MZ twins}))$$

Where${{|\Delta\beta|}_{ij}}^{MZ twins}$ is the matrix of absolute difference of methylation ($i$ CpGs as rows and $j$ twin pairs as columns),${Tr}_{j}$ is the trimmed mean operator across$j$ twin pairs and ${Median}_{i}$ is the median operator across $i$ CpGs. The epsilon parameters that were finally chosen were $\varepsilon_{dasen}=0.02706526$, $\varepsilon_{StrQN}=0.02739861$ $\varepsilon_{oob\_RELIC\_QN\_BMIQ}=0.03566612$.

We defined evCpGs as CpGs that are significant across all three normalizations (α = 0.05/4,652 = 1.07×10^-5^; Bonferroni-corrected) which is equivalent to:

$$p-value\left( {CpG}_{i} \right)={max}_{norm k}({p-value}_{norm k}\left( {CpG}_{i} \right))$$

However, a limitation in our statistical approach is that we artificially enhance our dataset by exploring all combinations of unrelated pairs in the computation of |Δβ|_unrelated_, sacrificing non-independence of observations and possibly inflating the statistic. In order to make sure that this strategy has not jeopardized the discovery phase, we performed additional analyses on the evCpG set. In order to do so, we firstly devised an algorithm to access all possible pairing or matchings of unrelated individuals which obey the following restrictions: i) twin pairs are not allowed (hence, only unrelated individuals) ii) every individual may appear once (hence, avoiding violation of independence of observations). To begin with, a twin matching may be arranged as:

|  | *j* | |
| --- | --- | --- |
| *i* | **Twin *i*1** | **Twin *i*2** |
|  | Twin 11 | Twin 12 |
|  | Twin 21 | Twin 22 |
|  | Twin 31 | Twin 32 |
|  | … | … |

Where Twin*_ij_* corresponds to Twin pair *i* with twin ID *j*.

1) Firstly, we shuffle the rows:

| **Twin A** | **Twin B** |  | **Twin A** | **Twin B** |
| --- | --- | --- | --- | --- |
| Twin 11 | Twin 12 |  | Twin 31 | Twin 32 |
| Twin 21 | Twin 22 | → | Twin 21 | Twin 22 |
| Twin 31 | Twin 32 |  | Twin 11 | Twin 12 |
| … | … |  | … | … |

2) Secondly, we randomly exchange columns per pair:

| **Twin A** | **Twin B** |  | **Twin A** | **Twin B** |
| --- | --- | --- | --- | --- |
| Twin 11 | Twin 12 |  | Twin 32 | Twin 31 |
| Twin 21 | Twin 22 | → | Twin 21 | Twin 22 |
| Twin 31 | Twin 32 |  | Twin 11 | Twin 12 |
| … | … |  | … | … |

3) Finally, we shift the second whole column one position:

| **Twin A** | **Twin B** |  | **Twin A** | **Twin B** |
| --- | --- | --- | --- | --- |
| Twin 32 | Twin 31 |  | Twin 32 | Twin *ni* |
| Twin 21 | Twin 22 | → | Twin 21 | Twin 31 |
| Twin 11 | Twin 12 |  | Twin 11 | Twin 22 |
| … | … |  | … | Twin 12 |

Such algorithm can access all possible arrangements of non-twin matchings that fulfil our restrictions. Equivalence testing was executed again for the total 333 evCpGs and 333 randomly selected non-significant CpGs (controlCpGs) for 100 unrelated matching combinations (B). Similar to the discovery, the same three normalizations were employed and combined via the maximum function, deployed for each B-CpG pairs. The same significance threshold used in the discovery was applied at the re-testing stage (α = 0.05/4,652 ≈ 1.07 × 10^-5^, **Fig S7**). Summary statistics for the discovery and validation are reported on the file S1.

**3.2 Visualisation**

Manhattan plots were plotted by repurposing the code from qqman::manhattan initially designed for GWAS studies rather than epigenome-wide studies.

In order to assess the agreement between MZ twins, we decided not use correlation as: i) it does not directly assess the agreement of the data towards the unit line (*i.e.* y = x) but to the best fitting linear model, ii) it depends on how twin pairs A and B are assigned to the x- and y-axis and iii) its variance grows as it gets closer to zero. For this reason, we choose a more appropriate measure to evaluate concordance between MZ twins. We first defined the mean methylation value estimate for a given CpG in twin pair $i$, $\hat{\beta_{i}}$, as:

$$\hat{\beta_{i}}=\frac{\beta_{yi}+\beta_{xi}}{2}$$

Where $\beta_{xi}$ and $\beta_{yi}$are the methylation values for a given CpG for twin pair $i$ to the x- and y-axis, respectively. The mean absolute error (MAE) can be computed as:

$$MAE\left( \beta_{y},\hat{\beta} \right)=\frac{1}{n}\sum_{i=1}^{n} |\beta_{yi}-\hat{\beta_{i}}|=\frac{1}{n}\sum_{i=1}^{n} \frac{|\beta_{yi}-\beta_{xi}|}{2}=\frac{1}{n}\sum_{i=1}^{n} \frac{|{\Delta\beta}_{i}|}{2}=\frac{1}{2}\bar{|\Delta\beta|}=MAE\left( \beta_{x},\hat{\beta} \right)$$

Where $|{\Delta\beta}_{i}|$ is the absolute difference in methylation for a given CpG in twin pair $i$.

Such computed MAE can be interpreted geometrically as half the average Manhattan distance to the unit line across twin pairs points (Diagram 1).


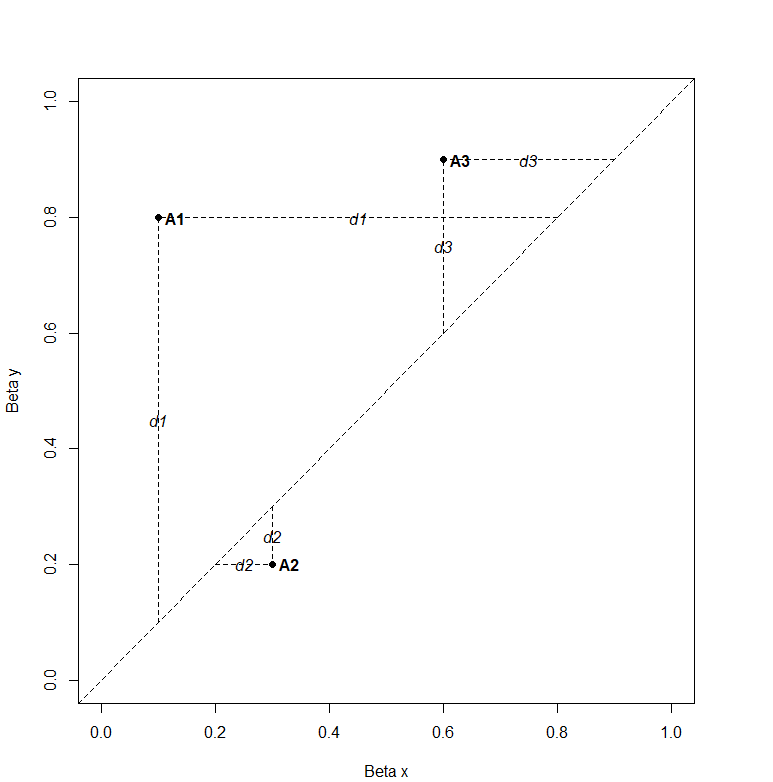


**Diagram 1**. Methylation for a given CpG across three twin pairs. Manhattan distances to the unit line (d1, d2 and d3) are highlighted as dashed lines. MAE is defined as the mean of these distances divided by two.

The maximum MAE that can be achieved is 0.5 which corresponds to twin pairs at ($\beta_{x1}$ = 0, $\beta_{y1}$ = 1) or ($\beta_{x1}$= 1, $\beta_{y1}$= 0). In order to ease interpretation, it is desirable that the designed measure for agreement between twins is bounded between 0 and 1. We propose concordance, $c$, defined as:

$$c= 1-\frac{{MAE}_{obs}}{{MAE}_{max}} c\in[0, 1]$$

Where ${MAE}_{obs}$ is the observed MAE for a given CpG in a twin pair set and ${MAE}_{max}$ is the maximum observable MAE. Although the case where twins show opposite methylation is the true maximum, it is unreasonable to employ it as a reference given its unrealistic nature. Instead we defined our reference as the more down-to-earth scenario where each twin’s methylation for a given CpG follows independent uniform distributions:

$$\beta_{y} \sim U\left( 0,1 \right); \beta_{x} \sim U\left( 0,1 \right)$$

We can then rewrite concordance as:

$$c= 1-\frac{{MAE}_{obs}}{{MAE}_{unif}}=1-\frac{\bar{|\Delta\beta|}_{obs}}{E[\left| {\Delta\beta}_{i} \right|_{unif}]}$$

It simply follows that:

$${{(\Delta\beta}_{i})}_{unif} \sim Tr\left( -1, 1, 0 \right); \left| {\Delta\beta}_{i} \right|_{unif} \sim Tr(0, 1, 0)$$

Where Tr corresponds to a triangular distribution with coefficients lower limit, upper limit and mode. For $\left| \Delta\beta\right|$, we can write the following probability density function:

$$\left| {\Delta\beta}_{i} \right|_{unif} \sim Tr(0, 1, 0)= \left\{ \begin{aligned} 0 if x<0 \\ \begin{matrix} 2-2x if 0\leq x\leq1 \\ 0 ifx>1 \end{matrix} \end{aligned} \right.$$

It is possible to analytically compute the following:

$$E[\left| {\Delta\beta}_{i} \right|_{unif}]=E\left[ Tr\left( 0, 1, 0 \right) \right]=\int_{0}^{1} x\cdot\left( 2-2x \right)\cdot dx=\left[ x^{2}-\frac{2}{3}x^{3} \right]_{0}^{1}=\frac{1}{3}$$

And hence:

$$c= 1-\frac{\bar{|\Delta\beta|}_{obs}}{E\left[ \left| {\Delta\beta}_{i} \right|_{unif} \right]}=1-3\cdot\bar{|\Delta\beta|}_{obs}$$

Although in theory, given that we do no longer normalize by the maximum MAE, the concordance coefficient is no longer restricted to the interval between [0, 1], in practice, no CpG approaches this limit as the case where MZ twins display opposite methylation values is completely unrealistic. For every CpG, concordance was computed as described above while range was obtained as:

$$range=\max\left( \hat{\beta} \right)-min(\hat{\beta})$$

Concordance-range plots were created with the hexbin R-package (**Fig 1D**, **Fig S8**).

**4. Assessing evCpGs’ measurement error in blood**

We here refer to measurement error as the variance between technical replicates. The technical variance in 450K signals can derive from many sources: DNA quantity and quality, bisulfite conversion, batch effects, data pre-processing, etc. all of which are expected to promote divergence between technical replicates. In order to assess measurement error, we initially checked for similarity in technical 450K measure in order to make sure that the pipeline had not deliberately enriched for low performance probes (**Fig S9**). We then extended the analysis to technical and longitudinal replicates.

**4.1 450K technical measures**

**4.1.1 Number of beads**

In the 450K array, there are three types of probes:

- Type II (targeted cytosine is not included in the probe; one sole oligonucleotide targets both epialleles; single-base extension fluorescence is informative on both red and green channels),
- Type I Green (targeted cytosine is included in the probe; there are two different oligonucleotides for the methylated and unmethylated allele; single-base extension fluorescence is informative in the green channel) and
- Type I Red (targeted cytosine is included in the probe; two different oligonucleotides for the methylated and unmethylated allele; single-base extension fluorescence is informative in the red channel).

Hundreds of thousands of such probe oligonucleotides cover 3 μm silica beads that self-assemble on a substrate interspaced by 5.7 μm. There are normally several beads representing each oligonucleotide pool (referred to as bead type), but the number is variable depending on the manufacture of the microarray itself. It has previously been noted that oligos represented by smaller number of beads give rise to higher measurement error (fluorescence is averaged over a smaller number of beads, hence larger variability) [24]. For example, a well-known 450K quality control function ENmix::QCinfo sets the default threshold of inappropriate number of beads per probe to 3: probes are flagged when at least 5% of the total samples display less than 3 beads. For type I probes concretely, ENmix::QCinfo takes into account only the probe with the smallest number of beads from the two probes per site. In summary, CpGs displaying low number of beads are expected to display higher measurement error.

**4.1.2 Detection p-value**

The detection p-value derives from inference testing under the following hypotheses contrast:

H_0_: Signal = background

H_1_: Signal > background

This is the same as weighing the degree of evidence on how different the fluorescence intensity signal is, compared to that of the background. A p-value > α indicates that we cannot rejected that the signal is equal to the background (e.g. probe failure). It has been shown before that probes that frequently display probe failure across different samples tend to show greater technical variability. Again, ENmix::QCinfo sets the default threshold of detection p-value to 0.000001: probes are flagged when at least 5% of the samples display detection p-values higher than 0.000001.

**4.1.3 Intra-class correlation coefficient**

As mentioned above, ICC measures the proportion of non-technical variance compared to the total variance. An ICC of zero indicates that 100% of the variance could be explained by technical variance. In the 450K array, probes displaying ICC close to zero are common. This is often driven by very limited inter-individual variation rather than displaying larger measurement error: in the absence of biological variation, 100% of the variance is measurement error [22, 25].

**4.2 Technical replicates and short-term longitudinal stability**

To begin with we visualized the differences between MZ twins and technical replicates in two different ways in the Danish twin cohort.

Firstly, we represented the data as arrows pointing from technical replicates to MZ twins represented as coordinates in the concordance-range plane (described in 3.2, **Fig S10A-B**). In case that the observed variation is beyond measurement error, the methylation range is expected to increase (Δx = Δrange > 0), while the concordance is expected to decrease (Δy = Δconcordance < 0).

Secondly, we visualized how the combination of all evCpGs is able to separate monozygotic twins via a heatmap performed with gplots::heatmap.2. We compared the resolving power of evCpGs with a set of negative control CpGs previously reported for strong genetic effects, which were not expected to resolve MZ twins. These derive via ranking reported blood mQTL CpGs by significance in adolescents from the ARIES cohort [26] and selecting a number equal to that of available evCpGs. We also performed this analysis on Group A from Zhang *et al.* data (*dataset-C1*), as it also included technical replicates of MZ twins (**Fig S11A**).

Finally, we performed statistical inference to assess whether the differences observed were larger in MZ twins than in technical replicates with a Kolmogorov-Smirnov test (**Fig S10C**).

Once proven that the differences that were observed are superior to measurement error, we asked whether such differences were stable in time or simply erratic. For that, we used the data from group B of Zhang *et al.* (*dataset-C2*). Again, with a heatmap and the use of mQTL-derived negative control CpGs we show that in spite of time of collection, individuals sampled at different time points cluster together. We also provide the temporal ICC distribution in the form of a violin plot, whose estimates had been previously published by Flanagan *et al* in [27] (**Fig S11B**).

**5. evCpGs’ epigenetic clock/drift in blood**

Like in a standard epigenome-wide association study (EWAS), we performed regression association testing for age. We employed the cpg.assoc function from the CpGassoc R-package [28] which, per CpG, fits a linear model where the dependent variable is the beta-value and the independent variable is age; we also included sex as a covariate. The input evCpG beta-value matrix employed was derived from *dataset-D* (**Fig S12A**) or from *dataset-E* (**Fig 3C-D**). Obtained age-regression coefficient p-values were corrected for multiple testing with Bonferroni correction (α/n = 0.05/331). Also, per CpG, we assessed whether heteroscedasticity with respect to age was present by carrying out White tests based on the lmtest::bptest function. Briefly, it first creates a linear model identical to the one employed for age association (sex included as a covariate). It then extracts the squared residuals and builds a second linear model or auxiliary model, also supplied with the gender covariate. From the R^2^ of the auxiliary model it is possible to compute a p-value for heteroscedasticity. We preferred this option to an ordinary Breusch-Pagan test, as it additionally includes a quadratic term for age in the auxiliary linear model. Heteroscedasticity p-values were also corrected for multiple testing via Bonferroni correction (α/n = 0.05/331).

**6. Behavior of evCpGs in other tissues**

**6.1 Post-mortem tissues**

To assess whether evCpG methylation is subject to between tissue variation, we made use of a large panel of post-mortem tissues (*dataset-H*); this way we can obtain a high number of tissues per individuals. We performed multi-dimensional scaling (MDS) plots with the minfi::mdsPlot function for the 65 450K SNP probes, available evCpGs and equal number of control CpGs known to display strong genetic effects extracted from mQTL from adolescents of the ARIES study [26] (**Fig S13**). To extract the percentage of variance explained, identically to what minfi::mdsPlot performs, we made use of the function cmscale on the matrix of Euclidean distances obtained from the evCpG/control CpG methylation data across individuals but this time with parameter eig = TRUE in order to obtain the eigenvalues of the distance matrix. The percentage of variance is simply equal to the highest eigenvalue (PC1) or the second highest eigenvalue (PC2) divided by the sum of all eigenvalues.

**6.2 Replication of evCpGs in adipose tissue**

We replicated the discovery of evCpGs in *dataset-I*. However, there are some cohort biases to be considered:

1. The sample size is much smaller than the dataset employed for discovery in blood.
2. Although the authors that made the dataset publicly available did not share age, we know that individuals within this cohort are generally quite old in contrast to 18 years old employed in whole blood. Hence, epigenetic drift effects are expected to be more predominant in this dataset
3. Only female MZ twins are included in this dataset.

Having identified possible biases and given that the data was already preprocessed, we had to re-establish an equivalence range for this normalization. For that, we excluded CpGs associated to X- and Y-chromosomes based on IlluminaHumanMethylation450kmanifest R-package. With respect to our SNP policy, we employed the SNPs.147CommonSingle file from R-package IlluminaHumanMethylation450kanno.ilmn12.hg19 which is based on dbSNP v.147. We excluded all probes containing SNPs at the CpG or in the single base extension site (the latter solely for type I probes) and probes containing SNPs with MAF > 0.01 within the probe. With respect to cross-reactive probes, we excluded all CpGs predicted *in silico* to be cross-hybridizing as reported by Chen *et al* and Price *et al* [18, 19]. We also excluded CpGs that displayed less than the suggested ICC empirical cut-off of 0.37 in blood [22]. Optimally, it would have been better to employ ICCs computed in adipose tissue, but no study addressed this question to this date to the best of our knowledge. We additionally excluded CpGs that displayed an inter-quantile range (IQR) of less than 0.07. IQRs were computed with the whole adipose tissue dataset (n = 648) to avoid selection biases.

We used the remaining 8,142 CpGs to define the parameter epsilon ($\varepsilon$), which characterizes the resolution at which the difference in two means can be defined as equivalent employing:

$$\varepsilon={Median}_{i}({Tr}_{j}({{|\Delta\beta|}_{ij}}^{MZ twins}))$$

Where${{|\Delta\beta|}_{ij}}^{MZ twins}$ is the matrix of absolute difference of methylation ($i$ CpGs as rows and $j$ samples as columns),${Tr}_{j}$ is the trimmed mean operator across$j$ samples and ${Median}_{i}$ is the median operator across $i$ CpGs. The epsilon parameter that was finally chosen was $\varepsilon_{adipose}=0.04196169$.

We then tested the evCpGs available in this dataset (332 out of the 333) for equivalence under the paradigm of equivalence testing with a two one-sided tests (TOST). As before, we based the TOST on Yuen t-test [23], employing the wrappers supplied by equivalence::rtost with parameters Trim = 0.2. Additionally, we tested short-term longitudinal stability in adipose tissue. As for longitudinal analysis in blood (section 4.2), we wondered whether such differences were stable in time for adipose tissue. For that, we used the data from [10]. Similarly to blood, we used a heatmap and the use of negative control CpGs. Additionally, we estimated temporal ICC’s employing the ICC::ICCest function (**Fig S14**). For this, we excluded the individual “WeightRegainers3” given that the sample was an outlier for both evCpGs and control CpGs. We finally represented the distribution of ICC in evCpGs and control CpGs with a violin plot.

**7. Functional annotation of evCpGs**

**7.1 Enrichment analysis**

We defined the following sets:

$$T\equiv Target CpG set;{Bg}_{0}\equiv Background CpG set$$

$$Bg=\left\{ x \in{Bg}_{0} \right| x\notin T\}$$

where $T$ consisted of evCpGs while ${Bg}_{0}$ consisted of all 450K probes except for low-quality, X- and Y-chromosome, SNP-containing and cross-reactive probes; probes with little to no variation (ICC < 0.37 and IQR < 0.07) were included. $Bg$ is defined as the set that contains the background and excludes $T$. As a result, $T$ and $Bg$ are mutually exclusive (required for Fisher’s exact test).

**7.1.1 Imprinted genes, metastable epialleles, EWAS traits and mQTL**

Known and predicted imprinted human genes were extracted from the Geneimprint database (<http://www.geneimprint.com/site/genes-by-species>), human metastable epiallele-like CpGs were extracted from [29], while EWAS-associated trait CpG annotation was obtained from [30]. We counted the number of instances belonging to a given category (imprinted gene/metastable epiallele/a given EWAS trait) in $Bg$ and in $T$ and built a 2×2 contingency table. Enrichment was performed via Fisher’s exact test. For EWAS traits (**Fig S20**), we only tested those traits that were present in $T$ (Bonferroni multiple testing correction was applied for a total number of 81 traits).

Also, we extracted mQTL discovered in blood of adolescents [26] from the mQTL database file, 15up.ALL.M.tab, a filter to only those that displayed p-values less than 1×10^-14^ (as per recommendation from the authors). We counted the number of CpGs with associated mQTL in $T$ (n = 333) and in a background, this time consisting of all non-significant variably methylated (n = 4,319) CpGs and built a 2×2 contingency table for which we examined for enrichment via Fisher’s exact test.

**7.1.2 Functional and CpG island status**

Based on the IlluminaHumanMethylation450kanno.ilmn12.hg19 file, we annotated CpGs. We counted the number of instances belonging to a given category (island status/ functional status) in $Bg$ and in $T$ and built a *m*×2 contingency table (m = 7 for functional status and m = 6 for island status). Significance for all categories was investigated on the m×2 contingency tables via Fisher’s exact test with Monte Carlo simulation for 100,000 permutations. Significance per category was tested by collapsing the absolute table of counts into 2×2 contingency table (belonging to a given category or not, for $Bg$ and $T$) and by performing a Fisher’s exact test on it (**Fig S17**). Bonferroni multiple testing correction was applied for the total number of categories tested for significance (respectively, 6 and 7 for CpG island and functional status). Specifically for functional status, CpGs were commonly associated to several classes; for this reason, we decided to employ the following hierarchical order to assign the highest priority class to a given CpG: 5’-UTR > TSS200 > TSS1500 > 1^st^ Exon > Body > 3’-UTR > Not gene associated.

**7.1.3 PBMC 15-states ChromHMM**

As part of Roadmap Epigenomics mapping consortium [31], a hidden markov model (HMM) was built based on data derived from primary mononuclear cells (PBMC) from peripheral blood by which the whole genome was segmented into 15 categories or states (ChromHMM): **(1)** TssA: active TSS promoter; **(2)** TssAFlnk: flanking active TSS promoter; **(3)** TxFlnk: transcribed state at the 5’ or 3’ end of genes with promoter and enhancer signatures; **(4)** Tx: actively transcribed; **(5)** TxWk: weakly transcribed; **(6)** EnhG: genic enhancer; **(7)** Enh: enhancer states; (**8)** ZNF/Rpts: associated with zinc finger genes; **(9)** Het: constitutive heterochromatin; **(10)** TssBiv: bivalent/poised TSS; **(11)** BivFlnk: flanking bivalent TSS/Enhancer; **(12)** EnhBiv: bivalent enhancer; **(13)** ReprPC: repressed polycomb; **(14)** ReprPCWk: weak repressed Polycomb; **(15)** Quies: quiescent.

The data was obtained from ENCODE (accession: ENCSR550VPH) in bigBed format which was converted to a Bed format file with the BigBedToBed tool obtained from UCSC server (<http://hgdownload.soe.ucsc.edu/admin/exe/>). We subsequently annotated all probes in the 450K with its respective category. We then counted the number of instances belonging to a given ChromHMM state in $Bg$ and in $T$ and built a *15*×2 contingency table. Significance for all categories was investigated via Fisher’s exact test with Monte Carlo simulation for 100,000 permutations. Significance per category was tested by collapsing the absolute table of counts into 2×2 contingency table (belonging to a given category or not, for $Bg$ and $T$) and by performing a Fisher’s exact test on it (**Fig S18**). Bonferroni multiple testing correction was applied for the total number of categories tested for significance (α/15).

**7.1.4 Gene ontology terms**

Gene Ontology (GO) term enrichment was performed with the missMethyl::gometh function which was supplied with $T$ and ${Bg}_{0}$, with arguments collection = “GO”, array.type = “450K” and prior.prob = T. The last argument ensured that the probability of false discovery due to a high number of probes per gene was taken into account.

**7.1.5 DNA motif and [G+C] content**

We extracted 500 bp up- and down-stream the CpG sites for $T$ and the ${Bg}_{0}$by feeding a file with the coordinates of choice (evCpGs500 and bg500) into samtools (v1.9) [32]. The following calls were employed:

*samtools faidx hg19.fasta.gz –r evCpGs500 > evCpGs500.fa*

*samtools faidx hg19.fasta.gz –r bg500 > bg500.fa*

We validated the number of entries with:

*tr -cd ‘>’ < evCpGs500.fa | wc -c*

*tr -cd ‘>’ < bg500.fa | wc -c*

We finally run Homer (v4.10) [33] for known and *de novo* motif enrichment analysis with the following parameters (**Fig S15**, **Fig S16A**):

*findMotifs.pl evCpGs500.fa fasta . -fasta bg500.fa -p 4 -humanGO > log.txt*

On another note, $T$ and the ${Bg}_{0}$ fasta files were input into R with the help of the seqinr R-package [34] for which we computed [G+C] content for all sequences. Finally, we tested whether the [G+C] content in the target was diminished compared to background via a Mann-Whitney U-test (**Fig S16B**).

**7.1.6 Genomic Position**

Positional enrichment analysis allows to check whether probes are nearby, while taking into account that some genomic regions are better covered by the 450K design than others. For every element of $T$, a window of window length $l$ was centered around the CpG of interest. Then, the number of CpGs that fall within and outside the window was counted for the $T$ and $Bg$. Finally, a 2x2 contingency table was built and a Fisher’s exact test was deployed to test for enrichment. Obtained p-values were corrected for multiple testing with Bonferroni correction (α/n = 0.05/333).

**7.2 RNA expression of evCpG-associated genes**

Median expression levels for 247 out of the 264 evCpG-associated genes were extracted from the Genotype Tissue Expression (GTEx) portal (<https://gtexportal.org/home/datasets>) by the name: GTEx_Analysis_2016-01-15_v7_RNASeQCv1.1.8_gene_median_tpm.gct and visualized in a heatmap (**Fig S19**).

**8. Whole-genome bisulfite sequencing analysis**

Unfiltered processed whole-genome bisulfite sequencing (WGBS) data derived from whole blood belonging to MZ twins were obtained from the ArrayExpress database with accession E-MTAB-3549 (*datasets-K1,K2*). Similar to [11], we excluded sites with more than 20% methylation differences between strands or that fell within the Duke Excluded Regions (<https://www.encodeproject.org/annotations/ENCSR797MUY/>) or the DAC Blacklisted Regions (<https://www.encodeproject.org/annotations/ENCSR636HFF/>), known to yield artefactual high coverage. We additionally applied both high- and low-end coverage filters. We excluded: i) sites with coverage less or equal to 10 reads and ii) larger than the per-sample 99.9% quantile. Altogether, this procedure improves the accuracy of the methylation estimates per site and filters out possible PCR artifacts at the high end of the coverage. Per twin pair, we then selected only those sites that were common.

As we wanted to compare WGBS data with 450K data, we were interested on relying on positions rather than regions. However, in order to separate real variation from technical one, depth per site must be considered in the analysis as twin differences may arise simply by sampling error. We ran some simulations to choose a threshold for (absolute value of delta beta) above which twin methylation differences can be considered improbable to have been arisen via random sampling. We simulated a CpG with beta-value of 0.5 (maximum variance), for which we sampled, as our low coverage filter, 10 read values (either methylated, 1, or unmethylated, 0) using a Bin(10,0.5) distribution for 10,000 times obtaining the sampling $\beta$ distribution. We then obtain the sampling $\left| \Delta\beta\right|$ distribution by computing all possible combinations of absolute value differences from this set (a total of 49,995,000 combinations). We established our threshold criteria to be the 95% quantile of the sampling$\left| \Delta\beta\right|$ distribution which corresponded to 0.4. Differences higher or equal to this threshold are very unlikely to have arisen from random sampling only. Finally, positional enrichment analysis was performed on the *cPCDH* region (chr5:140165876:140892546 for genome assembly hg19). Per twin, we computed the number of sites with |Δβ|^twin^ ≥ 0.4 and |Δβ|^twin^ < 0.4 within and outside this region and performed a Fisher’s exact test to obtain an enrichment p-value (**Fig S21-23**).

**References**

1. Hannon E, Knox O, Sugden K, Burrage J, Wong CCY, Belsky DW, et al. Characterizing genetic and environmental influences on variable DNA methylation using monozygotic and dizygotic twins. PLoS Genet. 2018; 14:e1007544; doi:10.1371/journal.pgen.1007544.

2. Tan Q, Frost M, Heijmans BT, von Bornemann Hjelmborg J, Tobi EW, Christensen K, et al. Epigenetic signature of birth weight discordance in adult twins. BMC Genomics. 2014 15; doi:10.1186/1471-2164-15-1062.

3. Zhang N, Zhao S, Zhang SH, Chen J, Lu D, Shen M, et al. Intra-Monozygotic Twin Pair Discordance and Longitudinal Variation of Whole-Genome Scale DNA Methylation in Adults. PLoS One. 2015; 10:e0135022; doi:10.1371/journal.pone.0135022.

4. Shi L, Jiang F, Ouyang F, Zhang J, Wang Z, Shen X. DNA methylation markers in combination with skeletal and dental ages to improve age estimation in children. Forensic Sci Int Genet. 2018; 33:1-9; doi:10.1016/j.fsigen.2017.11.005.

5. Johansson A, Enroth S, Gyllensten U. Continuous Aging of the Human DNA Methylome Throughout the Human Lifespan. PLoS One. 2013; 8:e67378; doi:10.1371/journal.pone.0067378.

6. Kurushima Y, Tsai PC, Castillo-Fernandez J, Couto Alves A, El-Sayed Moustafa JS, Le Roy C, et al. Epigenetic findings in periodontitis in UK twins: a cross-sectional study. Clin Epigenetics. 2019 11; doi:10.1186/s13148-019-0614-4.

7. Van Baak TE, Coarfa C, Dugue PA, Fiorito G, Laritsky E, Baker MS, et al. Epigenetic supersimilarity of monozygotic twin pairs. Genome Biol. 2018; 19:2; doi:10.1186/s13059-017-1374-0.

8. Lokk K, Modhukur V, Rajashekar B, Märtens K, Mägi R, Kolde R, et al. DNA methylome profiling of human tissues identifies global and tissue-specific methylation patterns. Genome Biol. 2014 15; doi:10.1186/gb-2014-15-4-r54.

9. Grundberg E, Meduri E, Sandling JK, Hedman AK, Keildson S, Buil A, et al. Global analysis of DNA methylation variation in adipose tissue from twins reveals links to disease-associated variants in distal regulatory elements. Am J Hum Genet. 2013 93; doi:10.1016/j.ajhg.2013.10.004.

10. Bollepalli S, Kaye S, Heinonen S, Kaprio J, Rissanen A, Virtanen KA, et al. Subcutaneous adipose tissue gene expression and DNA methylation respond to both short- and long-term weight loss. Int J Obes (Lond). 2018; 42:412-423; doi:10.1038/ijo.2017.245.

11. Busche S, Shao X, Caron M, Kwan T, Allum F, Cheung WA, et al. Population whole-genome bisulfite sequencing across two tissues highlights the environment as the principal source of human methylome variation. Genome Biol. 2015; 16:290; doi:10.1186/s13059-015-0856-1.

12. R Development Core Team: R: A Language and Environment for Statistical Computing**.** Vienna, Austria; 2017.

13. Aryee MJ, Jaffe AE, Corrada-Bravo H, Ladd-Acosta C, Feinberg AP, Hansen KD, et al. Minfi: a flexible and comprehensive Bioconductor package for the analysis of Infinium DNA methylation microarrays. Bioinformatics. 2014; 30:1363-1369; doi:10.1093/bioinformatics/btu049.

14. Xu Z, Niu L, Li L, Taylor JA. ENmix: a novel background correction method for Illumina HumanMethylation450 BeadChip. Nucleic Acids Res. 2016; 44:e20; doi:10.1093/nar/gkv907.

15. Pidsley R, Y. Wong CC, Volta M, Lunnon K, Mill J, Schalkwyk LC. A data-driven approach to preprocessing Illumina 450K methylation array data. BMC Genomics. 2013 14; doi:10.1186/1471-2164-14-293.

16. Phipson B, Maksimovic J, Oshlack A. missMethyl: an R package for analyzing data from Illumina's HumanMethylation450 platform. Bioinformatics. 2016; 32; doi:10.1093/bioinformatics/btv560.

17. Davis S, Meltzer PS. GEOquery: a bridge between the Gene Expression Omnibus (GEO) and BioConductor. Bioinformatics. 2007; 23:1846-1847; doi:10.1093/bioinformatics/btm254.

18. Chen YA, Lemire M, Choufani S, Butcher DT, Grafodatskaya D, Zanke BW, et al. Discovery of cross-reactive probes and polymorphic CpGs in the Illumina Infinium HumanMethylation450 microarray. Epigenetics. 2013; 8:203-209; doi:10.4161/epi.23470.

19. Price ME, Cotton AM, Lam LL, Farré P, Emberly E, Brown CJ, et al. Additional annotation enhances potential for biologically-relevant analysis of the Illumina Infinium HumanMethylation450 BeadChip array. Epigenetics Chromatin. 2013; 6; doi:10.1186/1756-8935-6-4.

20. Reinius LE, Acevedo N, Joerink M, Pershagen G, Dahlen SE, Greco D, et al. Differential DNA methylation in purified human blood cells: implications for cell lineage and studies on disease susceptibility. PLoS One. 2012; 7:e41361; doi:10.1371/journal.pone.0041361.

21. Houseman EA, Accomando WP, Koestler DC, Christensen BC, Marsit CJ, Nelson HH, et al. DNA methylation arrays as surrogate measures of cell mixture distribution. BMC Bioinformatics. 2012 13; doi:10.1186/1471-2105-13-86.

22. Bose M, Wu C, Pankow JS, Demerath EW, Bressler J, Fornage M, et al. Evaluation of microarray-based DNA methylation measurement using technical replicates: the Atherosclerosis Risk In Communities (ARIC) Study. BMC Bioinformatics. 2014; 15; doi:10.1186/1471-2105-15-312.

23. Yuen KK. The two-sample trimmed t for unequal population variances. Biometrika. 1974; 61; doi:doi.org/10.1093/biomet/61.1.165.

24. Logue MW, Smith AK, Wolf EJ, Maniates H, Stone A, Schichman SA, et al. The correlation of methylation levels measured using Illumina 450K and EPIC BeadChips in blood samples. Epigenomics 2017 9; doi:10.2217/epi-2017-0078.

25. Forest M, O'Donnell KJ, Voisin G, Gaudreau H, MacIsaac JL, McEwen LM, et al. Agreement in DNA methylation levels from the Illumina 450K array across batches, tissues, and time. Epigenetics. 2018; 13:19-32; doi:10.1080/15592294.2017.1411443.

26. Gaunt TR, Shihab HA, Hemani G, Min JL, Woodward G, Lyttleton O, et al. Systematic identification of genetic influences on methylation across the human life course. Genome Biol. 2016; 17:61; doi:10.1186/s13059-016-0926-z.

27. Flanagan JM, Brook MN, Orr N, Tomczyk K, Coulson P, Fletcher O, et al. Temporal stability and determinants of white blood cell DNA methylation in the breakthrough generations study. Cancer Epidemiol Biomarkers Prev. 2015; 24:221-229; doi:10.1158/1055-9965.EPI-14-0767.

28. Barfield RT, Kilaru V, Smith AK, Conneely KN. CpGassoc: an R function for analysis of DNA methylation microarray data. Bioinformatics. 2012; 28:1280-1281; doi:10.1093/bioinformatics/bts124.

29. Harris RA, Nagy-Szakal D, Kellermayer R. Human metastable epiallele candidates link to common disorders. Epigenetics. 2013; 8:157-163; doi:10.4161/epi.23438.

30. Li M, Zou D, Li Z, Gao R, Sang J, Zhang Y, et al. EWAS Atlas: a curated knowledgebase of epigenome-wide association studies. Nucleic Acids Res. 2019; 47:D983-D988; doi:10.1093/nar/gky1027.

31. Roadmap Epigenomics C, Kundaje A, Meuleman W, Ernst J, Bilenky M, Yen A, et al. Integrative analysis of 111 reference human epigenomes. Nature. 2015; 518:317-330; doi:10.1038/nature14248.

32. Li H, Handsaker B, Wysoker A, Fennell T, Ruan J, Homer N, et al. The Sequence Alignment/Map format and SAMtools. Bioinformatics. 2009; 25:2078-2079; doi:10.1093/bioinformatics/btp352.

33. Heinz S, Benner C, Spann N, Bertolino E, Lin YC, Laslo P, et al. Simple combinations of lineage-determining transcription factors prime cis-regulatory elements required for macrophage and B cell identities. Mol Cell. 2010; 38:576-589; doi:10.1016/j.molcel.2010.05.004.

34. Charif D, Lobry JR: SeqinR 1.0-2: a contributed package to the R project for statistical computing devoted to biological sequences retrieval and analysis**.** In *Structural approaches to sequence evolution: Molecules, networks, populations.* Edited by Bastolla U, Porto M, Roman HE, Vendruscolo M. In Bastolla: Springer Verlag; 2007: 207-232: *Biological and Medical Physics, Biomedical Engineering*].
