# Supplementary material for: Equivalent DNA methylation variation between monozygotic co-twins and unrelated individuals reveals universal epigenetic inter-individual dissimilarity
Source: Genome Biol. 2021 Jan 5;22:18. doi: 10.1186/s13059-020-02223-9 (PMC7786996; doi:10.1186/s13059-020-02223-9)
Supplement: Supplementary file 4 — Additional file 4. Review history. [file 13059_2020_2223_MOESM4_ESM.docx]

Review History

**First round of review**

**Reviewer 1**

**Are you able to assess all statistics in the manuscript, including the appropriateness of statistical tests used?**

Yes, and I have assessed the statistics in my report.

**Comments to author:**

Using a large published Illumina HM450 data set on MZ twin pairs, the authors set out to look for human genomic CpGs that show substantial interindividual variation among MZ co-twins. They claim (Abstract) to identify 333 CpG sites (evCpGs) showing similar degree of variation within MZ co-twins and unrelated individuals, and that "systematic" evCpG methylation in children supports establishment during early embryogenesis. (I think they intend to mean "systemic"; the "Universal" in the title also seems to imply systemic interindividual variation at these loci.) To lend biological import to their findings, the authors report that evCpGs are enriched at protocadherin gene clusters in the human genome. The existence of interindividual epigenetic differences among MZ co-twins is of long-standing interest in the field of epigenetics and human disease, as such differences could be a basis for phenotypic differences between isogenic humans. However, many conceptual and methodological shortcomings of the manuscript leave me unconvinced of the authors' findings.

Major concerns

The authors refer to data on beads per probe, high detection p value, and ICC as evidence that evCpGs are not simply the result of technical noise. It would be helpful if they articulate how these measures argue against the variants resulting from technical error. Also, "technical noise" is vague. Do the authors mean random technical error (as opposed to systematic errors)?

The data in figure S10 are not convincing. In particular, S10C shows that the twin-twin discordance at evCpGs is just barely greater than technical replicates. For 50% of evCpGs, twin-twin deltaBeta (i.e. discordance) is comparable to that of technical replicates! For 50% of evCpGs, twin-twin deltaBeta is less that 0.02. This does not validate the results in the original analysis. This is not even the appropriate comparision. MZ twin discordance should be compared to that of unrelated individuals, as in the original analysis (this could perhaps be simulated by shuffling the pair information in technical replicates).

Regarding the Gambian data set (line 111), since this is not a twin cohort, it is unclear what point the authors are trying to make.

The authors state (line 114) that their observations lead to the conclusion that evCpG stochastic variation is established early in embryonic development. It is entirely unclear how they reached this interpretation. In particular, it seems they have only considered data from peripheral blood. So 1) there is question of whether these variants might simply reflect interindividual variation in leukocyte composition, and 2) the question of whether these variants are systemic (i.e. consistent across different tissues and cell types).

The authors report from their GO analysis (line 119) that evCpGs are enriched for neurodevelopmental processes. This raises two issues. First, the background set used in in their analyses. Rather than reflecting all HM450 probes, it is apparent from their data that all evCpGs are derived from the small subset of HM450 probes showing interindividual variation of at least 0.2 Beta. Hence, this is the appropriate background set. Also, as described by Gunasekara et al (Genome Biol 2019), there is a tendency for genes expressed in neurons to be large genes. Simply because of this, GO analysis of randomly selected probes will be biased toward large genes (just by chance) and enriched for neurodevelopmental processes. The authors should adjust their GO analyses for gene size.

Regarding the enrichment for protocadherin (Pcdh) genes (line 147): Pcdh genes tend to be extremely long, so the same comments re: correcting for gene length are salient.

Regarding the external validation experiments (line 157) the authors should explain more clearly why data on only 2 of 7 MZ pairs was included. Otherwise this approach suggests cherry picking. Also, the Busche et al paper provides WGBS data on adipose tissue from the same individuals. Why not analyze data from adipose tissue as well? This would provide some information regarding the systemic nature of svCpGs, as well as the potential for svCpGs to simply reflect interindividual variation in leukocyte composition.

Minor concerns

The authors refer several times to "systematic" epigenetic variation. From context, it seems they mean "systemic". If not, I'm not sure what they mean by systematic epigenetic variation.

The authors cell type and tissue type as a driver of "systematic epigenetic variation". If they mean systemic, this does not make sense, as systemic variants are, by definition, not tissue specific.

The authors refer to "in utero stochastic mechanisms promoting epigenetic variation" (line 60). However, the processes they refer to occur prior to implantation, so "in utero" is inaccurate.

The authors refer to the advantages of studying twins of young age (line 73), but employ a data set on 18 year-old twins. Would not a data set on infant MZ twins be better? This merits comment.

I could not find any definition of what "evCpG" stands for.

The authors conclude (line 134) that "evCpGs avoid CpGs posing tight functional regulation in blood". It is not clear what this means, or how they arrived at this conclusion.

**Reviewer 2**

**Are you able to assess all statistics in the manuscript, including the appropriateness of statistical tests used?**

Yes, and I have assessed the statistics in my report.

**Comments to author:**

In this study, the authors aimed to identify CpGs that show similar large methylation variation among MZ co-twins and unrelated individuals, not caused by technical noise. Using a consecutive filtering in a discovery dataset of 426 MZ twin pairs they identified 333 CpGs representing true epigenetic variants (evCpGs) and validated those in independent datasets showing that such CpGs occur independent of genetic variation and /technical) measurement error. The authors conclude that these so-called evCpGs demarcate true stable individual epigenetic variants arising during early development which in the case of MZ twins might also serve as biomarkers for epigenetic fingerprinting (e.g., to discriminate between MZ twins in forensics). Oversall this is an important and well conducted analysis and the manuscript is well structured/written. I do have some (minor) concerns/suggestions that should be considered/addressed:

Abstract

-- The authors identified CpGs "displaying similarly large variation among co-twins and unrelated individuals surpassing measurement error", which they call "evCpGs". However, it is not clear why the authors use the term evCpGs. Where is "ev" referring to? This needs a clearer explanation (also in the Introduction).

-- The authors could also mention in the abstract that these "evCpGs" might serve as biomarkers for epigenetic fingerprinting, as they have the potential to discriminate between MZ co-twins.

(Supplementary) Methods

-- Authors should mention in the Methods section of the main manuscript, that the Methods are described in detail in the Supplementary Methods.

-- Page 4, Supplementary Methods "Exclusion of 450K putative noisy probes": Include for each filtering step described in this section the exact number of excluded probes.

-- Page 4, Supplementary Methods "450K data preprocessing": Three different methods were selected to normalize the array data (i.e., dasen, stratified quantile normalization, and oob_RELIC_QN_BMIQ). Since at least a dozen of different methods are available to normalize 450K data (Wang et al., Epigenetics, 2015; Shiah et al., Bioinformatics, 2017), the authors should explain why they selected these three methods.

-- Page 6, Supplementary Methods "Exclusion of 450K non-variable probes":

- Explain the intra-class correlation (ICC) coefficient in more detail, because it is now not clear how this coefficient is calculated and why it can be used to exclude non-variable probes.

- Also in this paragraph, the exact number of excluded probes should be mentioned for each filtering step.

-- Page 7, Supplementary Methods "evCpG discorvery": The paragraph starts with "For a total of 4,652 CpGs" …. Does this mean that after filtering only 4,652 CpGs were left from the 480,000? This should be explained.

-- For reproducibility reasons, these 4,652 CpGs should be listed in a Supplementary Table.

Results and Discussion

- Page 4, line 83: Since not all readers might be familiar with the 450K array, the authors should mention that the 450K array covers ~480,000 CpGs sites.

- Page 4, line 85-88: The authors should mention here that after strict quality control, prepossessing and exclusion of putative noisy and non-variable probes, 4,652 CpGs were left for the evCpG discovery analysis.

- Page 5, line 98: Please include after the ICC the reference to Bose et al. (BMC Bioinformatics, 2014), that describes the ICC.

- Page 5, line 99: Add here the total number of MZ twin pairs of dataset-B from the Danish Twin Registry. Please also add the number of samples (or MZ twin pairs) when you introduce dataset-C, dataset-D and dataset-E in the next paragraph.

- Page 5, line 108-110: Are the observed larger differences in the independent older twin cohort (dataset-D) indeed due to the older age, or could this also be due to a batch effect? Because Figure S1 shows that the "percent of low quality data per sample" is overall higher in dataset-D compared to dataset-A. The authors should also perform this test with dataset-B, because these twins are also older (mean age = 48.4 years) and the "percent of low quality data per sample" seems to be the highest in this dataset.

- Page 6, line 122: Explain the abbreviation ME in "ME-like CpGs".

- Page 7: The evCpGs were enriched at the protocadherin (PCDH) gene cluster at chromosome 5. Large DNA variation within MZ twins at the PCDH cluster has also recently been observed by Souren et al. (Nature Communications, 2019). The authors apparently missed that and should compare their data to this study, particularly discussing the fact that depp sequencing validation showed that some of such variants may be due to technical "artefacts" created on the Illumina arrays.

Figures

- Figure S1: Please standardize the scales of the x and y-axis of these 5 plots, so that the reader is able to compare them. Also increase the font of the labels.

- Figure S3 and S4: Please also standardize the scales of the y-axis of these plots, so that the reader can easily compare the figures and observe that the |Δβ| distributions differ between the used normalization methods.

- Figure S6: Standardize the y-axis of the three plots.

- Figure S8: Standardize the y-axis of the two plots.

- Figure S9B: The y-axis of figure 9B states "Proportion of samples with detection p-val >0.0001", but the legend states "b. Number of low-quality samples per CpG among significant (evCpGs) and non-significant hits". Please correct this mistake.

**Reviewer 3**

**Are you able to assess all statistics in the manuscript, including the appropriateness of statistical tests used?**

Yes, and I have assessed the statistics in my report.

**Comments to author:**

The manuscript by Vidaki and colleagues touches on an interesting, yet vastly understudied biological phenomena, that being interindividual epigenetic variation.

In human population epigenetics, epigenome-wide association studies and various measures of epigenetic age are commonplace, yet the community is only now beginning to realize that changes in variability might constitute yet another measure by which the epigenome (or more specifically, the DNA methylome) is linked to different environmental exposures and/or health outcomes. As such, in principle, the principal idea behind this manuscript is very innovative and timely and I was very excited that this topic was tackled in a systematic way. Specifically, the authors set out to use blood DNAm data from a large cohort of adolescent monozygotic twins to determine 333 CpGs that show a large variation among twins beyond technical noise (they call these evCpGs). They confirm in a number of publically available cohort data that these evCpGs also are highly variable in the blood of unrelated individuals, and identify one particular genomic locus (the protocadherin one) that appears to be particularly susceptible to such variation. For reasons not entirely clear to me, the authors then conclude that the variation at evCpGs is established during embryogenesis rather than resulting from postnatal changes in the sculpting of the DNA methylome.

My initial excitement was greatly dampened upon repeated reading of the manuscript, as in my opinion, it unfortunately falls short on its premise for a number of reasons. As outlined in more detail below, I have three partly overlapping primary concerns that - if addressed properly - will make this a stronger manuscript that might be of interest for a broad readership. Collectively, I feel that some of the conclusions drawn are not entirely supported by the data, or at least leave room for other interpretations. I don't think that this is due to their statistical approach, which as far as I can tell is solid and appropriate for the questions asked.

First, I have concerns as to whether the existing analyses, and the cohorts being used, are appropriate to support the kind of conclusions that the authors are drawing. I note that the authors did not use a single cohort with repeated measures and yet try to make statements about epigenetic drift/variation in a very developmental context. I do appreciate that they use a cohort of much younger children from Gambia in part to address this perspective but I am a bit perplexed that they did not utilize cohorts that allow them to more rigorously test this idea. In my mind, a longitudinal measure of DNAm in twins starting at birth would dramatically strengthen this work. The key question would be the extent to which evCpGs already are present at birth, which would be consistent with the authors' assertion that they are likely arise during embryogenesis. I am aware of at least one such dataset that could be utilized here. Martino et al Genome Biology 2013 (PMID: 23697701) performed a longitudinal, genome-scale analysis of DNA methylation in twins from birth to 18 months of age that revealed rapid epigenetic change in early life and pair-specific effects of discordance. While this work was done in buccal epithelial cells, I nonetheless have some difficulties reconciling its messages with the ones presented by Vidaki and colleagues. Further, I was surprised that as far as I can tell, this work was not even referenced here. Analyzing their evCpGs in the Marino data would also allow Vidaki and colleagues to examine more broadly as to whether the conclusions they are drawing from blood hold true for at least one other tissue commonly used in epigenetic studies. This is interesting from a biological perspective and would also make this work more broadly applicable. Beyond longitudinal data in twins, I was also surprised that the authors did not carefully examine the developmental dynamics of their evCpGs in ALSPAC, as that cohort has three different DNAm measures from birth to adolescence.

Second, I feel that the work as is, and as I read and understand it, falls short of putting epigenetic variation in the context of the emerging literature, and fails to draw connections to the existing data. Beyond the obvious emission of the Marino work mentioned before, there are a couple of directly relevant studies. For example, while small (10 twin pairs), Zhang et al PLoS One 2015 (PMID: 26248206) might be useful to integrate as it provides 4 DNAm from the same individuals over the course of a year, thus allowing short term assessment of the fluctuations of the evCpGs identified here within a person/twin pair. I would be reassured about the stability of the evCpGs if they showed very high ICC's across the timepoints measured here. I also would strongly encourage the authors' to put their work in the context of Li et al Int Journal Epidemiology PMID: 29518222), which looked at average DNAm across the lifespan in a number of cohorts, including several twin studies.

Beyond these directly relevant studies, I am having a hard time reconciling the messages from Vidaki and colleagues with existing studies of DNAm variation in human populations. In part this might be due to definitions and statistical approaches, but I do note that 333 variable CpGs out of 450,000 that are being measured is a very small number. I do realize that the discovery of such CpGs in monozygotic twins is a unique strength, but nonetheless find it hard to compare this to existing data that looked at epigenetic variation, across individuals, both as it relates to the nature of the variable CpGs (beyond the developmental context mentioned before) but also the pure number of such loci. Work in non-twin cohorts also has suggested that variable CpGs might not only be tissue-specific, but also enriched for being under genetic control. At the very least it would be very interesting to examine how their evCpGs compare to variable CpGs identified in community cohorts of children (for example Islam et al Epigenetics and Chromatin 2019, PMID: 30602389) or in aging human populations (for example McEwen et al Epigenetics and Chromatin 2015, PMID: 28465725).

Third, as written, overall I found the work as presented rather inaccessible except perhaps for the most expert reader. I think there are at least two reasons for this, including the required brevity of a Short Report in Genome Biology but also some apparent confusion about definitions. Specifically, I found it hard to discern what the authors meant with epigenetic drift versus interindividual variation, and whether they considered them distinct, similar or partly overlapping. In fact, some of the concerns I noted earlier might be rooted in my confusion about how excatly the authors used these concepts. To bring more clarity to this important framing issue, I would encourage the authors to at least consider the definition of epigenetic drift put forward by Jones et al Aging Cell 2015 (PMID: 25913071) as I believe it might help to conceptualize what the authors are actually measuring in a more accessible fashion and possibly contrast it from "general" epigenetic variation.

**Authors Response**

**Point-by-point responses to the reviewers’ comments:**

Responses to reviewers’ comments

Reviewer 1

Using a large published Illumina HM450 data set on MZ twin pairs, the authors set out to look for human genomic CpGs that show substantial interindividual variation among MZ co-twins. They claim (Abstract) to identify 333 CpG sites (evCpGs) showing similar degree of variation within MZ co-twins and unrelated individuals, and that "systematic" evCpG methylation in children supports establishment during early embryogenesis. (I think they intend to mean "systemic"; the "Universal" in the title also seems to imply systemic interindividual variation at these loci.) To lend biological import to their findings, the authors report that evCpGs are enriched at protocadherin gene clusters in the human genome. The existence of interindividual epigenetic differences among MZ co-twins is of long-standing interest in the field of epigenetics and human disease, as such differences could be a basis for phenotypic differences between isogenic humans. However, many conceptual and methodological shortcomings of the manuscript leave me unconvinced of the authors' findings.

Major concerns

1. The authors refer to data on beads per probe, high detection p value, and ICC as evidence that evCpGs are not simply the result of technical noise. It would be helpful if they articulate how these measures argue against the variants resulting from technical error.

*Reply: Given the briefness of the initial short report format, we acknowledge that we did not include such discussion in the manuscript. With the format upgrade to full article as allowed by the editor, we have now included a short summary on these measures in the revised manuscript’s methods (lines 470-476) as well as an extensive explanation in the Supplementary methods (lines 431-485). In brief, the 450K oligos designed to target bisulfite-converted genomic loci are displayed on beads. However, depending on the manufacture of the array, a variable number of beads represent each locus. Generally, oligos represented in a smaller number of beads result in higher technical noise. Secondly, the detection p-value weighs the degree of evidence on how different the fluorescence intensity signal is, compared to that of the background. Therefore, a high detection p-value is indicative of probe failure. A probe that frequently fails can also lead to higher technical error. Lastly, the intra-class correlation coefficient (ICC) measures the proportion of non-technical variance to total variance; therefore, an ICC of zero indicates that 100% of the variance could be explained by technical variance.*

*Given that CpGs displaying only technical error are also expected to show equivalent co-twin and unrelated inter-individual variation, we ought to provide evidence in support for evCpGs displaying greater methylation variation than measurement error. The aforementioned paragraph aimed to explain that the pipeline was not just deliberately enriching for CpGs displaying lower technical properties. The success of our filtering steps is underlined by proving similar distributions in ICC, number of beads and proportion of samples with detection p-values > 1·E-06 between evCpGs and non-significant CpGs.*

2. Also, "technical noise" is vague. Do the authors mean random technical error (as opposed to systematic errors)?

*Reply: In our study, we referred to technical noise as the variance observed between technical replicates. This may seem unspecific, but we chose this term deliberately due to the wide range of factors that can promote variance between technical replicates on the 450K array. Pursuing clarity, the term has now been replaced for ‘measurement error’ in the manuscript. A definition and thorough explanation have been added in the revised Supplementary methods as well (lines 431-439).*

3. The data in figure S10 are not convincing. In particular, S10C shows that the twin-twin discordance at evCpGs is just barely greater than technical replicates. For 50% of evCpGs, twin-twin deltaBeta (i.e. discordance) is comparable to that of technical replicates! For 50% of evCpGs, twin-twin deltaBeta is less that 0.02. This does not validate the results in the original analysis. This is not even the appropriate comparision. MZ twin discordance should be compared to that of unrelated individuals, as in the original analysis (this could perhaps be simulated by shuffling the pair information in technical replicates).

*Reply: We believe that the reviewer has misinterpreted the purpose and data analysis on Fig S10C, probably due to the compactness of the previously used short report format that forced us to skip valuable descriptions. We hereby provide a brief explanation that aims to clarify this point and a more thorough explanation has now been included in the revised manuscript (lines 150-161). In the discovery stage, we set to identify CpG sites where |Δβ|MZ twins is equivalent to |Δβ|unrelated individuals. Studying measurement error in evCpGs by no means pursues to replicate the discovery. Instead, we set an addition to the discovery hypothesis: |Δβ|MZ twins is equivalent to |Δβ|unrelated individuals but higher than |Δβ|technical replicates. This is of uttermost importance given that CpGs displaying only technical noise (ICC of zero) are expected to display |Δβ|MZ twins equivalent to |Δβ|unrelated individuals. Also, we would like to stretch the fact that proving that evCpG variance is not measurement error is an insurmountable challenge given that we do not dispose of a phenotype for which association can be measured on. The differences we expect in our hypothesis are of a ‘stochastic’ nature, unlike EWAS phenotypes for which association can be statistically tested. This issue is common to any epigenome-study pursuing to characterize stochastic methylation variation. We tried to avoid CpG sites enriched in technical noise by initially filtering out CpGs displaying small to no methylation variation (IQRβ-value < 0.07 and ICC < 0.37). This section of the paper (lines 150-166) is dedicated to evaluating whether the filtering strategy was successful. In addition, with respect to the possible misunderstanding by the reviewer, we would like to clarify that two matrices are compared: |Δβ|MZ twin (329 evCpG for 146 twin pairs) and |Δβ|technical replicates (329 evCpG for 16 technical duplicates). The rather difficult to find technical duplicates derived from 3 twin pairs (one with 4 technical replicates per twin and two with 2 technical replicates per twin) by making all possible combinations. Our approach was described in the initial Supplementary methods and in the legend of the initial figure S10C, but now also in the revised Supplementary methods (lines 68-71, 488-505).*

*Most importantly, the fact that the median of |Δβ| (or where the empirical cumulative density distribution of |Δβ| is equal to 0.5) is 0.0299 for the technical replicates and 0.0353 for the MZ twin pairs means that for 50% of the values in the matrix (2,632 entries out of the 5,264 in technical replicates and 24,017 out of the 48,034 entries in MZ twins) are equal or below 0.0299 and 0.0353, respectively for twins and technical replicates. These apparently small differences are not homogenously distributed across twin/replicate pairs or evCpGs: there are different degrees of similarity between MZ twins and different twin-resolving power between evCpGs. Because of this, by no means it can be interpreted that 50% of evCpGs present |Δβ|MZ twin less than 0.02. However, as a response to this comment, we have now included an additional, more convincing analysis on the same dataset including a comparison with control CpGs (Fig 2), discussed in the revised manuscript (lines 158-164).*

*Also, despite not pursing replication at this stage of the manuscript, the reviewer suggests performing the same discovery analysis on evCpGs in the Danish twin cohort. In our discovery strategy, unrelated individuals are indeed obtained by reshuffling twin pairs in a specific way (see revised manuscript, lines 134-136 and Supplementary material for details, lines 324-328). Of course, we could replicate the discovery on other twin datasets, however all of them contain MZ twins of a wide age range, so replication could simply be due to epigenetic drift effects. This motivated the choice of the E-risk cohort where relatively young MZ twins of almost the same age are available.*

*Finally, as a response to common concerns expressed by the reviewers on this matter, we have now included an extra analysis to confirm the short-term longitudinal stability of evCpGs on the dataset of Zhang et al PLoS One 2015; PMID: 26248206 (Fig 3), also discussed in the manuscript (lines 168-178). We were previously aware of this dataset but did not include it in our original analysis as the data are not available in their raw format (IDAT), which is strictly required for all the extensive quality control analysis we perform. We believe that the degree of QC on this already processed dataset is less thorough than the one we performed on the other employed raw datasets.*

4. Regarding the Gambian data set (line 111), since this is not a twin cohort, it is unclear what point the authors are trying to make.

*Reply: Our aim was to use a large children’s 450K cohort, as young as possible that had been deposited in a publicly available repository in its raw format. We agree that ideally that would have been a twins’ cohort, but unfortunately, to the best of our knowledge, no available dataset meets these requirements. The Gambian cohort was the largest and youngest publicly available dataset we could find that was deposited as raw IDAT files, as a requirement for our extensive quality control. As the reviewer is probably already aware, this condition is rarely met for the Gene Expression Omnibus (GEO) datasets deposited a few years ago. Although the Gambian cohort dataset is not a twin cohort, we strongly believe that it can provide additional insights on the nature of evCpGs, given that we do not expect strong genetic effects, based on the following arguments:*

*• Our discovery strategy is incompatible with strong deterministic genetic effects given that they would strongly fuel |Δβ|unrelated individuals but not |Δβ|MZ twins. If these existed, then the two distributions could never be equivalent.*

*• Children cohorts have barely experienced epigenetic drift/clock yet. Thus, together with the argument above, neither genetics nor epigenetic drift/clock can be the main source of evCpG inter-individual variation in a children cohort.*

*• Strong inter-individual variation on evCpGs is present in the Gambian dataset that includes children of just 2 years old. Given that evCpG methylation variation remains, we concluded that it must have been promoted at earlier stages, such as embryonic development or early life, specifically earlier than 2 years old.*

*This overall argument was already stated in the initially submitted manuscript, but we have further clarified and extended it in the revised manuscript (lines 203-214). Additionally, confirming the results available in the literature, we have also now included another cross-sectional children cohort pursuing to disprove strong epigenetic drift effects between late childhood and adolescence (lines 189-192), further pinpointing the source of the variation to embryonic development and/or early life.*

5. The authors state (line 114) that their observations lead to the conclusion that evCpG stochastic variation is established early in embryonic development. It is entirely unclear how they reached this interpretation.

*Reply: On line 114 of the original manuscript we stated “Thus, these observations led us to the conclusion that evCpG stochastic variation is established early on in (embryonic) development but amplified overtime with epigenetic drift”. With the term “(embryonic) development” under brackets, we aimed to encompass both embryogenesis and development, with the latter understood as early life experiences. In this study, we do not, and cannot, present evidence that can distinguish between embryonic development and early life as defined by occurring at least before the second year of life. We had not attempted to distinguish between the two stages in the manuscript given the peculiarities in the development of the immune system. Due to allotolerance between mother and embryo (tolerance to maternal antigens), a functional immune system cannot be established in utero; hence, the development of the immune system prolongs until early life [1]. This is pertinent given that the tissue employed for discovery is whole blood. In any case, with the format upgrade, we have amended any possible misinterpretation by referring in a literal fashion to early life at all stages of the revised manuscript (examples: lines 34, line 213, etc) and by adding the matter of no distinction in the discussion (lines 382-386).*

6. In particular, it seems they have only considered data from peripheral blood. So 1) there is question of whether these variants might simply reflect interindividual variation in leukocyte composition, and 2) the question of whether these variants are systemic (i.e. consistent across different tissues and cell types).

*Reply: With respect to (1), it is unlikely that whole-blood cell type composition is the main driver of the methylation differences we observe in evCpGs, given that we have corrected for such potential differences in our discovery pipeline applying frequently used published methods for whole-blood cell type composition estimation (e.g. Houseman method), but adapted for our purpose (see Supplementary methods, lines 243-259). Also, with the format update to full article, we have now also included replication in adipose tissue in the revised manuscript (lines 231-245). This provides further evidence that the differences observed cannot be simply due to cell type composition heterogeneities, as adipose tissue is a relatively homogenous tissue, mainly composed by adipocytes with a minor component of endothelial cells and macrophages.*

*With respect to (2), we would like to clarify to the reviewer that we did not use the word “systematic” to refer to “systemic” (which refers to claims being true across every tissue). We used “systematic MZ co-twin epigenetic variation” to refer to twin-to-twin divergence that is frequent/universal across different MZ twin pairs (e.g. frequently discordant). This concept is opposite to non-systematic epigenetic variation that only affects single MZ twin pairs or a small subset of MZ twin pairs, for example due to a pathological discordance between twins, e.g. one twin having a disease while the other does not. We do not suggest universality of evCpGs behavior across human tissues (especially depicted with the newly analyzed data on post-mortem tissues), but all our claims are restricted to whole blood and adipose tissue for which datasets were suitable and available. Thus, to account for the reviewer’s comment and avoid future confusions by the readers, we have avoided the word “systematic” across the revised manuscript.*

7. The authors report from their GO analysis (line 119) that evCpGs are enriched for neurodevelopmental processes. This raises two issues. First, the background set used in in their analyses. Rather than reflecting all HM450 probes, it is apparent from their data that all evCpGs are derived from the small subset of HM450 probes showing interindividual variation of at least 0.2 Beta. Hence, this is the appropriate background set. Also, as described by Gunasekara et al (Genome Biol 2019), there is a tendency for genes expressed in neurons to be large genes. Simply because of this, GO analysis of randomly selected probes will be biased toward large genes (just by chance) and enriched for neurodevelopmental processes. The authors should adjust their GO analyses for gene size. Regarding the enrichment for protocadherin (Pcdh) genes (line 147): Pcdh genes tend to be extremely long, so the same comments re: correcting for gene length are salient.

*Reply: We would like to clarify that in our analysis and manuscript we did not perform or describe a filter for probes showing “inter-individual variation of at least 0.2 Beta”. From now on, we assume that the reviewer refers to the filter of ICC > 0.37 and IQR > 0.07, instead.*

*We agree with the reviewer that the background should reflect the starting point of the discovery, e.g. all sites that had the potential to be significant. For example, as we intended to find markers displaying universal variation between MZ twins, probes located in the Y-chromosome were irrelevant for our purposes, as they would only be valid for male MZ twins. As these were removed straight away at the beginning of the pipeline, these should be excluded from the background (as we did) in order to avoid biasing the enrichment p-value. We however disagree where the discovery starting point lies at. The reviewer suggests that given that we did not perform statistics on the set of excluded non-variably methylated CpGs, these should not be included in the background. On the contrary, we discuss that the selection of variably methylated probes is also part of our discovery phase. Without this stage, CpGs devoid of inter-individual variation and only experiencing measurement error would also come across as significant hits.*

*In practice, our enrichment results reported in the manuscript are still significant regardless of which of the two backgrounds we choose (for all 450K probes, our chosen background of 346,555 and for the 4,652 variably methylated CpGs). Please see Table R1 in the Supplementary cover letter document.*

*However, the strategy of choosing variably methylated CpGs as the background is not free of bias. CpGs that display high IQR also happen to be intermediately methylated. It is more likely for a CpG displaying intermediate methylation to also display differences between MZ twins. Moreover, CpGs displaying high stochastic inter-individual variation (which we search with our statistical approach) also display high non-stochastic inter-individual variation (which we enrich with our filters). Hence, the filtering step in not independent of the statistical testing. Should we then ignore the putative enrichment occurring at this first step of the pipeline by selecting only the set of variably methylated CpGs in the background for GO enrichment?*

*In common EWAS practices, an arbitrary empirical threshold of inter-individual variation is avoided by also testing for significance on CpGs displaying barely any inter-individual variation. However, being susceptible to mostly measurement error makes a CpG’s methylation very unlikely to be associated to any phenotype. These are still included in the background of a GO enrichment analysis as they were statistically tested for association. But, altogether, the EWAS pipeline can be envisioned as a virtual or hypothetical two-step pipeline: 1) does the CpG display inter-individual variation? 2) If yes, does the CpG’s variation associate to the phenotype? The difference between an EWAS and our study is that we cannot measure association directly as there is no phenotype to which you can associate methylation with (e.g. stochastic) and hence, we need to manually perform the first step of filtering probes lacking inter-individual variation in order to avoid the introduction of large amounts of false positives displaying only measurement error. In conclusion, given the close ties between the filtering of probes lacking inter-individual variation and our discovery strategy, we decide to respectfully omit the suggestion of downgrading the background to the set of variably methylated CpGs.*

*Finally, we agree with the reviewer’s comment on adjusting for gene length, hence why we had already corrected for gene length in our initial GO enrichment analysis in the previous version. More specifically, this was performed by setting the argument prior.prob = TRUE on the missMethyl::gometh function, which accounts for the probability of significant enrichment simply due to a higher number of probes per gene. We understand that this was not explicitly explained on the Supplementary methods as initially submitted and we thank the reviewer for spotting this. We have now included it in more detail in the revised Methods (lines 543-544) and Supplementary methods (lines 663-667).*

8. Regarding the external validation experiments (line 157) the authors should explain more clearly why data on only 2 of 7 MZ pairs was included. Otherwise this approach suggests cherry picking.

*Reply: We agree with the reviewer that selecting a reduced number of individuals from a whole pool without a criterion would certainly qualify as cherry picking. However, this is not what we did. A brief explanation on why we selected only 2 out of 7 MZ twins was initially included in the firstly submitted Supplementary methods (line 379-381, original manuscript). As reported by the authors who produced the WBGS data we are using on this study [2], there is a dramatic difference in sequencing coverage between the 7 twin pairs (apparent on Fig S18B, original manuscript). We only included twin pair 2 and 3 as these were the only ones reaching our coverage threshold (>10X) required for implementing our extremely conservative analysis. This means that we had to exclude CpG sites:*

*• with more than 20% methylation differences between strands because that makes them unreliable*

*• falling within the Duke Excluded or DAC Blacklisted Regions, known to yield artefactual high coverage*

*• with coverage ≤10 reads in order to avoid sampling errors and ensure correct quantitative analysis*

*• larger than the per-sample 99.9% quantile of reads in order to filter out possible library PCR artefacts*

*• that were not included in both twins (given that we need to compute |Δβ|).*

*Additionally, for our WBGS analysis we established via simulations our |Δβ| threshold to 0.4 (Supplementary methods, lines 383-400, original manuscript), as differences higher or equal to this threshold are very unlikely to have arisen simply from random sampling only. In order to further investigate this topic, we have extended the analysis to all twin pairs in blood and adipose tissue (Fig 5, Fig S22-23, revised manuscript). Interestingly, twin pairs that do not display significant enrichment in sites with |Δβ| ≥ 0.4 in cPCDH are also those displaying the lowest coverage in this region (Fig 4C, revised manuscript). We consider the threshold of 0.4 as extremely conservative - differences lower than 0.4 can also represent true biological variation but we are less certain whether they could also arise by true sampling error.*

*In the revised manuscript, we have now included detailed explanations on the manuscript itself thanks to the format upgrade to full article (lines 312-337, lines 546-564).*

9. Also, the Busche et al paper provides WGBS data on adipose tissue from the same individuals. Why not analyze data from adipose tissue as well? This would provide some information regarding the systemic nature of svCpGs, as well as the potential for svCpGs to simply reflect interindividual variation in leukocyte composition.

*Reply: We fully understand and share the reviewer’s interest on testing other non-blood tissues. For this reason and thanks to the format upgrade to full article, we have now included in the revised manuscript replication on 450K data concerning adipose tissue as per the reviewer’s suggestion, in addition to an extension of the WGBS analysis to adipose tissue as well. Altogether, we provide convincing evidence of stochastic variation across blood and adipose tissues. We do, however, not claim that such variation is systemic, as we do see strong inter-tissue variation in evCpGs in post-mortem tissue panels. Given that we had initially taken measures for accounting for whole blood cellular composition heterogeneity in the discovery in blood (already discussed at point 6) and we also replicated in adipose tissue, it is extremely unlikely that cell composition imperfections, sub-optimally corrected by our approach, can explain our claims.*

Minor concerns

10. The authors refer several times to "systematic" epigenetic variation. From context, it seems they mean "systemic". If not, I'm not sure what they mean by systematic epigenetic variation. The authors cell type and tissue type as a driver of "systematic epigenetic variation". If they mean systemic, this does not make sense, as systemic variants are, by definition, not tissue specific.

*Reply: We apologize about the confusion that our term “systematic” has caused to the reviewer. As discussed also at point 6, we used “systematic MZ co-twin epigenetic variation” to refer to frequent/universal twin-to-twin divergence (e.g. consistently discordant) in contraposition to twin-pair specific discordance, as for example seen due to a pathology on one of the twins but not the other. All our initial claims in this manuscript were restricted to whole blood but now expanding also to adipose tissue. We have now replaced the word “systematic” with “universal” to avoid such confusion and included replication in 450K data in adipose tissue in addition to extension of the WGBS analysis to adipose tissue.*

11. The authors refer to "in utero stochastic mechanisms promoting epigenetic variation" (line 60). However, the processes they refer to occur prior to implantation, so "in utero" is inaccurate.

*Reply: We have accounted for the reviewer’s comment and have now rewritten this part to avoid such imprecision in the revised manuscript (lines 75-80).*

12. The authors refer to the advantages of studying twins of young age (line 73), but employ a data set on 18 year-old twins. Would not a data set on infant MZ twins be better? This merits comment.

*Reply: We fully agree with the reviewer that if we intend to draw conclusions on embryonic development and early life, a pediatric dataset would be optimal. Specifically, we had acknowledged such limitation in the manuscript by referring to “Given that we used young MZ twins rather than newborns, (…)” (lines 103, original manuscript). However, to our defense, in order to perform our extensive data analysis, we require a large number of MZ twin 450K methylation data in its raw format (IDAT) so that we can perform our deep quality control (superior than standard implementations) and we are unaware of any publicly available dataset that meets the above requirements. We invite the reviewer to suggest a suitable dataset if he/she is aware of. Also, whole blood extraction or adipose tissue biopsy tend to be too invasive for pediatric use; other tissues like epithelial tissue, saliva, blood spots or cord blood are preferred in practice with the risk of lower technical replicability and limitations towards correcting cell compositions heterogeneities. We have greatly expanded this part on this in the revised manuscript (lines 181-188).*

13. I could not find any definition of what "evCpG" stands for.

*Reply: We reported the abbreviation in the dedicated Abbreviation section in the initial manuscript (lines 198-204, original manuscript) as: evCpG stands for equivalently variable CpG between co-twins and unrelated individuals. However, we have now also added a much clearer definition in the main text of the revised manuscript (line 139).*

14. The authors conclude (line 134) that "evCpGs avoid CpGs posing tight functional regulation in blood". It is not clear what this means, or how they arrived at this conclusion.

*Reply: Based on the evCpGs position with respect to nearby genes, we observed an enrichment of CpGs not associated to genes and of CpGs associated to 1st Exon and TSS1500 as well as a depletion of CpGs associated to TSS200 and 5’-UTRs. Hence, this suggests that evCpGs tend to avoid important regions for gene regulation. Also, based on results of the ChromHMM segmentation of the methylome of PBMC, we observed a significant enrichment in states associated to heterochromatin, strongly and weakly polycomb repressed states and a significant depletion in active TSS flanking regions and actively transcribed states. Also, the analysis of the GTEx panel reveals that RNA expression of evCpG-associated genes tends to be low in whole blood. We then conclude that evCpGs concentrate in afunctional regions. However, given that the phrasing in the initial manuscript placed the emphasis on regulation rather than function, this was unclear. This argument is fully included in the revised manuscript with help of the change of format to full article (lines 255-275).*

Reviewer 2

In this study, the authors aimed to identify CpGs that show similar large methylation variation among MZ co-twins and unrelated individuals, not caused by technical noise. Using a consecutive filtering in a discovery dataset of 426 MZ twin pairs they identified 333 CpGs representing true epigenetic variants (evCpGs) and validated those in independent datasets showing that such CpGs occur independent of genetic variation and /technical) measurement error. The authors conclude that these so-called evCpGs demarcate true stable individual epigenetic variants arising during early development which in the case of MZ twins might also serve as biomarkers for epigenetic fingerprinting (e.g., to discriminate between MZ twins in forensics). Oversall this is an important and well conducted analysis and the manuscript is well structured/written. I do have some (minor) concerns/suggestions that should be considered/addressed:

Abstract

1. The authors identified CpGs "displaying similarly large variation among co-twins and unrelated individuals surpassing measurement error", which they call "evCpGs". However, it is not clear why the authors use the term evCpGs. Where is "ev" referring to? This needs a clearer explanation (also in the Introduction).

*Reply: As had been reported in the abbreviations of the initially submitted manuscript, evCpG stands for CpG with equivalent co-twin and inter-individual variation. However, it was pointed out also by other reviewers that this was unclear from the phrasing of the manuscript. Therefore, we adapted the definition and have now explained this better in the revised manuscript (lines 138-140).*

2. The authors could also mention in the abstract that these "evCpGs" might serve as biomarkers for epigenetic fingerprinting, as they have the potential to discriminate between MZ co-twins.

*Reply: We appreciate the reviewer’s comment and have now included it in the revised abstract with the help of the format upgrade to full article (line 50-53).*

(Supplementary) Methods

3. Authors should mention in the Methods section of the main manuscript, that the Methods are described in detail in the Supplementary Methods.

*Reply: We apologize to have missed this before. With the change of format into full-length article, a greater part of the description of the methods has migrated to the main revised manuscript. In any case, we have also now included at various points in Methods that further details are described in Supplementary methods (such as in line 460, line 491, etc).*

4. Page 4, Supplementary Methods "Exclusion of 450K putative noisy probes": Include for each filtering step described in this section the exact number of excluded probes.

*Reply: We have now included the exact numbers in the revised Methods as per reviewer’s request (lines 463-465) and revised Supplementary methods (lines 164-176).*

5. Page 4, Supplementary Methods "450K data preprocessing": Three different methods were selected to normalize the array data (i.e., dasen, stratified quantile normalization, and oob_RELIC_QN_BMIQ). Since at least a dozen of different methods are available to normalize 450K data (Wang et al., Epigenetics, 2015; Shiah et al., Bioinformatics, 2017), the authors should explain why they selected these three methods.

*Reply: Employing several normalization methods is not a standard routine in epigenome-wide studies, but in our study, it was initially included as another quality control step. We did notice strong differences between the methods; thus, to avoid normalization method-specific outcomes, we decided to search for significant results across multiple normalization strategies. Indeed, there are dozens of other normalization schemes but testing all or most of them becomes computationally impractical for large datasets like the ones employed in our study. Given the strong disagreement in the scientific community on which normalization methods is the optimal - some even state that the best choice is dataset-dependent [3] - we selected a subset of three that are very popular and have been implemented in three independent R-packages. Detecting significant results across all three methods increases the confidence on our outcomes. For further reading, details concerning the technical details for each method have now been included in the revised Supplementary methods (lines 205-223).*

6. Page 6, Supplementary Methods "Exclusion of 450K non-variable probes": Explain the intra-class correlation (ICC) coefficient in more detail, because it is now not clear how this coefficient is calculated and why it can be used to exclude non-variable probes.

*Reply: The intra-class correlation coefficient (ICC) measures the proportion of non-technical variance with respect to the total variance. Therefore, an ICC of zero indicates that 100 % of the variance could be explained by technical error (here defined as variance between technical replicates). In the 450K array, probes displaying ICC close to zero are common. This is often driven by very limited inter-individual variation rather than displaying larger technical noise: in the absence of biological variation, 100 % of the variance is technical noise. Hence by providing a filter of ICC, CpGs with high technical noise are discarded as well as those displaying little to no variation. This explanation is now available on the revised methods (lines 469-476) and in the revised Supplementary methods (lines 479-485)*

7. Also in this paragraph, the exact number of excluded probes should be mentioned for each filtering step.

*Reply: As also in response of point 4, we have now included the exact numbers for each filtering step in the revised Supplement methods (lines 463-465).*

8. Page 7, Supplementary Methods "evCpG discorvery": The paragraph starts with "For a total of 4,652 CpGs" …. Does this mean that after filtering only 4,652 CpGs were left from the 480,000? This should be explained.

*Reply: Indeed, the reviewer is right; we excluded CpGs that did not display variation in the 18-year old cohort E-risk study, as defined by empirical cut-offs of IQR of 0.07 and ICC of 0.37. Subsequently, we were left only with 4,652 CpGs to perform our discovery strategy, a number comparable with previously published figures on variably methylated CpGs [4, 5]. This prior selection is of paramount importance for our discovery as CpGs displaying only technical error are expected to display MZ co-twin variation equivalent to inter-individual variation. We now explain this more clearly in the revised manuscript (lines 130-134).*

9. For reproducibility reasons, these 4,652 CpGs should be listed in a Supplementary Table.

*Reply: We have now included the list of 4,652 CpGs in the revised Supplementary file 1.*

Results and Discussion

10. Page 4, line 83: Since not all readers might be familiar with the 450K array, the authors should mention that the 450K array covers ~480,000 CpGs sites.

*Reply: We have now included the quote in the revised manuscript (line 115).*

11. Page 4, line 85-88: The authors should mention here that after strict quality control, prepossessing and exclusion of putative noisy and non-variable probes, 4,652 CpGs were left for the evCpG discovery analysis.

*Reply: We have now stated this fact in a clearer fashion in the revised manuscript (lines 130-134).*

12. Page 5, line 98: Please include after the ICC the reference to Bose et al. (BMC Bioinformatics, 2014), that describes the ICC.

*Reply: This reference has now been included in the revised manuscript as per reviewer’s suggestion (line 133).*

13. Page 5, line 99: Add here the total number of MZ twin pairs of dataset-B from the Danish Twin Registry. Please also add the number of samples (or MZ twin pairs) when you introduce dataset-C, dataset-D and dataset-E in the next paragraph.

*Reply: All numbers have now been included in the revised manuscript (Table 1).*

14. Page 5, line 108-110: Are the observed larger differences in the independent older twin cohort (dataset-D) indeed due to the older age, or could this also be due to a batch effect? Because Figure S1 shows that the "percent of low quality data per sample" is overall higher in dataset-D compared to dataset-A. The authors should also perform this test with dataset-B, because these twins are also older (mean age = 48.4 years) and the "percent of low quality data per sample" seems to be the highest in this dataset.

*Reply: We understand from the reviewer’s comment that batches were understood as datasets; however, throughout the manuscript we have referred to batches as different microarray chips within each study (i.e. the typical meaning of so-called batch effects in microarray-based studies). For the sake of clarity, data quality can be additionally affected by many other factors apart from the chip batch effects, such as: DNA quantity and quality, bisulfite conversion, hybridization, etc. That said, if the data quality for the TwinsUK cohort (Dataset-D) were to be inferior, it would promote a larger |Δβ|MZ twin independently of epigenetic drift. So, it is important to know where the figure of percent of low-quality data per sample derives from. This is computed per sample, by counting the number of CpGs out of the entire 450K array that display a detection p-value higher than 1E-06 or number of beads lower than 3. Such global measurements may have little say on the technical error of particular subsets of CpGs within a given sample. Particularly, TwinsUK is formed only by females. As a result, most of the probes targeting the Y-Chromosome show up as probe failure.*

*As per the reviewer’s request we performed a similar analysis on the Danish Twins Cohort (Dataset-B), which was partly available on the original figure S10C. We have compiled the results from figure S10C and S11A. Please see Figure R1 in the Supplementary cover letter document. As expected, technical replicates display the smallest variation followed by the 18-year old cohort E-risk. Finally, the Danish twin cohort and TwinsUK at the lead seem to display similar variation, but this could simply be explained by the difference in total number of samples between both cohorts. The obtained statistics have been registered in the Table R2, also included in the Supplementary cover letter document.*

*However, TwinsUK and the Danish Twins cohort happen to be the oldest twin datasets and those with the highest low-quality data frequency. In order to understand whether the reported age-dependent increase in variation could be explained simply by a higher frequency of low-quality samples, we examined whether low-quality CpG frequency per sample correlated with higher |Δβ|twin mean across evCpGs in TwinsUK and in the Danish twin cohort. This, however, was not the case as shown in Figure R2 in the Supplementary cover letter document; hence, we concluded that a lower data quality in our older twin cohorts cannot explain the observed increased discordance with age. We have not included his analysis/figures in our manuscript though, given that we were confident that our QC automatically discarded probes with sub-standard quality, but included them here to support our argument.*

15. Page 6, line 122: Explain the abbreviation ME in "ME-like CpGs".

*Reply: The abbreviation is explained in the Background, however, given that it is only used three times in the manuscript, we have now written the full name “metastable epiallele” on all instances, thereby avoiding this abbreviation in the revised manuscript.*

16. Page 7: The evCpGs were enriched at the protocadherin (PCDH) gene cluster at chromosome 5. Large DNA variation within MZ twins at the PCDH cluster has also recently been observed by Souren et al. (Nature Communications, 2019). The authors apparently missed that and should compare their data to this study, particularly discussing the fact that depp sequencing validation showed that some of such variants may be due to technical "artefacts" created on the Illumina arrays.

*Reply: Thank you for pointing out this interesting study. In Souren et al, the authors perform an EWAS for multiple sclerosis (MS) on the 850K platform making use of the discordant twin study design (n = 45 twin pairs) that controls for genetic effects, that resulted in a series of MS-differentially methylated positions (MS-DMPs), some of which were validated with targeted methods. Given that MS-DMPs display differential methylation on MS twins with respect to their control co-twins, hence, they cannot be compared directly to evCpGs that display systematic discordance in all healthy twin pairs without direction. Additionally, these authors describe the discovery of within-pair differentially methylated regions (WP-DMRs) as: i) ≥ 3 CpGs within 1 kb, ii) having a |βMS - βcontrol| > 0.2 and iii) the aberrant methylated co-twin must have a β-value greater than ± 3 standard deviations from the mean. Under these conditions, they report the discovery of 4 within-pair DMRs in the protocadherin gamma gene cluster (PCDHG), all of which are specific to single twin pairs. Towards this, no statistical inference is involved in the definition and hence, discovery by chance is not controlled. Secondly, condition (i) favors regions that are highly covered with probes in the 450K; such as, neurodevelopmental genes. Thirdly, condition (iii) searches for outliers; this could enrich for artefactual (rather than biological) outliers, especially given that their scheme of probe exclusion is rather mild (no mention of cross-reactive probes or SNP-containing probes in the WP-DMR analysis section) and that they do not correct for chip batch effects. Finally, validation via targeted methods was not performed for this particular subset of sites. The authors themselves warn of the limitations on the discussion: “Hence, a contribution of these WP-DMRs to the discordant phenotype cannot be excluded, but since they are mainly pair-specific, these results should be interpreted very cautiously”.*

*In conclusion, given that such results are twin-specific, obtained from MS-discordant MZ twins, not validated on another platform and obtained by a rudimentary statistical methodology for WP-DMR discovery, we have decided not to cite this work as prior evidence of our own findings. In any case, the association with PCDHG was a minor result in the context of this otherwise insightful study. We hope that with the explanations provided here, the reviewer will agree with us on why we did not include a comparison with the data of Souren et al.*

Figures

17. Figure S1: Please standardize the scales of the x and y-axis of these 5 plots, so that the reader is able to compare them. Also increase the font of the labels.

Figure S3 and S4: Please also standardize the scales of the y-axis of these plots, so that the reader can easily compare the figures and observe that the |Δβ| distributions differ between the used normalization methods.

Figure S6: Standardize the y-axis of the three plots.

Figure S8: Standardize the y-axis of the two plots.

*Reply: The scales of all the figures mentioned above have now been standardized in the revised manuscript as per the reviewer’s request.*

18. Figure S9B: The y-axis of figure 9B states "Proportion of samples with detection p-val >0.0001", but the legend states "b. Number of low-quality samples per CpG among significant (evCpGs) and non-significant hits". Please correct this mistake.

*Reply: We thank the reviewer for spotting this mistake, we now have changed the legend to “proportion” instead of “number” in the revised manuscript.*

Reviewer 3

The manuscript by Vidaki and colleagues touches on an interesting, yet vastly understudied biological phenomena, that being interindividual epigenetic variation.

In human population epigenetics, epigenome-wide association studies and various measures of epigenetic age are commonplace, yet the community is only now beginning to realize that changes in variability might constitute yet another measure by which the epigenome (or more specifically, the DNA methylome) is linked to different environmental exposures and/or health outcomes. As such, in principle, the principal idea behind this manuscript is very innovative and timely and I was very excited that this topic was tackled in a systematic way. Specifically, the authors set out to use blood DNAm data from a large cohort of adolescent monozygotic twins to determine 333 CpGs that show a large variation among twins beyond technical noise (they call these evCpGs). They confirm in a number of publically available cohort data that these evCpGs also are highly variable in the blood of unrelated individuals, and identify one particular genomic locus (the protocadherin one) that appears to be particularly susceptible to such variation.

1. For reasons not entirely clear to me, the authors then conclude that the variation at evCpGs is established during embryogenesis rather than resulting from postnatal changes in the sculpting of the DNA methylome.

*Reply: On line 114 of the initial manuscript we stated “Thus, these observations led us to the conclusion that evCpG stochastic variation is established early on in (embryonic) development but amplified overtime with epigenetic drift”. With the term “(embryonic) development” under brackets, we aimed to encompass both embryogenesis and development, with the latter understood as early life experiences. In this study, we do not, and cannot, present evidence that can distinguish between embryonic development and early life as defined by occurring at least before the second year of life. We had not distinguished between the two stages in the manuscript given the peculiarities in the development of the immune system. Due to allotolerance between mother and embryo (tolerance to maternal antigens), a functional immune system cannot be established in utero; hence, the development of the immune system prolongs until early life [1]. This is pertinent given that the tissue employed in the discovery of evCpGs is whole blood. In any case, we have amended any possible misinterpretation by referring in a literal fashion to early life at all stages of the revised manuscript (examples: line 34, line 105, etc.).*

*My initial excitement was greatly dampened upon repeated reading of the manuscript, as in my opinion, it unfortunately falls short on its premise for a number of reasons. As outlined in more detail below, I have three partly overlapping primary concerns that - if addressed properly - will make this a stronger manuscript that might be of interest for a broad readership. Collectively, I feel that some of the conclusions drawn are not entirely supported by the data, or at least leave room for other interpretations. I don't think that this is due to their statistical approach, which as far as I can tell is solid and appropriate for the questions asked.*

2. First, I have concerns as to whether the existing analyses, and the cohorts being used, are appropriate to support the kind of conclusions that the authors are drawing. I note that the authors did not use a single cohort with repeated measures and yet try to make statements about epigenetic drift/variation in a very developmental context.

*Reply: We appreciate the reviewer’s criticism, which we are happy to address and clarify. While we have already included technical replicates (repeated measures of the same samples at the same time point) as part of the Danish Twin Cohort with a dedicated paragraph in the manuscript, we did not include longitudinal samples (repeated measures of the same samples at different time points) as it was beyond our purpose and we do not dispose of the right data to observe the establishment of methylation differences during development and/or early childhood. In an attempt to study short-term stability of evCpGs, we have included an extra analysis on the dataset of Zhang et al PLoS One 2015 (PMID: 26248206) despite initially discarded for being pre-normalized instead of raw. We managed to confirm the short-term longitudinal stability of evCpGs (Fig 3), which we now discuss in the revised manuscript (lines 168-178).*

*Additionally, our claims in the manuscript with respect to epigenetic drift/clock were not meant to be in a developmental context, but restricted only to the age interval present in the cross-sectional Swedish cohort used as our population samples, which excludes development and childhood. To address this point in the revised manuscript, we have now included a cohort of children covering the age range between late childhood and adolescence (lines 189-192). In any case, given that our focus is population epigenomics, we do not consider a longitudinal study design with repeated measures of the same individuals a necessity to study epigenetic drift. We focus in this study on regions susceptible to epigenetic drift across individuals, hence common (e.g. universal) in a population rather than intra-individual longitudinal variation. For our aim, a cross-sectional set-up is valid. We expect that dependence with age (association) reflects the epigenetic clock while increased variance with age (heteroscedasticity) reflects the epigenetic drift. We updated these definitions on our revised manuscript (lines 66-69), based on what has been proposed by Jones et al Aging Cell 2015; PMID: 25913071.*

3. I do appreciate that they use a cohort of much younger children from Gambia in part to address this perspective but I am a bit perplexed that they did not utilize cohorts that allow them to more rigorously test this idea. In my mind, a longitudinal measure of DNAm in twins starting at birth would dramatically strengthen this work. The key question would be the extent to which evCpGs already are present at birth, which would be consistent with the authors' assertion that they are likely arise during embryogenesis.

*Reply: We agree entirely with the reviewer that a longitudinal twin study encompassing from birth to adulthood would be ideal for pinpointing the moment when evCpG methylation establishment and divergence occurs. However, to the best of our knowledge and as previously stated, such dataset does not exist in the literature until now. Given this, the use of our newly included children dataset and the Gambian cohort attempts to reduce the initial time frame being from development to 18 years old, to a narrower one, meaning from development to 2 years old. However, as stated, such experimental design still fails to resolve between embryogenesis and early life, which we now further emphasize in the revised discussion (lines 382-386). Although its interest is doubtless, discriminating between the two scenarios was not one of the objectives of this paper. A more detailed explanation on this point is available on point 1.*

4. I am aware of at least one such dataset that could be utilized here. Martino et al Genome Biology 2013 (PMID: 23697701) performed a longitudinal, genome-scale analysis of DNA methylation in twins from birth to 18 months of age that revealed rapid epigenetic change in early life and pair-specific effects of discordance. While this work was done in buccal epithelial cells, I nonetheless have some difficulties reconciling its messages with the ones presented by Vidaki and colleagues. Further, I was surprised that as far as I can tell, this work was not even referenced here. Analyzing their evCpGs in the Marino data would also allow Vidaki and colleagues to examine more broadly as to whether the conclusions they are drawing from blood hold true for at least one other tissue commonly used in epigenetic studies. This is interesting from a biological perspective and would also make this work more broadly applicable.

*Reply: Although we agree with the reviewer that including the data of Martino et al might at first seem* *like an interesting and reasonable addition to our manuscript, we have decided to respectfully omit this suggestion of the reviewer based on the following arguments:*

*a) Most importantly, discriminating between early life and embryonic development is not one of the aims of our paper. Our already ambitious objective was to identify CpGs displaying universal inter-individual methylation variation, equivalent between young co-twins and unrelated individuals, apparent in the absence of epigenetic drift that cannot be explained simply by technical noise (more details available at point 1).*

*b) In Martino et al, only 3 MZ twin pairs have been assayed both at birth and 18 months. We are hesitant to make conclusions on the longitudinal behavior of our evCpGs across another tissue only based on such small number of samples.*

*c) The data by Martino et al submitted to the GEO database is not in its raw format (IDAT); hence, we are unable to perform our deep quality control, especially important when the datasets are small. This is relevant given that we are particularly troubled by the data quality of Martino et al. Although the authors do not provide the full account of 450K control measures, they do share detection p-value. Employing our current threshold for this measure, we observe that from the 53 samples comprising their dataset that had allegedly past their stringent quality control, 12 are well below our QC threshold (please see Figure R3 in the Supplementary cover letter document). The reason for this dissonance probably lies on the QC measure employed: their sample QC consisted of evaluating whether the average detection p-values across sites was larger than 0.05. Statistically speaking, averaging p-values qualifies as bad practice. In this case, the presence of CpGs with high signal-to-noise ratio will result in p-values very close to zero (for example, multi-locus targeting or cross-reactive probes) and will push the average towards zero even with a large proportion of probe failure as shown below; e.g. the average is a bad centrality measure for highly skewed distributions.*

*Given that the 450K protocol is highly standardized and that the authors employed 1 µg of high-quality DNA, we sought for other explanations for the high density of bad-quality data. At this stage, we dare to suggest the bisulfite conversion kit employed. The so called Methyl EasyXceed kit (Human Genetic Signatures, North Ryde, Australia) has been highlighted in the literature as suboptimal: in [6], it is stated: “Next, DNA recovery was tested on plasma cfDNA from 14 healthy individuals using the 5 top-ranked kits and the MethylEasy Xceed kit (Fig. 4A-E). The latter kit was included to address how a kit with suboptimal performance on artificial DNA samples performed on genuine cfDNA”.*

*d) Especially true for pediatrics, tissues such as whole blood or adipose tissue are rarely available due to the invasiveness of extraction. More importantly, we have not previously examined the behavior of buccal swab tissue methylation on several large independent twin cohorts to thoroughly assess reproducibility and technical noise, the biggest challenges faced in this paper. For example, from [7], they report substantially lower reproducibility for buccal swabs (Table R3 in the Supplementary cover letter document).*

*e) There is a lack of appropriate reference profiles to estimate and correct for cell composition differences in complex epithelial tissues, such as buccal swabs. As a result, a higher inter-individual variation has been described in the literature, which can probably be simply explained by immune cell contamination, ranging from none to 80% [8, 9]. For example in [10], they report a dramatic linear decay in MZ twin genome-wide buccal swab methylation correlation with respect to the absolute within-pair difference predicted percentage of buccal epithelial cells (r = -0.94; p-value = 2.3×10-16), hence proving that epigenome-wide twin divergence can be explained almost completely by cell composition differences in buccal swabs. We are hesitant to make any conclusions on the longitudinal behavior of our evCpGs when we cannot appropriately assess biological and technical variation.*

5. Beyond longitudinal data in twins, I was also surprised that the authors did not carefully examine the developmental dynamics of their evCpGs in ALSPAC, as that cohort has three different DNAm measures from birth to adolescence.

*Reply: We thank the reviewer for suggesting the ALSPAC resource. However, applying for data access, gaining access to the raw data, pre-processing the data and performing the longitudinal data analysis is not achievable within our given timeframe to prepare the revised re-submission. Nevertheless, we will consider analyzing this dataset in the future.*

6. Second, I feel that the work as is, and as I read and understand it, falls short of putting epigenetic variation in the context of the emerging literature, and fails to draw connections to the existing data. Beyond the obvious emission of the Marino work mentioned before, there are a couple of directly relevant studies. For example, while small (10 twin pairs), Zhang et al PLoS One 2015 (PMID: 26248206) might be useful to integrate as it provides 4 DNAm from the same individuals over the course of a year, thus allowing short term assessment of the fluctuations of the evCpGs identified here within a person/twin pair. I would be reassured about the stability of the evCpGs if they showed very high ICC's across the timepoints measured here.

*Reply: We have already addressed this point with our answer to this reviewer’s point 2 (see above). Briefly, we have performed an extra analysis on the dataset of Zhang et al and could show short-term longitudinal stability of evCpGs.*

7. I also would strongly encourage the authors' to put their work in the context of Li et al Int Journal Epidemiology PMID: 29518222), which looked at average DNAm across the lifespan in a number of cohorts, including several twin studies.

*Reply: We thank the reviewer for suggesting this study by Li et al, but we are unsure why the average measurement of methylation across all CpGs in the 450K array would be informative on the behavior of the very restricted subset of our 333 evCpGs. The 450K array is designed by Illumina to focus on functional regions; hence, in our opinion an average DNAm based on 450K data is a biased measure of global methylation. If we have misunderstood the reviewer, we invite him/her to clarify to us what exactly we should compare using the outcomes of this study.*

8. Beyond these directly relevant studies, I am having a hard time reconciling the messages from Vidaki and colleagues with existing studies of DNAm variation in human populations. In part this might be due to definitions and statistical approaches, but I do note that 333 variable CpGs out of 450,000 that are being measured is a very small number. I do realize that the discovery of such CpGs in monozygotic twins is a unique strength, but nonetheless find it hard to compare this to existing data that looked at epigenetic variation, across individuals, both as it relates to the nature of the variable CpGs (beyond the developmental context mentioned before) but also the pure number of such loci.

*Reply: We find it unfortunate that we did not successfully convey our message to the reviewer in our previous version, especially in terms of existing DNAm population variation, and we are happy for the opportunity to elaborate in this response. We do not expect that the number of 333 evCpGs will be necessarily comparable to any figure previously produced in the literature, simply because it reflects a novel set of markers under novel discovery restrictions. For example:*

*a) Some studies exist where thousands of variably methylated CpGs have been reported, simply by setting empirical cut-offs on a given measure of inter-individual methylation variation in a population cohort. These discovered sets include mQTLs, epigenetic clock CpGs, CpG-SNPs that were missed in the quality control, etc. On the contrary, we aimed to restrict to a very specific subset: variably methylated CpGs displaying similar divergence between young MZ co-twins and unrelated individual pairs, that is likely, and very importantly, not due to technical noise or epigenetic drift. To achieve this, as a first step, we excluded CpGs that did not display variation in the 18-year old cohort E-risk study, defined by empirical cut-offs of IQR of 0.07 and ICC of 0.37). Subsequently, we were left with only 4,652 CpGs, a number that is comparable with previously published figures on variably methylated CpGs. Subsequently, as a second step we performed our discovery strategy only on this subset of 4,652 CpGs, as we aimed to characterize only a sub-portion of this inter-individual variation. This selection prior to the discovery is of paramount importance given that CpGs displaying only technical error are expected to also inevitable display equivalent co-twin and inter-individual variation.*

*b) Some studies exist where authors build ACE models where an E component, representing the unique/unshared environment between the MZ twins, is obtained for every CpG in the 450K array. However, and as noted also by the authors themselves [11], unshared environment is confounded with measurement error. This would not be a big issue if CpGs subject to technical noise in a high proportion with respect to total variance were excluded to begin with; but however, such models are fitted to all CpGs disregarding whether they display methylation variation or not. As a result, CpGs that show no epigenetic variation, meaning that every individual has very similar methylation value, seem to be enriched in E-component. These observations should be interpreted very cautiously as a CpG without inter-individual variation is a CpG subject to just measurement error.*

*c) Existing EWA studies focus on variance explained, rather than unexplained. No matter how high the measurement error is compared to the real phenotypically associated change in methylation, it can be detected with the right sample size and power. In our discovery and for our purpose, we do not dispose of any phenotype of interest to measure association with.*

*d) Finally, the aim of this study was not to conduct an exhaustive discovery of all evCpG-like biomarkers but to correctly identify a subset for which we can ensure with high confidence that measurement error is not fully accountable for the observed discrepancies between MZ twins. Our focus was on true positives which might of course result in a certain number of false negatives. We apologize for not making our reasons for a relatively small number of evCpGs clear enough in the previous version, which we now explain in detail in the revised version (lines 351-368).*

9. Work in non-twin cohorts also has suggested that variable CpGs might not only be tissue-specific, but also enriched for being under genetic control.

*Reply: We agree with the reviewer; the focus of this study, however, was on detecting CpGs without deterministic strong genetic effects because we aimed to find CpGs for differentiating between MZ twins. CpGs under genetic control would result in high |Δβ|unrelated individuals, but not in high |Δβ|MZ twins; hence, these two distributions could not be equivalent. As for tissue specificity, we have now extended our analysis to a wide range of tissues in post-mortem samples (lines 217-230) and adipose tissue (lines 231-245); in latter we replicated evCpG behavior, time stability and WGBS validation as described in the revised manuscript (line 328).*

10. At the very least it would be very interesting to examine how their evCpGs compare to variable CpGs identified in community cohorts of children (for example Islam et al Epigenetics and Chromatin 2019, PMID: 30602389)

*Reply: We thank the reviewer for suggesting this study by Islam et al, as it is a unique study that encompasses matched-PBMC and buccal epithelial cells methylation data for pediatric samples. The manuscript of Islam et al. is dedicated to compare inter-individual variation in both tissues with a special emphasis on the genetic component, in order to find inter-tissue informative sites as well as characterizing tissue-dependent differential methylation. As a result, CpGs are classified based on the existence of associated-mQTL, strong correlation or differential methylation between tissues. None of these measures, however, assesses inter-individual variation in a direct fashion; hence, we are unsure how any of these parameters are comparable to the purposes of our work. Nevertheless, we attempted to examine our evCpGs within their data, but we were unable to do so, as the file designated to include such information (Supplementary file 7) is incomplete. We expect that this is due to a technical mistake during the authors’ initial file submission (given its .xls format, the maximum number of possible rows is 65,536 instead of the required 419,507; hence, the information for 353,971 probes is missing). We are hesitant to display the results for only a subset of evCpGs available in the top 65,536 rows.*

11. or in aging human populations (for example McEwen et al Epigenetics and Chromatin 2015*, PMID: 28465725).

* We were unable to locate this publication. There must be a typo: 2017, instead.

*Reply: We thank the reviewer for suggesting this study by McEwen et al 2017 (not 2015 as we believe) in which they examined methylation, epigenetic age and in silico predicted cell composition changes in blood of adults of the Nicoya region of Costa (n1 = 48). This geographic sub-region is known to display particularly high life expectancy compared to the rest of the country. They performed an EWAS against selected control individuals outside the Nicoya region (n2 = 47), harnessing 20 differentially methylated regions (DMRs) containing ≥ 3 CpGs with FDR ≤ 0.05 and |Δβ| between groups ≥ 0.05, and 4 differentially methylated sites (DMSs); 3 of which were validated by pyrosequencing. The authors concluded that non-genetic effects were more likely to drive such differences, as they confirmed similar population structures as estimated with GLINT. However, the number of samples is very small and the obtained FDRs are barely significant. Regarding the 4 DMPs, all of them were excluded in our analysis before discovery (Table R4 in the Supplement cover letter document).*

*With respect to the 20 DMRs, a cg id list has not been made available. We manually extracted all CpGs within the reported DMRs, rising to a total of 101 CpGs. We then counted the number of CpGs that were excluded throughout the different stages of our pipeline (Table R5 in the Supplement cover letter document). We conclude that there is no evidence of confluence between the list of evCpGs and the lists of DMPs/ DMRs reported on this paper and hence, we chose not to cite it.*

12. Third, as written, overall I found the work as presented rather inaccessible except perhaps for the most expert reader. I think there are at least two reasons for this, including the required brevity of a Short Report in Genome Biology but also some apparent confusion about definitions. Specifically, I found it hard to discern what the authors meant with epigenetic drift versus interindividual variation, and whether they considered them distinct, similar or partly overlapping. In fact, some of the concerns I noted earlier might be rooted in my confusion about how exactly the authors used these concepts. To bring more clarity to this important framing issue, I would encourage the authors to at least consider the definition of epigenetic drift put forward by Jones et al Aging Cell 2015 (PMID: 25913071) as I believe it might help to conceptualize what the authors are actually measuring in a more accessible fashion and possibly contrast it from "general" epigenetic variation.

*Reply: By allowance from the Editor, we have now expanded our manuscript from the very space-restricted short report format to the full article format, which provided us with more space to include more detailed explanations. We believe that this has increased the readability of the revised manuscript also to non-specialists. We appreciate the reviewer’s suggestion on the definition of epigenetic drift. We initially considered epigenetic drift simply as change in methylation with respect to time (which would include both epigenetic clock and epigenetic drift as referred by Jones et al), as have previously expressed in the literature [12]. However, we have now employed the suggested definitions in the revised manuscript (lines 66-69) to avoid possible misunderstandings. Additionally, we have also specifically clarified in the manuscript that we consider inter-individual variation as variation of CpG methylation across individuals in a population across ages, sex, genetic background, etc. (lines 65-66).*

*References*

*1. Simon AK, Hollander GA, McMichael A. Evolution of the immune system in humans from infancy to old age. Proc Biol Sci. 2015; 282; doi:10.1098/rspb.2014.3085.*

*2. Busche S, Shao X, Caron M, Kwan T, Allum F, Cheung WA, et al. Population whole-genome bisulfite sequencing across two tissues highlights the environment as the principal source of human methylome variation. Genome Biol. 2015; 16:290; doi:10.1186/s13059-015-0856-1.*

*3. Wang Z, Wu X, Wang Y. A framework for analyzing DNA methylation data from Illumina Infinium HumanMethylation450 BeadChip. BMC Bioinformatics. 2018; 19:115; doi:10.1186/s12859-018-2096-3.*

*4. Gunasekara CJ, Scott CA, Laritsky E, Baker MS, MacKay H, Duryea JD, et al. A genomic atlas of systemic interindividual epigenetic variation in humans. Genome Biol. 2019 20; doi:10.1186/s13059-019-1708-1.*

*5. Garg P, Joshi RS, Watson C, Sharp AJ. A survey of inter-individual variation in DNA methylation identifies environmentally responsive co-regulated networks of epigenetic variation in the human genome. PLoS Genet. 2018; 14:e1007707; doi:10.1371/journal.pgen.1007707.*

*6. Worm Orntoft MB, Jensen SO, Hansen TB, Bramsen JB, Andersen CL. Comparative analysis of 12 different kits for bisulfite conversion of circulating cell-free DNA. Epigenetics. 2017; 12:626-636; doi:10.1080/15592294.2017.1334024.*

*7. Forest M, O'Donnell KJ, Voisin G, Gaudreau H, MacIsaac JL, McEwen LM, et al. Agreement in DNA methylation levels from the Illumina 450K array across batches, tissues, and time. Epigenetics. 2018; 13:19-32; doi:10.1080/15592294.2017.1411443.*

*8. Turinsky AL, Butcher DT, Choufani S, Weksberg R, Brudno M. Don't brush off buccal data heterogeneity. Epigenetics. 2019; 14:109-117; doi:10.1080/15592294.2019.1581592.*

*9. Zheng SC, Webster AP, Dong D, Feber A, Graham DG, Sullivan R, et al. A novel cell-type deconvolution algorithm reveals substantial contamination by immune cells in saliva, buccal and cervix. Epigenomics. 2018; 10; doi:10.2217/epi-2018-0037.*

*10. van Dongen J, Ehli EA, Jansen R, van Beijsterveldt CEM, Willemsen G, Hottenga JJ, et al. Genome-wide analysis of DNA methylation in buccal cells: a study of monozygotic twins and mQTLs. Epigenetics Chromatin 2018; 11; doi:10.1186/s13072-018-0225-x.*

*11. van Dongen J, Nivard MG, Willemsen G, Hottenga JJ, Helmer Q, Dolan CV, et al. Genetic and environmental influences interact with age and sex in shaping the human methylome. Nat Commun. 2016; 7:11115; doi:10.1038/ncomms11115.*

*12. Jones MJ, Goodman SJ, Kobor MS. DNA methylation and healthy human aging. Aging Cell. 2015; 14; doi:10.1111/acel.12349.*

**Second round of review**

**Reviewer 1**

I am generally satisfied with the changes the authors made in response to my concerns. The logic of the paper is now much easier to follow, and the new analyses that were added generally support the authors' claims.

The authors overstate their findings, however, regarding stability of evCpG methylation over time. A major claim of their paper, put forth in both the abstract and the discussion, is that evCpGs may be useful for epigenetic discrimination of MZ twins, for example in forensic applications. This requires temporal stability of differences within MZ twin pairs. However, the only data they provide on this (Fig. 3B) shows that repeat measurements in a pair of MZ twins at 0, 3, 6, and 9 months of age do not cluster by twin. It is tough to imagine how these evCpGs could be used for epigenetic fingerprinting of MZ twins when they cannot be used to discriminate two MZ twins in multiple measurements spanning just a few months. (Also, in this same figure, the multiple measurements of unrelated individuals all do cluster together, undermining the authors' claim that evCpGs are not influenced by genetics.) At a minimum, the authors should temper their statements regarding stability, perhaps posing this question as something that needs to be clarified in future longitudinal twin studies targeted to these regions.

Also, the manuscript could use some English language editing. Examples of inappropriate/confusing word choice include:

Line	phrase

45	To this end

79	precluding the twinning process

88	deceitfully display relative E components

169	methylation differences at evCpGs were superior to measurement error

**Reviewer 3**

I appreciate the great effort that Vidaki and colleagues have put into revising their manuscript. Expanding it from a Short Report to a full-fledged paper has not only allowed them to address a number of important issues from the previous version but to also include more extensive analyses to make their point. That being said, there are still a number of crucial issues that need to be resolved.

In trying to reconcile the authors' responses to my previous comments with the expansion into a full paper, I will first deal with the previous comments that the authors addressed satisfactorily, followed by those that require substantial attention and/or clarification. I realize that perhaps this is an imperfect way of providing feedback and might be somewhat frustrating to the authors. At the same time, it seems to me like the most transparent and constructive way to deal with the complexities of this manuscript and its evolution.

A) Previous Comments that the authors' have adequately dealt with:

Comments 3 and 4 (which were related)

I appreciate the difficulties in finding epigenetic longitudinal twin cohort studies and was not aware of the poor quality of the Marino et al Genome Biology paper. Looking at it now, I have to agree with Vidaki et al in that it is not suitable for the kind of analysis done here.

Comment 5

Having most recently tried to gain access to ALSPAC data and in the process learned about the complexity of the process and the costs involved, I do agree that at this stage, it is not reasonable to ask the authors to pursue this line of interrogation.

Comment 6

The authors have fully addressed my concern.

Comment 10

It is too bad that the supplementary data file for this publication seems to be corrupted. Given the relatively minor nature of this particular comment, I am satisfied with omitting this analysis.

Comment 11

I appreciate the authors' having done the suggested analysis; the negative result is perhaps not entirely surprising given the different approaches and as such, I am satisfied with the response provided by the Vidaki and colleagues.

B) Previous Comments that require relatively minor attention

Comment 1

I am cognizant that perhaps my concern was related to the authors' use of "embryonic" in parenthesis when describing development. While the editing to include early life in the current manuscript is appreciated, I think it is important indeed that the current study design does not allow the authors to make any conclusions about prenatal variability in DNA methylation. Further, while I am not an immunologist, I am not convinced by the argument put forward by Vidaki and colleagues about the fetal immune system being non-functional in utero. According to my immunology friends, their statement about allotolerance is too general and oversimplified, and in fact the immune system of the fetus is perfectly tuned to what it needs to do. Again, this might be an issue that possible can be resolved by a bit more generous and inclusive arguments in the writing by Vidaki et al, but I certainly feel it is an important point that needs to be clarified.

Comments 2 and 12

I am combining these since my remaining concerns are both related to the definition of evCpGs in the context of epigenetic drift. I am frankly confused by the argument made here that epigenetic drift can be looked at outside of a developmental context and without having longitudinal measures in the same person. Further, we already know that epigenetic drift does not necessarily need to happen consistently or in a linear fashion over time, there likely are periods of more or less drift during the human lifecourse. so the short term stability of evCpGs are not surprising. In this context, it is not entirely clear to me what, in the opinion of the authors, the evidence is for these evCpGs actually constituting a domain of epigenetic variability distinct from epigenetic drift. Again, I might continue to misunderstand their argument, but to me bringing more clarity to this issue would be very important and make this work much more accessible, not only for epigenetics experts but even more so for the more general reader. As presented now, I am still left wanting on this important issue. While I do agree with the authors' statement that, epigenetic drift is simply "individual-specific accumulation of stochastic and environmental changes over time", it is not clear how and why ecCpGs differ from this. I am wondering whether this actually is an arbitrary separation made by the authors and in fact, these phenomena don't really need to be distinguished? In other words, can we exclude that evCpGs are simply a subset of epigenetic drift loci that happened to exist early in life?

Comment 7

I am fully aware that the Li et al paper does employ a very different measurement of variable DNA methylation. However, they do investigate the genetic and environmental causes of variation in genome-wide average DNA methylation in a developmental context, thus clearly overlapping at a conceptual level with the current paper. As such, my recommendation would be for Vidaki and colleagues to put their own work in the context of the Li et al paper in the Discussion only.

Comment 8 (this is probably my most pressing remaining concern)

I realize that given the apparent stringency of their filtering process, it is reasonable for Vidaki et al to observe fewer variable CpGs. However, at the same time, I am very much struggling to fully understand how the present analysis is different from what Jonathan Mill and colleagues (PMID: 30091980) did in the exact same e-RISK cohort when characterizing Genetic and Environmental Influences on Variable DNA Methylation Using Monozygotic and Dizygotic Twins. In particular - and this is relevant to a number of my comments - and seemingly in contrast to the work of Vidaki, they document a strong effect of genetics (mQTLs) on variable DNAm. I might have missed something here, but are the authors claiming that the study from Mill lacked in stringency? Whatever the argument might be, I feel this needs to at the very least be addressed constructively in the Discussion.

Similarly, I feel strongly that the authors need to put their work in the context of very prominent existing papers that have documented that a very minor, if any, variation in DNAm can be attributed to environment alone. Specifically, as initially documented by Joanna Holbrook (PMID: 24709820) and further substantiated more recently by Elisabeth Binder (PMID: 31186427), variable regions of DNAm in cord tissue or cord blood/children blood are best explained by a G(enetics)xE(environment) or G only model, but rarely - if ever - by E alone. Are Vidaki et al claiming to have isolated the very small E component contributing epigenetic variation, or are their evCpGs an underappreciated stochastic component not captured by the models applied by these two papers? In any case, I am aware that both Holbrook and Binder used variable methylated regions, as opposed to the single CpGs used by Vidaki et al. Nonetheless, I feel strongly that Vidaki et al need to put their work in the context of these important papers and reconcile the findings. And I will not simply accept the argument put forward by Vidaki numerous times in their rebittal that they simply used much more rigorous normalization than everyone else to skirt this topic.

Comment 9

I do appreciate the argument by Vidaki and colleagues that the twin design largely eliminates the genetic component of epigenetic variation. That being said, especially now with the expanded format, I would recommend for the authors to at least acknowledge in the Discussion that epigenetic variation outside of the monozygotic twin space has been shown in numerous studies to be enriched for CpGs under genetic control (please also note my major point below re. the effects of genetics and/or environment in determining DNAm variation).

As for tissue specificity, again, I am cognizant of the effort the authors have made by including additional tissues in their analysis. However, at the same time I agree with Reviewer 1 that there are a large number of datasets from children available in public databases that report on DNAm in buccal epithelial cells (easily 10x as many as from any other pediatric tissue), and that in many ways these would be ideal to include here. I disagree with the statement by Vidaki and colleagues made in a different part of their rebuttal that there are no proper procedures in place to account for cellular heterogeneity in buccal cells. Both Andrew Teschendorff (PMID: 30504870) and Alicia Smith (PMID: 25355443) have published such algorithms and they are widely used in the community. I personally would love to see the inclusion of buccal cell analyses in this manuscript, as it would possibly make it a much stringer study. At the same time, I am cognizant that this might constitute a large amount of work and the expected outcome might not add much in terms of new information.

**Authors Response**

**Point-by-point responses to the reviewers’ comments:**

Responses to reviewers’ comments

Reviewer 1

I am generally satisfied with the changes the authors made in response to my concerns. The logic of the paper is now much easier to follow, and the new analyses that were added generally support the authors' claims.

1. The authors overstate their findings, however, regarding stability of evCpG methylation over time. A major claim of their paper, put forth in both the abstract and the discussion, is that evCpGs may be useful for epigenetic discrimination of MZ twins, for example in forensic applications. This requires temporal stability of differences within MZ twin pairs. However, the only data they provide on this (Fig. 3B) shows that repeat measurements in a pair of MZ twins at 0, 3, 6, and 9 months of age do not cluster by twin. It is tough to imagine how these evCpGs could be used for epigenetic fingerprinting of MZ twins when they cannot be used to discriminate two MZ twins in multiple measurements spanning just a few months.

*Reply: Indeed, the reviewer is correct: the samples derived from a single adult twin pair from the data of Zhang et al assayed at time points 0, 3, 6 and 9 months apart do not separate in different clusters but rather appear as one single inter-mixed cluster (Figure 3). This seems to suggest that variation occurring between short time periods supersedes co-twin variation, at least in this single twin pair. However, we do not know with confidence whether in this specific twin pair there were differences in the methylation of evCpGs to begin with, as no technical replicates were included at time point zero. Maybe methylation levels were similar to begin with. Additionally, since the twin pair is 26 years old, epigenetic drift is not expected to be a large source of the variation. Both these reasons together could explain such observation.*

*Having said that, we cannot ignore that the raw data of this dataset was unavailable (IDAT files), which is required for all the extensive quality control and preprocessing. As a result, we had to rely on what Zhang et al had originally done in their paper and hence, the degree of control was smaller than in our core datasets. It could well be that by applying our careful pre-processing and normalization, the MZ longitudinal stability incongruence could be eliminated. But we cannot test this hypothesis as the raw data files were not made available. Moreover, in this analysis we used only 296 out of the 333 evCpGs; 37 evCpGs (11.1 %) were excluded by Zhang et al for details we do not know. All these reasons could explain at least partly why we were unable to properly cluster the longitudinal replicates for this particular twin pair.*

*In any case, our longitudinal analysis does not aim to examine whether the differences observed between MZ twins are stable in time (we do not have enough data to do so). Instead, we aim to understand whether the methylation of evCpGs is stable in time, which can be performed on unrelated or twin individuals. Thus, our claims on longitudinal stability of evCpGs pertained unrelated individuals. A total of 28 out of 28 longitudinal (0, 3, 6 and 9 months apart) and technical replicates belonging to 6 unrelated individuals were clustered correctly in whole blood. In adipose tissue, employing the replicated set of evCpGs (n = 154), 55 out of 57 longitudinal replicates (0, 5 and 12 months) belonging to 19 different individuals were clustered correctly. These results supply strong evidence that DNA methylation does not behave erratically at short-time scales. Thus, we believe that our claims on the short-time stability evCpG methylation are not overstated.*

*Finally, although longitudinal stability of MZ twin methylation differences is key for the forensic application, we cannot judge the success of this approach based on a single twin pair anyway. Testing the feasibility of MZ twin epigenetic discrimination in a forensic scenario is out of the scope of this paper; however, this topic is of great interest to us and it will certainly be covered in future follow-ups. In summary, towards incorporating the reviewer’s input to the manuscript, we agree that the limitation of the single MZ twin of Zhang et al should at least be acknowledged in the discussion. Therefore, we have now included this topic in the revised manuscript (lines 177-178, 433-448 with visible tracked changes).*

2. (Also, in this same figure, the multiple measurements of unrelated individuals all do cluster together, undermining the authors' claim that evCpGs are not influenced by genetics.)

*Reply: EvCpGs are chosen to have similar co-twin and inter-individual variation. If evCpGs also display short-time stability, multiple longitudinal measurements of unrelated individuals are expected to cluster together. It is true that genetics can also provide such variation but one cannot use this finding as evidence pointing towards genetics, as it would qualify as fallacy of the converse. Stated differently: clustering of longitudinal replicates of unrelated individuals is not a unique signature of genetics. We would like to kindly remind the reviewer that if genetics were to be a strong source of variation in evCpGs, then it would have supplied large inter-individual variation compared to co-twin variation during our discovery. Hence, our evCpGs would have never reached a significant equivalence p-value, as this was the criteria for the discovery of evCpGs; thus, meaning that our pipeline excluded any CpG under genetic control.*

3. At a minimum, the authors should temper their statements regarding stability, perhaps posing this question as something that needs to be clarified in future longitudinal twin studies targeted to these regions.

*Reply: This has been partially answered at point 1. As per reviewer’s request, we have included in the discussion that further and extensive longitudinal analysis on MZ twins is required, especially before any future practical forensic application can be considered (lines 519-523).*

4. Also, the manuscript could use some English language editing. Examples of inappropriate/confusing word choice include:

Line phrase

45 To this end

79 precluding the twinning process

88 deceitfully display relative E components

169 methylation differences at evCpGs were superior to measurement error

*Reply: To improve clarity, all the highlighted phrases have now been rewritten (lines 45, 79, 88, 171).*

Reviewer 3

I appreciate the great effort that Vidaki and colleagues have put into revising their manuscript. Expanding it from a Short Report to a full-fledged paper has not only allowed them to address a number of important issues from the previous version but to also include more extensive analyses to make their point. That being said, there are still a number of crucial issues that need to be resolved.

In trying to reconcile the authors' responses to my previous comments with the expansion into a full paper, I will first deal with the previous comments that the authors addressed satisfactorily, followed by those that require substantial attention and/or clarification. I realize that perhaps this is an imperfect way of providing feedback and might be somewhat frustrating to the authors. At the same time, it seems to me like the most transparent and constructive way to deal with the complexities of this manuscript and its evolution.

A) Previous Comments that the authors' have adequately dealt with:

Comments 3 and 4 (which were related)

I appreciate the difficulties in finding epigenetic longitudinal twin cohort studies and was not aware of the poor quality of the Marino et al Genome Biology paper. Looking at it now, I have to agree with Vidaki et al in that it is not suitable for the kind of analysis done here.

Comment 5

Having most recently tried to gain access to ALSPAC data and in the process learned about the complexity of the process and the costs involved, I do agree that at this stage, it is not reasonable to ask the authors to pursue this line of interrogation.

Comment 6

The authors have fully addressed my concern.

Comment 10

It is too bad that the supplementary data file for this publication seems to be corrupted. Given the relatively minor nature of this particular comment, I am satisfied with omitting this analysis.

Comment 11

I appreciate the authors' having done the suggested analysis; the negative result is perhaps not entirely surprising given the different approaches and as such, I am satisfied with the response provided by the Vidaki and colleagues.

B) Previous Comments that require relatively minor attention

1. Comment 1: I am cognizant that perhaps my concern was related to the authors' use of "embryonic" in parenthesis when describing development. While the editing to include early life in the current manuscript is appreciated, I think it is important indeed that the current study design does not allow the authors to make any conclusions about prenatal variability in DNA methylation. Further, while I am not an immunologist, I am not convinced by the argument put forward by Vidaki and colleagues about the fetal immune system being non-functional in utero. According to my immunology friends, their statement about allotolerance is too general and oversimplified, and in fact the immune system of the fetus is perfectly tuned to what it needs to do. Again, this might be an issue that possible can be resolved by a bit more generous and inclusive arguments in the writing by Vidaki et al, but I certainly feel it is an important point that needs to be clarified.

*Reply: We agree with the reviewer that the impossibility to distinguish embryonic development from early life was an issue worth mentioning and discussing. Towards this mean, we had already dedicated a paragraph to this point in the discussion in the previous version. As for the peculiarities of development in blood, we appreciate the reviewer’s criticism on our arguments and have clarified this point, both here and in the manuscript. Firstly, we did not intend to claim that the immune system is non-functional in utero. Instead, we stated that the development of the immune system persists beyond birth: it is well established that newborns are deviant in both the innate and adaptive immune systems compared to adults [1-3]. It is also well established that a successful pregnancy requires the correct suppression of any immunological response between the fetus and the mother (a.k.a. Medawar’s hypothesis); this is normally stated as the mother needs to avoid mounting an immunological response against the fetus [4]. However, the alternate is also true: the immune system of the fetus needs to avoid mounting a response against the mother [3, 5]. To this end, both immune systems actively cross-talk to promote a tolerogenic environment. We also stated that the immune system of the fetus cannot fully develop in the womb; to our knowledge, this is also a fair statement. However, it is true that other reasons apart from risking mother-embryo allotolerance can justify the need for post-natal development; for example, the sudden and massive exposure to environmental antigens after birth. Following the reviewer’s suggestions, we have now rewritten this paragraph to reflect the intricacies and hope that we have clarified our arguments (lines 456-466 in the currently revised manuscript with visible tracked changes).*

2. Comments 2 and 12: I am combining these since my remaining concerns are both related to the definition of evCpGs in the context of epigenetic drift. I am frankly confused by the argument made here that epigenetic drift can be looked at outside of a developmental context and without having longitudinal measures in the same person. Further, we already know that epigenetic drift does not necessarily need to happen consistently or in a linear fashion over time, there likely are periods of more or less drift during the human life course. so the short term stability of evCpGs are not surprising.

*Reply: We thank the reviewer for his/her input and we are happy to further clarify this argument. Firstly, we would like to remind the reviewer that the idea of employing a cross-sectional design to study epigenetic drift is not novel: in [6], it is stated “epigenetic drift can be observed generally in cross‐sectional studies, as evidenced by increased discordance between epigenomes with advanced age”; In [7], they employ a very similar strategy to us to identify CpGs displaying increasing variability with age. To discuss this further, we also devised the following thought experiment:*

*In a longitudinal study, let’s assume that an individual begins with methylation status (S0). After time t the methylation status is S(t). Epigenetic drift is simply the evolution of S0 towards new states: S(t) – S0. Elsewise in a cross-sectional dataset, the starting point can be thought of as an ensemble of states (E0) rather than a discrete state like in the longitudinal scenario. As time passes, E0 is let to evolve towards new states. Every individual included in the cross-sectional study, though only sampled at one time point across a wide-age range, can be hypothetically traced back to an initial state included within E0. It is to be expected that the diversity of observed states increases with age: longer times enable the exploration of states that are further away from E0. This can also be stated as the system slowly forgets its initial condition or the methylation distribution increases its entropy with time. However, two cases exist in which a cross-sectional study cannot resolve between two confounded scenarios (for which a longitudinal study can):*

*1) The ensemble of states does not evolve in time (it is bounded, no new states are created). This could either be because (A) the system is jumping between states already included in E0 (qualifying as epigenetic drift) or (B) simply because the state of each individual is stable in time. 2) E0 is complete to begin with: further divergence cannot access any new methylation state because all states are already included in E0. This can be again compatible with both (A) jumping between states or (B) stable-in-time behaviors. Scenario 2) is however unrealistic as no CpG in the 450K approaches this limit. We can prove this point by computing Shannon entropy of the discretized distributions of DNA methylation for each CpG across the population cohort of NSPHS (Dataset-E), and plotting their distribution compared with the maximum entropy possible, correspondent to the discretized uniform distribution (Figure R4). Most CpGs are far from approaching uniformity in DNA methylation in this dataset with mixed ages, genders and unrelated individuals. In any case, the existence of counter-scenarios means that we cannot be sure whether evCpGs not displaying significant increase in DNA methylation variation with age in a cross-sectional study are stable in time or jumping between states; nonetheless, it has no repercussion on evCpGs displaying significant increase in DNA methylation variation: it not possible to observe a CpG with increase variation with age in a cross-sectional study but not on a longitudinal study.*

*Secondly, it does not matter whether epigenetic drift happens smoothly over time or in spurious bursts: in either cases the longer time passes (a.k.a. the older the individual), the greater the chance to observe divergence in methylation. Hence, longer time equates to larger chance to reach states further away from E0 in a cross-sectional design. These can be reached smoothly or via erratic jumps. The exact trend of individual methylation with age (whether linear, with jumps or even cyclic) will of course only be detectable in longitudinal studies and is not intended in this manuscript.*

*With this thought experiment in mind and these arguments, we hope it is now clearer to the reviewer how epigenetic drift can be meaningful in a cross-sectional study. We have also added a short explanation on this topic in the revised manuscript (lines 189-195).*

3. In this context, it is not entirely clear to me what, in the opinion of the authors, the evidence is for these evCpGs actually constituting a domain of epigenetic variability distinct from epigenetic drift. Again, I might continue to misunderstand their argument, but to me bringing more clarity to this issue would be very important and make this work much more accessible, not only for epigenetics experts but even more so for the more general reader. As presented now, I am still left wanting on this important issue. While I do agree with the authors' statement that, epigenetic drift is simply "individual-specific accumulation of stochastic and environmental changes over time", it is not clear how and why ecCpGs differ from this. I am wondering whether this actually is an arbitrary separation made by the authors and in fact, these phenomena don't really need to be distinguished? In other words, can we exclude that evCpGs are simply a subset of epigenetic drift loci that happened to exist early in life?

*Reply: The reviewer considers the possibility that distinguishing epigenetic drift from embryonic development/early life-linked epigenetic variation is an arbitrary separation made by us. If this distinction is artificial, then what makes older MZ twins less alike than younger MZ twins must be an expression of the same phenomenon that makes young MZ twins differ between each other.*

*Through the literature, a strong link has been established between the definition of epigenetic drift and aging. Nonetheless, processes occurring during embryonic development and early life are not necessarily related to aging. For instance, representing the stochasticity in the methylome during development, the agouti mouse is a mouse model in which an intracisternal-A (IAP) murine retrotransposon has been inserted downstream the agouti gene, responsible for the colour of the fur of the mouse. A long-terminal repeat (LTR) within IAP can act as a cryptic promoter that induces the expression of the agouti gene in hair follicle melanocytes, switching synthesis from eumelanin (black) to phaeomelanin (yellow) [8]. Given the inherent variability in the methylation of the LTR of the IAP upstream the agouti gene, genetically-identical mice give rise to fur colours ranging from brown (hypermethylated, agouti repressed) to yellow (hypomethylated, agouti expressed). Intermediate colours correspond to different degrees of mosaicism. Can agouti mice be considered an epitome of epigenetic drift? Or is this inherent variability in the detection and hypermethylation of selfish elements a distinct phenomenon to epigenetic drift? We find hard to consider the fur colour of newborn agouti mice a result of aging. Though the underlying mechanism for the agouti mice fur colour is known, we acknowledge this is not the case for evCpGs.*

*Focusing on evCpGs, what happens when we extend the time period of action of epigenetic drift to embryogenesis and early life? Let’s assume evCpGs are solely an expression of epigenetic drift; e.g. "individual-specific accumulation of stochastic and environmental changes over time". If so, epigenetic divergence is fully dependent on time: the longer time passes, the higher the chance to observe twin-to-twin divergence. If true, the blood of young MZ twin pairs should be more concordant in evCpG methylation than in older pairs. The closer you get to time zero, the more similar MZ twins are. But what is time zero? Our aforementioned definition eludes to mention on what tissue: whole blood does not even exist on the first stages of development. Thus, a first good attempt when using data from whole blood could be to set time zero at the time of establishment of the pool of hematopoietic stem cells (HSC) that will give rise to all blood cells. This way, any change occurring during early life, which happens to succeed this event, can qualify as an expression of epigenetic drift. Also, epigenetic drift would incorporate dynamics occurring in the pool of HSC (which HSC escape quiescence, which HSC cells die, etc). But what about differences in the methylation status of the starting pool of HSC occurring between twinning and the establishment of the pool of HSC? Is this variation also epigenetic drift? To include it as well in the definition of epigenetic drift, time zero has to be moved back to the time of twinning. If so, one will also have to include under the same umbrella any stochastic and environmental change in methylation occurring as a result of asymmetric cell division, differentiation, organogenesis, etc, that also qualify to this definition. The only factor left excluded from this definition of epigenetic drift is genetics itself. Hence, epigenetic drift ends up being a synonym of the environment.*

*In conclusion, when one extends the definition of epigenetic drift to development and early life, it ends up being so general that it loses its relationship to aging. Particularly, claiming that the stochasticity of developmental programme can be part of epigenetic drift is counterintuitive and unnecessary. We are of the opinion that a distinction between the inherent variability of processes that construct a pluricellular biological system and those that maintain it can be made, hence our willingness to retain this distinction in this manuscript.*

*Additionally, the reviewer also asked whether evCpGs are simply a subset of markers susceptible to epigenetic drift early in life. Given the entanglement between epigenetic drift to aging and the short scales of time involved, the option that ageing is the cause is improbable. What makes aging in these loci so much faster than the rest of the genome? Instead, developmental and early life stochastic and environmental noise are more probably involved, based on our data. Under the reviewer’s mindset, many of the aspects we attribute to development fall under his/her rather broad definition of epigenetic drift. Though we do understand the reviewer’s point of view, the heart of the issue lies in the confounding between aging and time: longer time implies stronger effects of aging. However, biological processes by definition evolve in time. Is the evolution of any biological process in time a result of aging? We strongly disagree: aging is the accumulative deterioration of a biological system throughout life. Instead, in development an active programme is ran but cannot be performed with 100 % accuracy; this is very different to the passive attrition of aging. Aging is the result of unfaithful transfer of information or maintenance across cellular generations but not simply the noise resulting from carrying out a complex developmental programme. Aging occurs over long-time scales, while developmental noise occurs over short-time scales.*

*Being pragmatic, this discussion is irrelevant for any practical purpose: this distinction between development/early life and epigenetic drift is overall useful. A common misconception in the field is that the epigenome of young MZ twins is identical at young ages, but mysteriously diverges as a result of time. One of our points in this paper was to show that it is possible to observe differences at younger ages in specific loci, not attributable to what was back then defined as epigenetic drift (the force that makes older twins more different than younger twins). The reality is that the terms aging and epigenetic drift are tightly linked in the literature. While we agree with the reviewer that the concept he/she envisions should have a formal name (we suggest biological decoherence), this does not match with the current definition of epigenetic drift. For all the reasons above, we would like to retain the distinction between epigenetic drift and embryonic development/early life-linked epigenetic variation in this manuscript. To acknowledge the reviewer’s additional concern and to further clarify this point, we have included an additional discussion in the revised manuscript (lines 365-384).*

4. Comment 7 : I am fully aware that the Li et al paper does employ a very different measurement of variable DNA methylation. However, they do investigate the genetic and environmental causes of variation in genome-wide average DNA methylation in a developmental context, thus clearly overlapping at a conceptual level with the current paper. As such, my recommendation would be for Vidaki and colleagues to put their own work in the context of the Li et al paper in the Discussion only.

*Reply: Here, we cannot agree with the reviewer that Li et al covers the topic of variable DNA methylation. Instead, genome-wide average DNA methylation (GWAM) is examined; e.g. averaging the DNA methylation across all CpGs for a given sample. There is no direct relationship between variable DNA methylation and GWAM. In any case, encouraged by the reviewer, we have now re-examined the methods and claims made on the Li et al paper. In the following, we explain, after careful considerations, why we decided not to follow the reviewer’s suggestion to put our work in the context of the Li et al paper in the discussion.*

*Overall, Li et al face a huge heterogeneity in various aspects such as tissues (buccal, whole blood and adipose tissue), sample size (from 28 to 614 samples), number of CpGs employed in the average, populations (Australians, Koreans, British) as well as data QC and preprocessing (Table R6). This is a serious issue given that their analysis aims to borrow information across datasets to construct their models. As for preprocessing, we do not understand why the authors did not put effort into harmonizing all datasets by preprocessing with the same strategy on a pooled dataset, as the raw signals of many of the datasets are available in GEO. Instead, they rather opted for independent pre-processing of the data with different approaches, which to our opinion, increased dramatically the differences between studies. Nonetheless, assuming that independent preprocessing could not be avoided, they should have at least pooled the datasets together to examine dataset-specific batch effects. To showcase to the reviewer how different normalizations can impact their measurements, we examined GWAM mean, standard deviation and correlations between MZ twins in the E-risk cohort across normalizations. As we can see in Table R7, all measures vary with respect to the normalization employed.*

*But foremost, the strong confounding between tissue and time point poses limitations to the certainty of their claims. This is especially severe at PETS: buccal cells (birth and 18 months) compared to all the other time points that are non-buccal cells. In order to ease the critic towards this point, they compare another newborn PETS dataset produced in cord blood: “The correlations in GWAM from cord blood mononuclear cells for PETS newborns were 0.80 (95% CI: 0.75–0.84) for MZ pairs, 0.80 (95% CI: 0.72–0.86) for DZ pairs and 0.80 (95% CI: 0.76–0.84) for twin pairs combined, similar to the correlations in GWAM from buccal cells”. However, the correlations in cord blood were obtained in the 27K microarray instead of the 450K. Therefore, this result is not meaningful unless they previously prove that GWAM in 27K is equivalent to 450K (with 17.6 times smaller number of CpGs). The degree of uncertainty grows even further when we notice that the PETS study samples correspond exactly to those from the Martino et al paper, which we have already established to be of very low quality. This of course explains the lower number of available CpGs in PETS (as observed in the table of the previous page).*

*With respect to the modelling section, we believe there are also serious flaws in the methodology and interpretation of results. For instance, i) the assumptions of the models are not stated or discussed anywhere in the manuscript or ii) no goodness-of-fit (GOF) measure is shown to evaluate how good the models fit the data. As a result, we believe that some of the claims are overstated such as: “we conclude that GWAM is determined before birth, possibly by prenatal environmental factors acting in utero, the effects of which persist during the whole life”. Firstly, assuming that they have a working model that can predict GWAM correlation accurately between any two related pairs, this does not mean that you can predict GWAM levels (it is a different variable). Secondly, even being able to predict something, it does not mean it is determined. Is chronological age determined by the methylation status of Horvath clock’s CpGs? They also state: “We found no difference in MZ and DZ pair correlations at birth in the PETS and therefore no evidence for a role of genetic factors at birth”. We are not surprised given the quality and the sample size of the data at the Martino et al paper. However, this is very unlikely true. Finally, they state: “Our study found that the twin pair correlations decreased in childhood, which suggests that individual environmental effects increase during childhood. This may be due to ‘epigenetic drift’, which is in effect the role of non-genetic factors inducing variation in methylation levels. However, given that the twin pair correlations were relatively stable in adult life, our study provides evidence that ‘epigenetic drift’ may be manifest in early life but not in middle age and beyond“. This sentence is confusing: epigenetic drift, under neutral epiallele selection, should not have any direction in a cross-sectional study (not hypo/hyper-methylating with time) and thus should not impact the average methylation. If it did, it would be behaving like an epigenetic clock (non-neutral epiallele selection); moreover, given the strong link to aging the effects of epigenetic drift are more prominent in adults. How can they claim that epigenetic drift is more prominent in childhood? How do they explain that older MZ twins are more dissimilar than younger ones?*

*For all the above, we remain unconvinced by the claims made by Li et al. Particularly, the limited quality of the data analysis further questions whether any of the outputs of the study can be replicated or should be compared with our own. Together with our prior argument in which we stated that genome-wide scale average methylation has little say on our 333 evCpGs, we prefer not to discuss our results in the context Li et al or to cite this paper.*

5. Comment 8 (this is probably my most pressing remaining concern):I realize that given the apparent stringency of their filtering process, it is reasonable for Vidaki et al to observe fewer variable CpGs. However, at the same time, I am very much struggling to fully understand how the present analysis is different from what Jonathan Mill and colleagues (PMID: 30091980) did in the exact same e-RISK cohort when characterizing Genetic and Environmental Influences on Variable DNA Methylation Using Monozygotic and Dizygotic Twins. In particular - and this is relevant to a number of my comments - and seemingly in contrast to the work of Vidaki, they document a strong effect of genetics (mQTLs) on variable DNAm. I might have missed something here, but are the authors claiming that the study from Mill lacked in stringency? Whatever the argument might be, I feel this needs to at the very least be addressed constructively in the Discussion.

*Reply: In the Mill et al paper, a DNA methylation ACE model is constructed for every marker on the 450K array, utilizing samples derived from MZ and DZ twins. This includes three components A: additive genetics; C: shared environment; E: unshared environment. In an ACE model, it is assumed that MZ twins share A and C and differ in E, while DZ twins share 1/2·A and C and differ in 1/2·A and E. From these assumptions, it is possible to fit a structural model to estimate the relative components A, C and E for each CpG (e.g. varA/vartotal, varC/vartotal and varE/vartotal). Most CpGs in the 450K are depleted of inter-individual variation (always methylated or unmethylated) and hence, are simply left with measurement error (ε). As ε is part of E, this means that most markers in the 450K show E close to one, and A and C close to zero. When one restricts the set of markers to those displaying high inter-individual variation, a higher predominance of markers with high A component is observed: high A-CpGs correspond to those associated to unfiltered genetic artefacts or mQTLs. This does not mean that variable methylation is exclusive to mQTL and unfiltered genetic artefacts. This is actually what we see in our data: from the 4,652 variably methylated CpGs, only 333 (7 %) showed equivalent co-twin and inter-individual variation. This means that 93 % of them possibly have strong genetic effects, but these are not in the focus of this study.*

*That said, the difference in number of variably methylated CpGs depends exclusively on the definition of variable methylation. In Mill et al, the definition of variably methylated CpGs was: “those (CpGs) where the range of DNA methylation values for the middle 80% of individuals was greater than 5%; N = 214,991 (out of 420,857) sites (51.1%)”. This can be written as Q80 – Q20 ≥ 0.05, where Q is quantile function. This definition, like many other definitions of variable methylation, is arbitrary and is not based on any theoretical knowledge but simply on empirical thresholds. We instead used a different definition in our study: Q75 – Q25 ≥ 0.07 in all three normalizations and ICC ≥ 0.37. Together with our rather stringent filtering criteria this resulted in a smaller number of CpGs. To showcase the arbitrariness of the definition of variable methylated CpGs, we quantified the number of CpGs that qualified as variably methylated CpGs by following Jonathan Mill’s et al definition in our datasets with our normalizations (Table R8). If we intersect the vmCpGs across normalizations with the definition of Jonathan Mill et al, we end up with a total of 37,920 CpGs: the degree of confluence between normalizations can be quite limited. In conclusion, the number of variably methylated CpGs depends exclusively on how you define variable methylation.*

*Most importantly, there is little confluence in scope between Mill et al paper and our manuscript: while they aim to characterize all CpGs in the 450K in terms of ACE components, we aim to identify a set of CpGs for which co-twin variation is similar to inter-individual variation beyond technical measurement. We would like to state that Mill et al performed their study under adequate levels of stringency for their purposes. The level of stringency of any analysis though depends on the task in question. They avoided the topic of measurement error; in reality, it was not even assessed on their data, given the lack of technical replicates. In our study on the other hand, we attempt to make this great leap and hence require a level of stringency much greater to that from Mill et al and that of the previous studies. We fully respect their work as they were the first to perform an ACE model on a dataset with homogenous age and moreover, made a huge dataset publicly available without which our research would not be possible.*

*To address the reviewer’s comment, we have now included a short section in the discussion remarking how most tested CpGs pose non-significant p-values and how this relates to mQTLs and the strong influence of genetics in the epigenome (lines 290-294, 385-388).*

6. Similarly, I feel strongly that the authors need to put their work in the context of very prominent existing papers that have documented that a very minor, if any, variation in DNAm can be attributed to environment alone. Specifically, as initially documented by Joanna Holbrook (PMID: 24709820) and further substantiated more recently by Elisabeth Binder (PMID: 31186427), variable regions of DNAm in cord tissue or cord blood/children blood are best explained by a G(enetics)xE(environment) or G only model, but rarely - if ever - by E alone. Are Vidaki et al claiming to have isolated the very small E component contributing epigenetic variation, or are their evCpGs an underappreciated stochastic component not captured by the models applied by these two papers? In any case, I am aware that both Holbrook and Binder used variable methylated regions, as opposed to the single CpGs used by Vidaki et al. Nonetheless, I feel strongly that Vidaki et al need to put their work in the context of these important papers and reconcile the findings. And I will not simply accept the argument put forward by Vidaki numerous times in their rebittal that they simply used much more rigorous normalization than everyone else to skirt this topic.

*Reply: Concerning the prominent existing papers cited by the reviewer, both Holbrook et al (A) and Binder et al (B) papers cover the interaction between genetics (G) and the environment (E) in DNA methylation with similar methodology, although in (A), 450K and in (B), EPIC (850K) data are used. In (A) they selected variably methylated CpGs (vmCpGs) defined as those CpGs with median average deviation (MAD) greater than Q95. In (B), vmCpGs are defined as CpGs with MAD greater than Q90 (again, an arbitrary definition). In both studies, vmCpGs were restricted to those at least another neighbouring vmCpG 1 kb apart from each other (now called variably methylated region). To represent each VMR, the site with the highest MAD was selected (so not really working with regions). In (A), they fitted each VMR-CpG with 39 different models and selected the best with Akaike information criterion (AIC). Models included were of three types: E, G and GxE. Best fits were GxE (~ 75 % vmCpGs) and G only (~ 25 % vmCpGs) but never E alone. In (B), similarly to (A), they tested a wider range of models of four types: G, E, G+E and GxE and used AIC to decide which model fits best each CpG. As a result, best fits across the 450K were GxE (40.6 %), G+E (29 %), G (30 %) and E (0.4 %). Assuming that these percentages were accurate, it would indeed mean that CpGs affected only by E are extremely rare; thus, our hypothesis on evCpGs would be challenged, hence the need to reconcile these findings.*

*However, in both papers a caveat concerning their GxE models can be realized when examining how these were fitted. G variables consisted of the alleles from SNPs in 1 MB in cis from the CpG site. E variables consisted of so-called environmental phenotypes: in (A), these consisted of maternal age, depression score, glucose tolerance, BMI, smoking, concentration of a wide range of metabolites (folate, vitamins B6, B12, pyridoxic acid, EPA, DHA, ferritin, magnesium, iron, copper and zinc), birth weight, gestational age and parity. In (B), environmental phenotypes included maternal age, hypertension, pre-pregnancy BMI, anxiety, depression score, betamethasone intake, delivery mode, gestational diabetes, oral glucose tolerance test and parity.*

*According to our view, this definition of E does not match in any way the definition of E in an ACE model. In an ACE model, the covariance between MZ and DZ twins is employed to segment the total variance into A, C and E. As a result, VarianceA + VarianceC + VarianceE = total variance. Elsewise, in these GxE models, the total variance is segmented into VarianceG + VarianceE + VarianceInteraction(G,E) + unexplained variance. As a result, stochastic components, measurement errors and any other source of variation that does not follow the list of phenotypes supplied end up in the unexplained variance term. This is an issue since the percentage of unexplained total variance for their top-ranking models was never determined and could well be a larger percentage than the variance explained. If a big percentage of the variance remains unaccounted for, there is still room for other unknown variables.*

*Which brings us to the second point: these GxE models highly depend on the variables included in the model. We are not convinced to what extent such list of variables correctly model the totality of the environment. Are the authors claiming that the insurmountable complexity of the womb’s environment can be summarised with 19 (A) or 10 (B) variables? For example, it is known that the concentration of methyl donors can have a great impact in the methylation status of some loci, at least in rodents [9]; however, maternal concentration of methyl donors such as methionine, betaine or choline are not included as environmental variables in these studies. We also dispute the relevance of some of the variables included. Especially given that no critical assessment of the variables included in the models was formulated. Finally, the tissue employed in these studies is cord blood. We have already established that our experimental design cannot separate embryonic development from early life. Cord blood will certainly be devoid of early life variation, a potential source of variation in evCpGs.*

*For all of the above arguments, we have reconciled our findings with those from the aforementioned papers. To address the reviewer’s suggestion, we have now included a short section in the discussion about GxE models, their limitations and how they relate to this work (lines 405-421).*

7. Comment 9: I do appreciate the argument by Vidaki and colleagues that the twin design largely eliminates the genetic component of epigenetic variation. That being said, especially now with the expanded format, I would recommend for the authors to at least acknowledge in the Discussion that epigenetic variation outside of the monozygotic twin space has been shown in numerous studies to be enriched for CpGs under genetic control (please also note my major point below re. the effects of genetics and/or environment in determining DNAm variation).

*Reply: We do not claim that most epigenetic inter-individual variation is not associated to CpGs under genetic control. Actually the contrary, our study is a living testament of this fact: from the 4,652 variably methylated CpGs (according to our definition), only 333 (7 %) showed equivalent co-twin and inter-individual variation; this means that 93 % of variably methylated CpGs potentially display genetic effects. In line with the reviewer‘s position, our paper showcases that genetic effects rule on variably methylated CpGs. Also, in our introduction we present genetic variation as a key variable in inter-individual variation. In any case, following the reviewer’s recommendation and aiming to further clarify this topic, we have now also included a clear statement in the discussion (lines 385-388).*

8. As for tissue specificity, again, I am cognizant of the effort the authors have made by including additional tissues in their analysis. However, at the same time I agree with Reviewer 1 that there are a large number of datasets from children available in public databases that report on DNAm in buccal epithelial cells (easily 10x as many as from any other pediatric tissue), and that in many ways these would be ideal to include here. I disagree with the statement by Vidaki and colleagues made in a different part of their rebuttal that there are no proper procedures in place to account for cellular heterogeneity in buccal cells. Both Andrew Teschendorff (PMID: 30504870) and Alicia Smith (PMID: 25355443) have published such algorithms and they are widely used in the community. I personally would love to see the inclusion of buccal cell analyses in this manuscript, as it would possibly make it a much stringer study. At the same time, I am cognizant that this might constitute a large amount of work and the expected outcome might not add much in terms of new information.

*Reply: We fully understand and share the reviewer’s interest on testing other non-blood tissues, especially buccal epithelial cells. However, based on our results on post-mortem tissues, we cannot automatically assume that the list of evCpGs can be transported to other tissues. Therefore, replication needs to be performed on any tissue that one wishes to incorporate, as we did on adipose tissue. In order to perform our pipeline on buccal cells, we need data on MZ twins. Although buccal cell is a common tissue in the literature, we have not succeeded in identifying any single 450K dataset in public repositories derived from buccal epithelial cells or saliva from MZ twins, apart from Martino et al dataset which we have established already to be of low quality. We did, however, find a 27K dataset (GSE58045) but downgrading to this technology solely for this tissue seems unreasonable and may not be comparable. The literature though does show promising non-publicly available datasets such as [10-12]. However, to demand and process data access for such extensive analysis is out of the scope of our study at this advanced stage of our submission.*

*In conclusion, given that we do not have the data to perform a replication in buccal and that this would not add much in terms of new information, we are unable to include buccal cell analyses in this manuscript. Finally, we acknowledge that we were not aware of the cited methods for estimating blood cell infiltration on buccal epithelial cell samples and we thank the reviewer for letting us know about them. We have corrected the manuscript for any imprecision concerning cell composition heterogeneity procedures in buccal epithelial cells and saliva (lines 243-244, 477).*

*References*

*1. Georgountzou A, Papadopoulos NG. Postnatal Innate Immune Development: From Birth to Adulthood. Frontiers in Immunology. 2017; 8; doi:10.3389/fimmu.2017.00957.*

*2. Adkins B, Leclerc C, Marshall-Clarke S. Neonatal adaptive immunity comes of age. Nature Reviews Immunology. 2004; 4:553-564; doi:10.1038/nri1394.*

*3. Simon AK, Hollander GA, McMichael A. Evolution of the immune system in humans from infancy to old age. Proc Biol Sci. 2015; 282; doi:10.1098/rspb.2014.3085.*

*4. Veenstra van Nieuwenhoven AL, Heineman MJ, Faas MM. The immunology of successful pregnancy. Human Reproduction Update. 2003; 9:347-357; doi:10.1093/humupd/dmg026.*

*5. McGovern N, Shin A, Low G, Low D, Duan K, Yao LJ, et al. Human fetal dendritic cells promote prenatal T-cell immune suppression through arginase-2. Nature. 2017; 546:662-666; doi:10.1038/nature22795.*

*6. Jones MJ, Goodman SJ, Kobor MS. DNA methylation and healthy human aging. Aging Cell. 2015; 14; doi:10.1111/acel.12349.*

*7. Slieker RC, van Iterson M, Luijk R, Beekman M, Zhernakova DV, Moed MH, et al. Age-related accrual of methylomic variability is linked to fundamental ageing mechanisms. Genome Biol. 2016; 17:191; doi:10.1186/s13059-016-1053-6.*

*8. Morgan HD, Sutherland HG, Martin DI, Whitelaw E. Epigenetic inheritance at the agouti locus in the mouse. Nat Genet. 1999; 23:314-318; doi: 10.1038/15490.*

*9. Wolff GL, Kodell RL, Moore SR, Cooney CA. Maternal epigenetics and methyl supplements affect agouti gene expression in Avy/a mice. The FASEB Journal. 1998; 12:949-957; doi:10.1096/fasebj.12.11.949.*

*10. van Dongen J, Ehli EA, Jansen R, van Beijsterveldt CEM, Willemsen G, Hottenga JJ, et al. Genome-wide analysis of DNA methylation in buccal cells: a study of monozygotic twins and mQTLs. Epigenetics Chromatin 2018; 11; doi:10.1186/s13072-018-0225-x.*

*11. Odintsova VV, Hagenbeek FA, Suderman M, Caramaschi D, van Beijsterveldt CEM, Kallsen NA, et al. DNA Methylation Signatures of Breastfeeding in Buccal Cells Collected in Mid-Childhood. Nutrients. 2019; 11; doi:10.3390/nu11112804.*

*12. Kurushima Y, Tsai PC, Castillo-Fernandez J, Couto Alves A, El-Sayed Moustafa JS, Le Roy C, et al. Epigenetic findings in periodontitis in UK twins: a cross-sectional study. Clin Epigenetics. 2019 11; doi:10.1186/s13148-019-0614-4.*
